# Artery tertiary lymphoid organs encode a pathogenic high-affinity autoantibody−autoantigen pair in atherosclerosis

**DOI:** 10.1038/s44161-026-00864-w

**Published:** 2026-09-07

**Authors:** Chuankai Zhang, Xi Zhang, Yi Ran, Zhihua Wang, Liping Li, Shu Wang, Junjie Zheng, Yixin Zhang, Ting Sun, Yutao Li, Shu Lu, Mingyang Hong, Zhe Ma, Sabine Steffens, Michael Hristov, Xavier Blanchet, Jingpu Zhu, Xinwen Dou, Xinyi Deng, Klaus Dornmair, Desheng Hu, Shibojyoti Lahiri, Axel Imhof, Nadja Sachs, Lars Maegdefessel, Jingyi Xiao, Jianning Zhang, Yan Wang, Hua Hong, Livia Habenicht, Xinyu Weng, Junbo Ge, Christian Weber, Donato Santovito, Rachael Bashford-Rogers, Sarajo K. Mohanta, Klaus Ley, Andreas J. R. Habenicht, Changjun Yin

**Affiliations:** 1 Division of Vascular Surgery, The First Affiliated Hospital of Sun Yat-sen University, Guangzhou, China; 2 Institute for Cardiovascular Prevention (IPEK), LMU University Hospital, LMU Medizin, Ludwig-Maximilians-Universität München, Munich, Germany; 3 Institute of Precision Medicine, The First Affiliated Hospital of Sun Yat-sen University, Guangzhou, China; 4 Department of Oncology, Cancer Center, The First Affiliated Hospital of Sun Yat-sen University, Guangzhou, China; 5 Institute of Intelligent Sport and Proactive Health, Department of Health and Physical Education, Jianghan University, Wuhan, China; 6 DZHK (German Center for Cardiovascular Research), partner site Munich Heart Alliance, Munich, Germany; 7 Institute for Diabetes and Cancer, Helmholtz Zentrum Munich, Neuherberg, Germany; 8 Institute of Clinical Neuroimmunology, University Hospital, LMU, Munich, Germany; 9 Munich Cluster for Systems Neurology (SyNergy), Munich, Germany; 10 Department of Integrated Traditional Chinese and Western Medicine, Union Hospital, Tongji Medical College, Huazhong University of Science and Technology, Wuhan, China; 11 Molecular Biology, Biomedical Center (BMC), LMU Medizin; Ludwig-Maximilians-Universität München, Planegg-Martinsried, Germany; 12 Core Facility Protein Analytics; Biomedical Center (BMC), LMU Medizin; Ludwig-Maximilians-Universität München, Planegg-Martinsried, Germany; 13 Department of Vascular and Endovascular Surgery, TUM University Hospital, Technical University Munich, Munich, Germany; 14 Institute of Molecular Vascular Medicine, TUM University Hospital, Technical University Munich, Munich, Germany; 15 Health Management Center, The First Affiliated Hospital of Sun Yat-sen University, Guangzhou, China; 16 Klinik und Poliklinik für Innere Medizin II, Klinikum rechts der Isar, TUM, Munich, Germany; 17 Department of Cardiology, Zhongshan Hospital, Fudan University, Shanghai Institute of Cardiovascular Diseases, Shanghai, China; 18 State Key Laboratory of Cardiovascular Diseases, Zhongshan Hospital, Fudan University, Shanghai, China; 19 NHC Key Laboratory of Ischemic Heart Diseases, Shanghai, China; 20 Key Laboratory of Viral Heart Diseases, Chinese Academy of Medical Sciences, Shanghai, China; 21 National Clinical Research Center for Interventional Medicine, Shanghai, China; 22 Department of Biochemistry, Cardiovascular Research Institute Maastricht (CARIM), Maastricht University, Maastricht, The Netherlands; 23 Institute for Genetic and Biomedical Research (IRGB), Unit of Milan, National Research Council, Milan, Italy; 24 Wellcome Centre for Human Genetics, Oxford, UK; 25 Department of Biochemistry, University of Oxford, Oxford, UK; 26 Oxford Cancer Centre, Oxford, UK; 27 Easemedcontrol R&D, Munich, Germany; 28 Immunology Center of Georgia (IMMCG), Augusta University, Augusta, GA USA; 29 Department of Cardiology, The Affiliated Nanjing Drum Tower Hospital of Nanjing University Medical School, Nanjing, China; 30 Nanjing Key Laboratory for Cardiovascular Information and Health Engineering Medicine, Institute of Clinical Medicine, Cardiovascular Medical Center, Nanjing Drum Tower Hospital, Medical School, Nanjing University, Nanjing, China; 31 NHC Key Laboratory of Assisted Circulation and Vascular Diseases, Sun Yat-sen University, Guangzhou, China

**Keywords:** Atherosclerosis, Inflammation, Autoimmunity, Inflammatory diseases

## Abstract

Artery tertiary lymphoid organs (ATLOs) emerge in atherosclerosis, which is a chronic inflammatory artery disease with an autoimmune component. However, whether disease-relevant autoimmune B cells emerge in ATLOs remains unknown. In this study, we isolate germinal center (GC) B cells from ATLOs and lymph nodes from healthy and atherosclerosis-burdened mice, expression clone 60 autoantibodies and screen them for arterial wall reactivity. ATLO GC B cell-derived autoantibodies skew to atherosclerosis-relevant autoantigens versus their counterparts in lymph nodes of both genotypes. One ATLO GC B cell-derived autoantibody (termed A6) binds to histone 2B (H2B) with high affinity. Both vaccination with H2B and adoptive transfer of A6 accelerate atherosclerosis, revealing a pathogenic autoantibody−autoantigen pair. Mechanistically, ATLOs specifically show both distorted B cell activation and immune tolerance checkpoint-regulating gene expression profiles. In a human cohort, circulating anti-H2B antibody titers positively correlate with aortic calcification in humans. We suggest that ATLOs harbor a dysregulated immune tolerance environment permissive for autoreactive B cells that express pathogenic autoantibodies promoting atherosclerosis.

## Abstract (web summary)

By combining single-cell transcriptomic and B cell receptor sequencing of germinal center B cells within artery tertiary lymphoid organs, Zhang, Zhang, Ran, Wang et al. identify and clone an autoreactive monoclonal antibody A6 targeting histone 2B (H2B) and demonstrate that both H2B vaccination and adoptive transfer of the anti-H2B A6 monoclonal antibody significantly accelerated atherosclerosis progression.

## Main

Atherosclerosis constitutes the major underlying disease pathology of heart attacks and strokes^1,2^ and is well recognized as a chronic inflammatory disease. However, its autoimmune disease etiology remains incompletely defined^3–6^. Previous data have shown atherosclerosis-associated autoreactive T cells^3,7^ and autoreactive B cells^4,5^. Multiple autoantigens and autoantibodies have been identified: monoclonal autoantibodies against oxidized low-density lipoprotein (oxLDL) or aldehyde dehydrogenase 4A1 (ALDH4A1) have atheroprotective effects and are of low or undefined affinity for their cognate antigens^8–10^. IgM oxLDL antibody titers negatively correlate with atherosclerotic cardiovascular disease (ASCVD) severity in humans^11^. Many autoantibodies may arise from peripheral naive B cells that escaped central tolerance and express autoreactive B cell receptors (BCRs) but do not trigger autoimmune disease^12^. Polyclonal antibodies to β2GPI^13^, GRP78 (ref. ^14^) or CXCR3 (ref. ^15^) promote atherosclerosis, but their affinities to the respective self-antigens have not been determined. Monoclonal antibodies to Hsp60 (ref. ^16^) and Hsp70 (ref. ^17^) were induced by vaccination and then were shown to exacerbate atherosclerosis. However, none of these studies identified an endogenous autoantibody−autoantigen pair with defined specificity and affinity. Autoimmune diseases are defined by the Witebsky−Rose criteria^18^, which require the identification of pathogenic autoantigens and their related high-affinity pathogenic autoantibodies that form autoimmune disease-causing pairs^18,19^. These criteria have not been met for atherosclerosis, and autoimmune disease societies do not list atherosclerosis as an autoimmune disease or an autoimmune disease-related condition. Recent epidemiological studies have revealed that 19 autoimmune or autoimmune-related diseases confer risks for cardiovascular diseases, including atherosclerosis^20^. Although the underlying mechanisms by which autoimmune diseases confer risk for ASCVD remain unclear, breakdown of tolerance to self is a common denominator in many autoimmune diseases. Another prominent accelerator of ASCVD is immune checkpoint blockade in cancer treatments, which unleashes autoimmunity that manifests itself in exacerbated ASCVD^21,22^. These recent studies highlight the importance of delineating autoimmune traits in atherosclerosis to understand the mechanisms underlying the relationships among the immunology of cancers, autoimmune diseases, chronic inflammatory diseases and atherosclerosis.

Tertiary lymphoid organs (TLOs) in general and ATLOs in particular, unlike the stereotyped secondary lymphoid organs (SLOs), emerge exclusively in various diseased adult tissues in response to chronic inflammation, such as in cancers, autoimmune diseases, transplant rejection, infectious diseases and cardiovascular diseases^23–29^. Curiously, clinical correlation studies of the incidence of TLOs have shown beneficial outcomes in most cancers and many infectious diseases^24^ but unfavorable outcomes in most autoimmune diseases, transplant rejection and atherosclerosis^26,30,31^. However, the functional impacts and underlying mechanisms of action of TLOs on most of these diseases largely remain unknown. Previous work has shown clonal expansion of T cells in ATLOs of mice with atherosclerosis, indicating a T cell autoimmune component^7^. ATLOs emerge in mouse and human atherosclerosis in disease-relevant segments of the arterial tree, including coronary and carotid arteries and the abdominal aorta^27,29,32^. Their advanced stages contain activated B cell follicles with GCs and prominent plasma cell (PC) niches^29,33^. Thus, we suspected that ATLOs may participate in B cell autoreactivity and autoantibody affinity maturation in atherosclerosis^33^. Autoreactive B cells are normally kept under control by highly efficient peripheral tolerance checkpoints^34–36^. When one or more tolerance checkpoints are compromised, autoimmune disease can result^37^. Here, we focused on the humoral aspect of atherosclerosis by studying B cell and PC transcriptomes and possible tolerance checkpoint defects. In GC B cells from ATLOs (and lymph node controls), we determined immunoglobulin light and heavy chain pairs by combining single-cell RNA sequencing and single-cell BCR sequencing. To test the relevance of these BCRs, we expression cloned their corresponding autoantibodies, screened them for arterial wall and plaque reactivity and identified one pathogenic high-affinity monoclonal autoantibody and its autoantigen. Identifying relevant high-affinity autoantibodies that exacerbate atherosclerosis and their corresponding autoantigen(s) would strongly support an autoimmune etiology of atherosclerosis.

## Results

### Approach to explore B cell immunity in ATLOs

**Extended Data Fig. 1:**
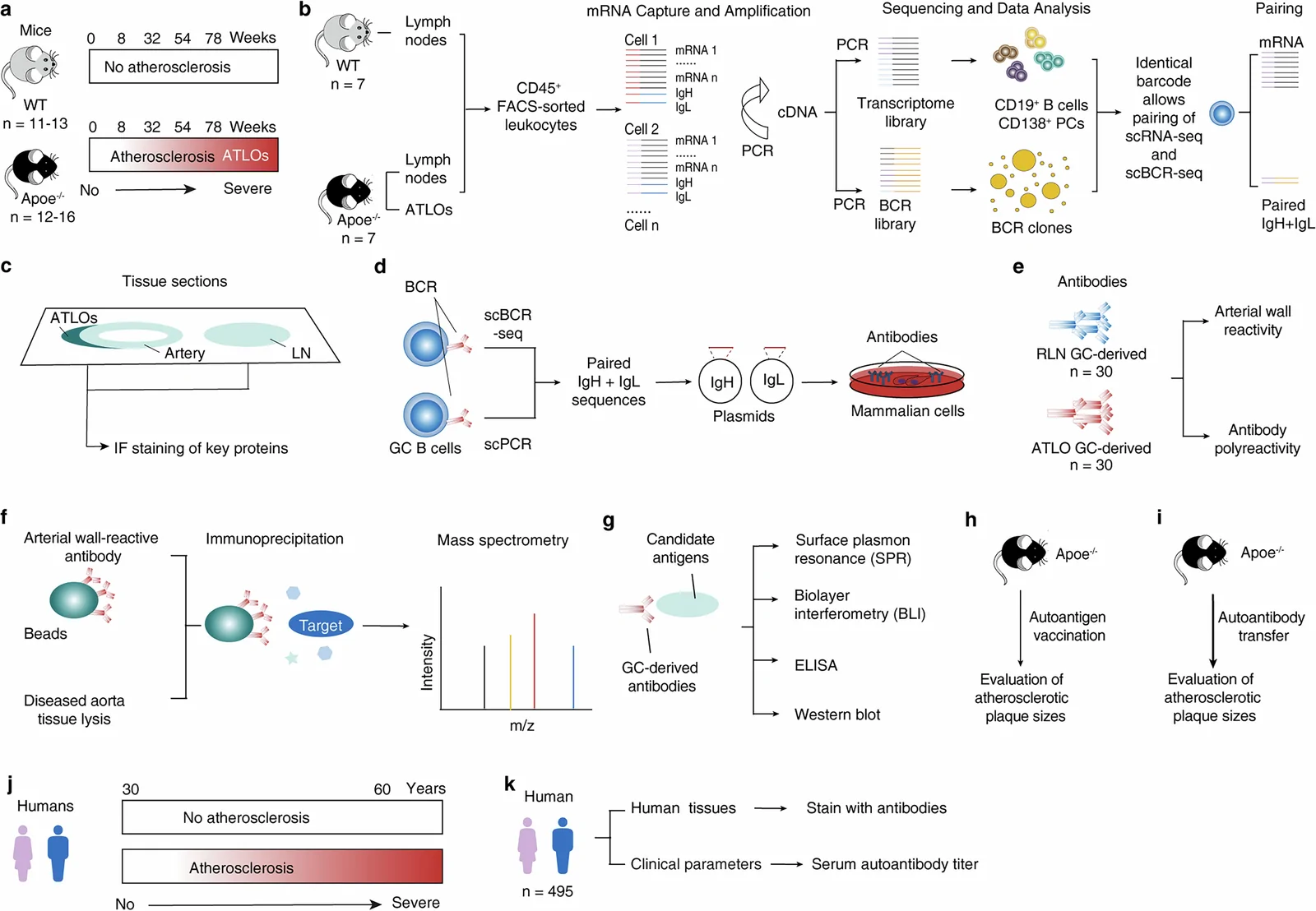
Cross-tissue and cross-genotype strategy to study B cell immunity in ATLOs in atherosclerosis. (**a**) Mouse models of atherosclerosis during aging; B-cell immunity was examined in WT or hyperlipidemic atherosclerosis-prone Apoe^−/−^ mice during aging. (**b**) ScRNA-seq/scBCR-seq pairing of CD45^+^ FACS-sorted leukocytes using droplet barcoding to obtain transcriptomes and paired IgH and light chain (IgL) in single B-cells; to identify expression of differential transcript profiles in multiple B-cell or PC subsets of single clonally expanded vs single nonexpanded subsets, pairing of scRNA-seq/scBCR-seq was employed as described under Methods; CD19^+^ B cells for all non-plasma cell (PC) B cells or CD138^+^ for all PCs were identified by transcriptome analyses from WT, Apoe^−/−^ RLNs and ATLOs. (**c**) Immunohistochemical staining; selective staining of key proteins in mouse or human tissues were performed to identify immunoreactivity of newly observed autoantibodies. (**d**) Expression-cloning of autoantibodies in mammalian cells *in vitro*; paired IgH + IgL sequences of GC B-cells were obtained by scPCR-seq/scBCR-seq, followed by cloning of the paired BCR sequences into expression plasmids and transfection and protein expression of the BCRs in chinese hamster ovary cells or in human embryonic kidney 293 cells. (**e**) Arterial wall and broad tissue reactivity of GC B-cells and examination of antibody polyreactivity vs oligoreactivity; GC-derived antibodies of WT, Apoe^−/−^ RLNs and ATLOs were examined by fluorescent immunohistochemistry in arteries; cloned antibodies were examined for polyreactivity vs oligoreactivity towards insulin, double-stranded DNA and lipopolysaccharide as described in Methods. (**f**) Identification of autoantigen reacting against A6; autoantigen candidates were identified using protein-A/G beads loaded with A6 and diseased mouse aorta lysates to obtain bound antigen-antibody complexes followed by elution of bound antigens and subsequent mass spectrometry analyses of eluted proteins. (**g**) Characterization of autoantibody-autoantigen interactions; following western blots utilizing diseased aorta lysates and expression-cloned antibodies, autoantigen-antibody interaction assays included surface plasmon interferometry (SPR) and biolayer interferometry (BLi); enzyme-linked immunosorbent assays (ELISA) and western blots. (**h**) Identification of histone 2B (H2B) as an autoantigen and determination of its antigenicity and pathogenicity by vaccination; H2B was used as a vaccine followed by determination of its effect on atherosclerosis progression to determine its pathogenicity. (**i**) Identification of A6 autoantibody pathogenicity in adoptive transfers; one of the high affinity autoantibodies, that is the ATLO-GC-derived A6 antibody, was transferred to young or adult Apoe^−/−^ mice under different dietary conditions followed by atherosclerosis measurements. (**j**) Human tissues were evaluated using expression-cloned antibodies and a human cohort aged 30-70 years was studied for circulating anti autoimmune antibodies. (**k**) Translation of mouse data in human organ sections and determination of total circulating anti H2B autoantibodies in a human cohort; human tissue banks were used for atherosclerotic plaque tissue sections to examine reactivity of A6 autoantibody in diseased arteries; in a human cohort of 495 individuals of the general population who underwent preventive health screening, total circulating anti H2B antibody titers were examined to determine their association with cardiovascular diseases.

Selective interference into TLOs to examine their specific functions without changing SLOs has not been a viable approach in the past, because molecules regulating formation of the stereotyped SLOs during embryonic development and the non-stereotyped disease-associated TLOs in adulthood largely overlap^38,39^. To sidestep these obstacles, we implemented a cross-tissue and cross-genotype approach in mice. This strategy was designed to avoid the need to directly interfere into either SLOs or TLOs^38^. Here, we focused on B cell immunity as we had observed follicular dendritic cell (FDC) networks in GCs of activated B cell follicles in advanced stage III ATLOs, indicating that ATLOs are home to antigen-specific B cell immune responses^7,26,29^. Our study design included mapping B cell subsets and their BCR repertoires across lymph nodes and ATLOs by pairing of single-cell RNA sequencing and BCR profiling (scRNA-seq/scBCR-seq). Among all B cell subsets, GC B cell is the only B cell subset that does not circulate between tissues while undergoing a short-term GC reaction during affinity maturation. Thus, focusing on GC B cells allows to address ATLO-specific B cell responses versus their SLO counterparts. Moreover, expression cloning of 60 autoantibodies from GC B cells from renal lymph nodes (RLNs) of wild-type (WT) and *Apoe*^−/−^ mice and ATLOs allowed us to characterize these autoantibodies by a variety of methods to search for rare high-affinity autoreactive and pathogenically relevant autoantibodies and identify a pathogenic atherosclerosis-specific autoantigen. Autoantigen vaccination and autoantibody transfers were then used to examine the pathogenicity of both members of the pair. To search for the relevance of H2B as an autoantigen and its corresponding autoantibody, we finally conducted a clinical study to examine whether the titer of anti-H2B autoantibodies in the circulation correlates with the disease (Extended Data Fig. 1).

### B cells and PCs in ATLOs

**Fig. 1:**
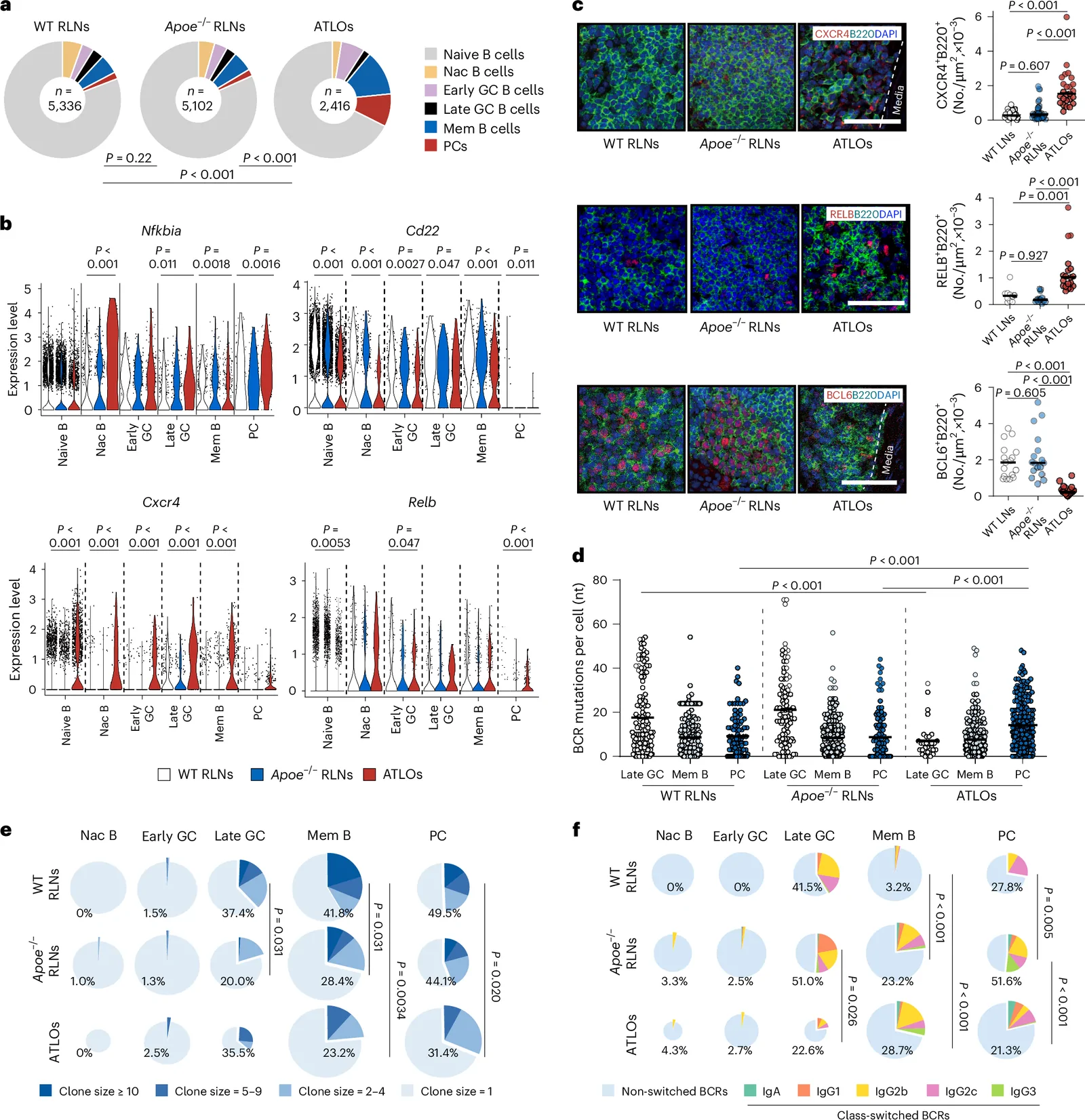
Delineation of scRNA-seq/scBCR-seq-paired B cell subsets reveals multilayered B cell dysregulation in ATLOs. **a**, Relative composition of six B cell subtypes was determined based on their transcript profiles as shown in Supplementary Fig. 1. scRNA-seq identified six B cell subsets in each tissue according to their transcript profiles: naive B cells, Nac B cells, early GC B cells, late GC B cells, Mem B cells and PCs. Percentages of each B cell subtype in each tissue are indicated by different colors in the circle plots; the total number of B cells analyzed per tissue is shown in the inner circle. Statistical analysis was performed using the *χ*^2^ test with Benjamini−Hochberg correction. **b**, Selected transcripts in individual B cell subsets. Violin plots show B cell activation (*Nfkbia*, *Relb*), migration (*Cxcr4*) and the B cell inhibitory checkpoint (*Cd22*) gene expression profiles in B cell subsets in different tissues. Each dot represents one single B cell. Statistical analysis was performed using the Kruskal−Wallis *H*-test with Benjamini−Hochberg correction. **c**, Quantitative immunofluorescent analyses of key regulatory molecule-expressing cells in WT and *Apoe*^−/−^ RLNs and ATLOs; immunofluorescence followed by morphometry of CXCR4^+^ B cells and RelB^+^ B cells but lower numbers of BCL6^+^ B cells in B cell areas. Tissue sections were stained using anti-CXCR4, anti-RelB, anti-BCL6 and anti-B220 antibodies. Quantification of the number of positive cells per area per section of CXCR4^+^ B cells (migratory B cells), RelB^+^ B cells (activated B cells) and BCL6^+^ B cells (follicular B cells) within B220^+^ B cell areas was determined as described in the Methods. Bars, 100 μm. Each dot represents one microscopy field; 10−31 random fields from 3−5 mice in each group were used for analysis. Statistical analyses were performed using one-way ANOVA with Tukey’s multiple comparison test. A two-sided *P* < 0.05 was considered significant. **d**, Number of SHMs per BCR heavy chain. Each dot represents one B cell. *P* values for pairwise comparisons were determined using a two-sided Wilcoxon rank-sum test, followed by Benjamini−Hochberg correction for multiple testing. **e**, Pie charts show distinct ATLO traits of clonally expanded versus non-expanded B cell subtypes. Sizes of circles represent the relative cell numbers within each tissue; blue indicates the percentage of clonally expanded B cells within each subset. Clonal expanded BCRs that have a clone size of 2−4, 5−9 and ≥10 are labeled with different blue colors. Statistical analysis was performed using the *χ*^2^ test, followed by Benjamini−Hochberg correction for multiple testing. **f**, Percentages of class-switched BCRs in different B cell subsets of WT and *Apoe*^−/−^ RLNs versus ATLOs. Sizes of circles represent the relative cell numbers within each tissue; blue indicates the percentages of class-switched B cell subtypes; differently switched isotypes are labeled with different colors. **e**−**f**, *χ*^2^ test with Benjamini−Hochberg correction was used to perform statistical analyses. RLN, Renal lymph node; nt, nucleotides. Source data

**Extended Data Fig. 2:**
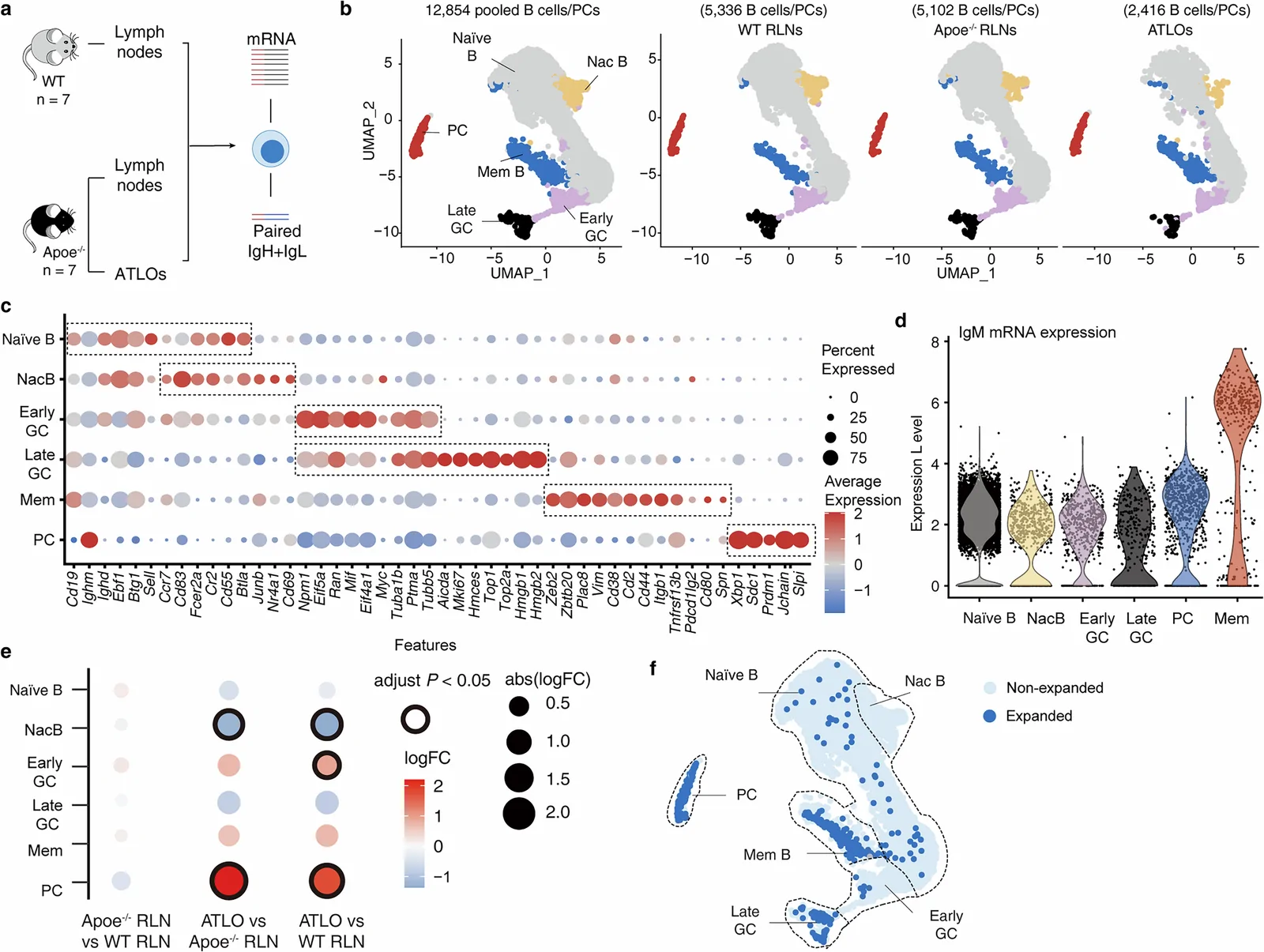
ScRNA-seq/scBCR-seq pairing reveals distinct B cell landscapes in ATLOs. (**a**) Pairing of scRNA-seq/scBCR-seq approach to compare B cell subtype transcriptomes of RLN B cell subsets of both genotypes and ATLOs. (**b**) 12,854 B cells were grouped into six subsets resulting from UMAP analyses based on their top 2000 most highly expressed genes; B cell subset distribution in the UMAP plot of WT RLNs, Apoe^−/−^ RLNs and ATLOs as naїve B cells, newly activated B cells, early GC B cells, late GC B cells, Mem B cells and PCs. (**c**) Dotplot visualization of transcript profiles of B cell subtypes; dotplots show marker signature genes to define six B cell subsets: *IgM*, *IgD*, *Sell* (selectin L) and early B cell transcription factor 1 (*Ebf1*) for naїve B cells; *Cd69* for activation; eukaryotic translation initiation factor 5a (*Eif5a*) and myelocytomatosis oncogene (*Myc*) for early GC B cells; activation-induced cytidine deaminase (*Aicda*) and proliferation-related Ki67 antigen (*Mki67*) for late GC B cells; CD38 and zinc finger E-box-binding homeobox 2 (*Zeb2*) and zinc finger and BTB domain containing 20 (*Zbtb20)* for Mem B cells; and *Sdc1* (CD138) and X-box binding protein 1 (*Xbp1*) for PCs. 6 major B cell/PC subsets were identified across SLOs and ATLOs according to their prototypical markers, that is i. naїve B cells (*IgM*^+^, *IgD*^+^, *Sell* (Selectin L)^+^, *Ebf1*^+^); ii. newly activated B cells (Nac B cells, *IgM*^+^, *IgD*^+^, *Sell*^+^, *Cd69*^+^); iii. early GC B cells (*Eif5a*^+^, *Myc*^+^, *Aicda*^−^, *Mki67*^−^); iv. late GC B cells (*Eif5a*^+^, *Aicda*^+^, *Mki67*^+^); v. Mem B cells (*Zeb2*^+^, *Zbtb20*^+^); and vi. PCs (*Xbp1*^+^, *Sdc1*^+^). (**d**) Violin plots show IgM transcripts expression by B cells and plasma cells. B cells and plasma cells were obtained from WT RLNs, Apoe^−/−^ RLNs, and ATLOs. Each dot represents one cell. (**e**) Differential abundance testing of B cell subsets was performed using edgeR to assess statistically significant changes in cellular composition across tissues. Pairwise comparisons were conducted between two groups, that is WT RLNs vs Apoe^−/−^ RLNs, ATLOs vs Apoe^−/−^ RLNs, and ATLOs vs WT RLNs. Dot plots display log10-transformed fold changes (logFC) in the relative abundance of indicated B cell subsets for each comparison. Dot color represents the direction and magnitude of logFC (red color represents increased changes, blue color represents decreased changes). Dot size corresponds to the absolute value of logFC arranging from 0.5 to 2.0 folds changes. Differences in cell abundance between groups were assessed using a quasi-likelihood F-test, and P values were adjusted for multiple testing using the Benjamini–Hochberg method. Black circle indicates statistically significant differences (adjusted P < 0.05). (**f**) UMAP visualization of clonally expanded B cell subsets; distribution of clonally expanded B cells. Clonally expanded B cells in red, not clonally expanded B cells in grey. B cells with identical IgH CDR3 amino acid sequences are considered as clonally expanded.

CD45^+^ leukocytes were FACS-sorted from RLNs of aged WT and *Apoe*^−/−^ mice and ATLOs from the same aged *Apoe*^−/−^ mice. After quality controls, 12,854 B cell transcriptomes, with matched scRNA-seq and scBCR-seq data, were retrieved. Uniform manifold approximation and projection (UMAP) algorithms identified six B cell subsets (Extended Data Fig. 2a−d): naive B cells (antigen-inexperienced); newly activated B cells (Nac B cells); early GC B cells (non-dividing antigen-experienced GC B cells); late GC B cells (antigen-experienced GC B cells undergoing proliferation, somatic hypermutation (SHM) and affinity-driven selection); memory B cells (Mem B cells); and PCs (actively secreting antibodies) (Extended Data Fig. 2c−f). ATLOs harbored all prototypical B cell subsets of RLNs but showed significantly higher percentages of early GC B cells, Mem B cells and PC subsets versus both WT RLNs and *Apoe*^−/−^ RLNs (Fig. 1a and Extended Data Fig. 2b,e).

**Extended Data Fig. 3:**
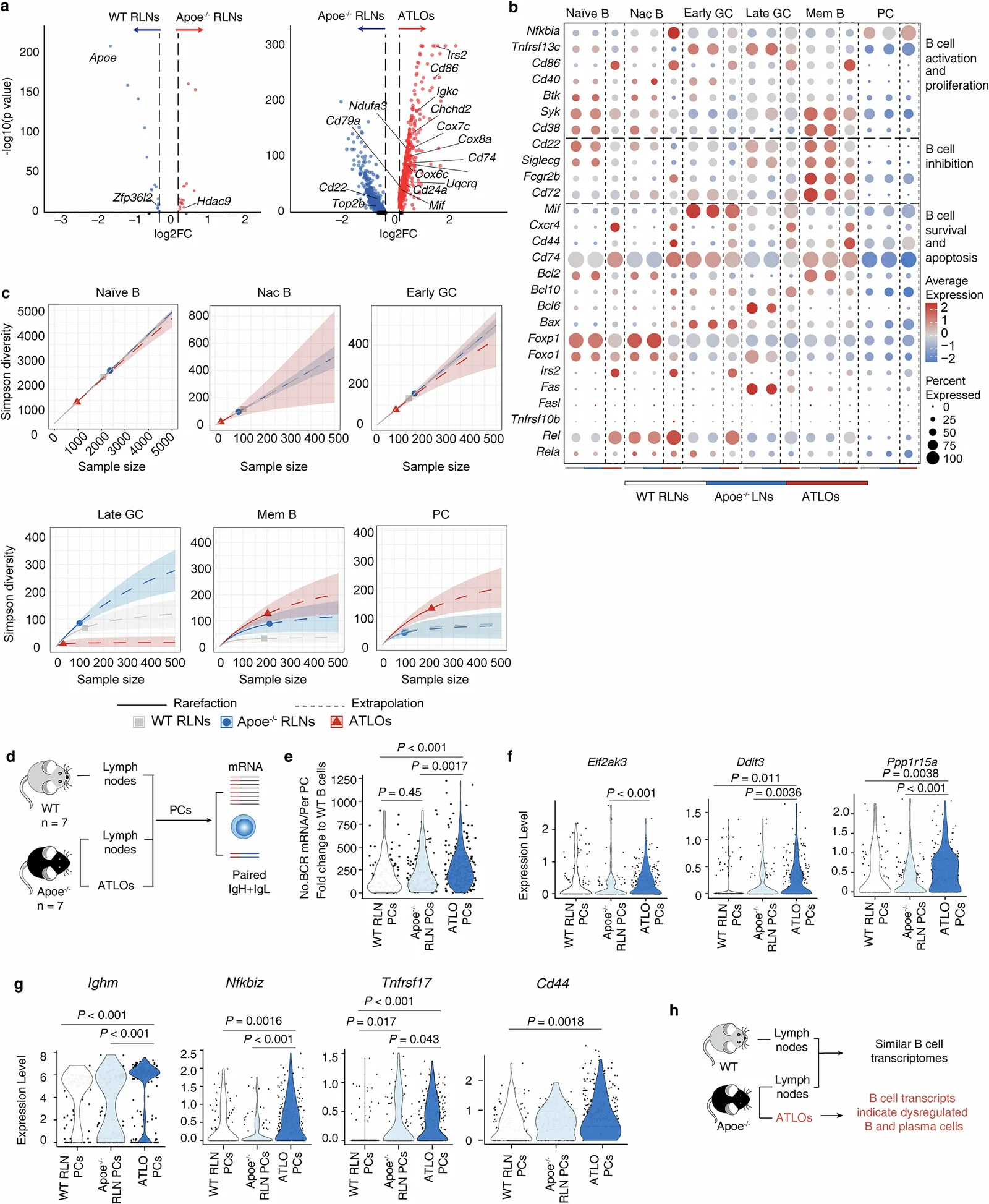
ScRNA-seq/scBCR-seq pairing reveals B cell immune response dysregulation in ATLOs. (**a**) Volcano plots show differentially expressed genes (DEGs) between two tissues. Apoe^−/−^ RLN B cells *vs* WT RLN B cells, and ATLO B cells *vs* Apoe^−/−^ RLN B cells were compared. Genes with |Log2(fold change)|>0.25 and adjusted P value < 0.05 were considered as DEGs. The adjusted P value was determined by two-sided Wilcoxon-Rank Sum test with Bonferroni correction. (**b**) Dotplots showed abnormal gene expression signatures in ATLO B cell subsets; gene expression profiles of 6 B cell subsets were determined as described in Methods. (**c**) Metrics for measuring BCR Simpson diversity. BCR diversity estimation assays show BCR repertoires in different B cell subsets. Solid (rarefaction) and dashed (extrapolation) lines mark interpolated and extrapolated regions of rarefaction curves, respectively. Simpson diversity were performed for indicated B cell subsets in WT RLNs (gray), Apoe^⁻/⁻^ RLNs (blue), and ATLOs (red). Shaded areas indicate 95% confidence intervals. Naïve B cell represents BCRs with high diversity and low expansion, PC represents BCR with low diversity and high expansion. (**d**) Pairing of scRNA-seq/scBCR-seq compares PC transcriptomes of RLNs of both genotypes versus ATLOs. (**e**) Number of immunoglobulin heavy chain (IgH) mRNAs expression per PC; scRNA-seq/BCR-sec pairing allows to quantify IgH transcripts per PC. P values for pairwise comparisons were determined using a two-sided Wilcoxon rank-sum test, followed by Benjamini–Hochberg correction for multiple testing. (**f-g**) ATLO PC transcripts involved in key protein secretion-regulating genes and function-related genes in Apoe^−/−^ RLNs vs ATLOs; Violin plots show PC protein secretion-regulating gene profiles and function-related gene expression in different tissues. P values for pairwise comparisons were determined using a two-sided Wilcoxon rank-sum test, followed by Benjamini–Hochberg correction for multiple testing. (**h**) B cell transcripts indicate B cell dysfunction in ATLOs. Source data

Differentially expressed gene (DEG) profiles in B cells revealed significant differences between ATLOs versus *Apoe*^−/−^ RLNs, but they were similar in WT RLNs versus *Apoe*^−/−^ RLNs (Extended Data Fig. 3a). ATLO B cell subsets included naive B cells, Nac B cells, early GC B cells, late GC B cells and Mem B cells. They expressed higher levels of BCR activation-related, T helper T cell-related and survival-related transcripts (*Nfkbia*, *Relb*, *Cxcr4*, *Cd86*) and lower levels of inhibitory-related and apoptosis-related transcripts (*Cd22*, *Fas*) versus their counterparts in either WT RLNs or *Apoe*^−/−^ RLNs (Fig. 1b and Extended Data Fig. 3b). The high levels of CXCR4 and RELB proteins together with lower levels of BCL6 protein expressed by ATLO B cells were confirmed and quantified at the protein level (Fig. 1c).

Because GC B cells, Mem B cells and PCs are dynamically connected, we determined the average SHM of GC B cells, Mem B cells and PCs in ATLOs and compared them with those of WT RLNs or *Apoe*^−/−^ RLNs (Fig. 1d). Although ATLO late GC B cells show lower SHM than WT RLN late GC B cells and *Apoe*^−/−^ RLN late GC B cells, ATLO PCs show significantly higher SHM than those in WT RLN PCs and *Apoe*^−/−^ RLN PCs (Fig. 1d). We defined expanded B cell clonotypes as two or more B cells with identical paired IgH and IgL CDR3 sequences. Overall, ATLO B cell subsets showed similar BCR clonal expansion when compared to their counterparts in *Apoe*^−/−^ RLNs (Fig. 1e), except for ATLO late GC B cells that display increased clonal dominance compared to *Apoe*^−/−^ RLNs, as evidenced by reduced diversity (indicator for higher BCR clonal expansion) (Extended Data Fig. 3c). Highly mutated BCRs in some PCs may mainly be derived from GC responses^40^; the significant higher number of BCR mutations in ATLO PCs does not support ATLO GC deficiency of SHM mechanisms. These data are consistent with an accelerated GC B cell−PC differentiation pathway in ATLOs.

Although late GCs were enriched by class-switched GC B cells, the class-switching recombination (CSR) event occurs during T−B cell−cell interactions and SHM events in newly activated B cells and early GC B cells^41^. In line with this model, class-switched B cells were already detected in Nac B cells and early GC B cells in *Apoe*^−/−^ RLNs and ATLOs (Fig. 1f). Of note, the class-switched BCRs in late GC B cells and PCs are significantly lower versus their counterparts in *Apoe*^−/−^ RLNs, and they remained unchanged in early GC B cells (Fig. 1f). This is consistent with CSR dysfunction during early GC to late GC reactions in ATLOs. Moreover, ATLO PCs expressed higher Ig mRNA transcripts per cell versus PCs in WT and *Apoe*^−/−^ SLOs (Extended Data Fig. 3d,e). Consistent with the markedly higher rate of Ig mRNA expression, ATLO PCs expressed significantly higher levels of unfolded protein response-related transcripts (*Eif2ak3*, *Ddit3*, *Ppp1r15a*) versus their counterparts in RLNs of both genotypes (Extended Data Fig. 3f). ATLO PCs also expressed higher levels of transcripts related to Ig secretion (*Ighm*), activation (*Nfkbiz*, *Cd44*) and survival (*Tnfrsf17*) (Extended Data Fig. 3g). These data indicated altered control of B cell/PC homeostasis in ATLOs (Extended Data Fig. 3h).

**Extended Data Fig. 4:**
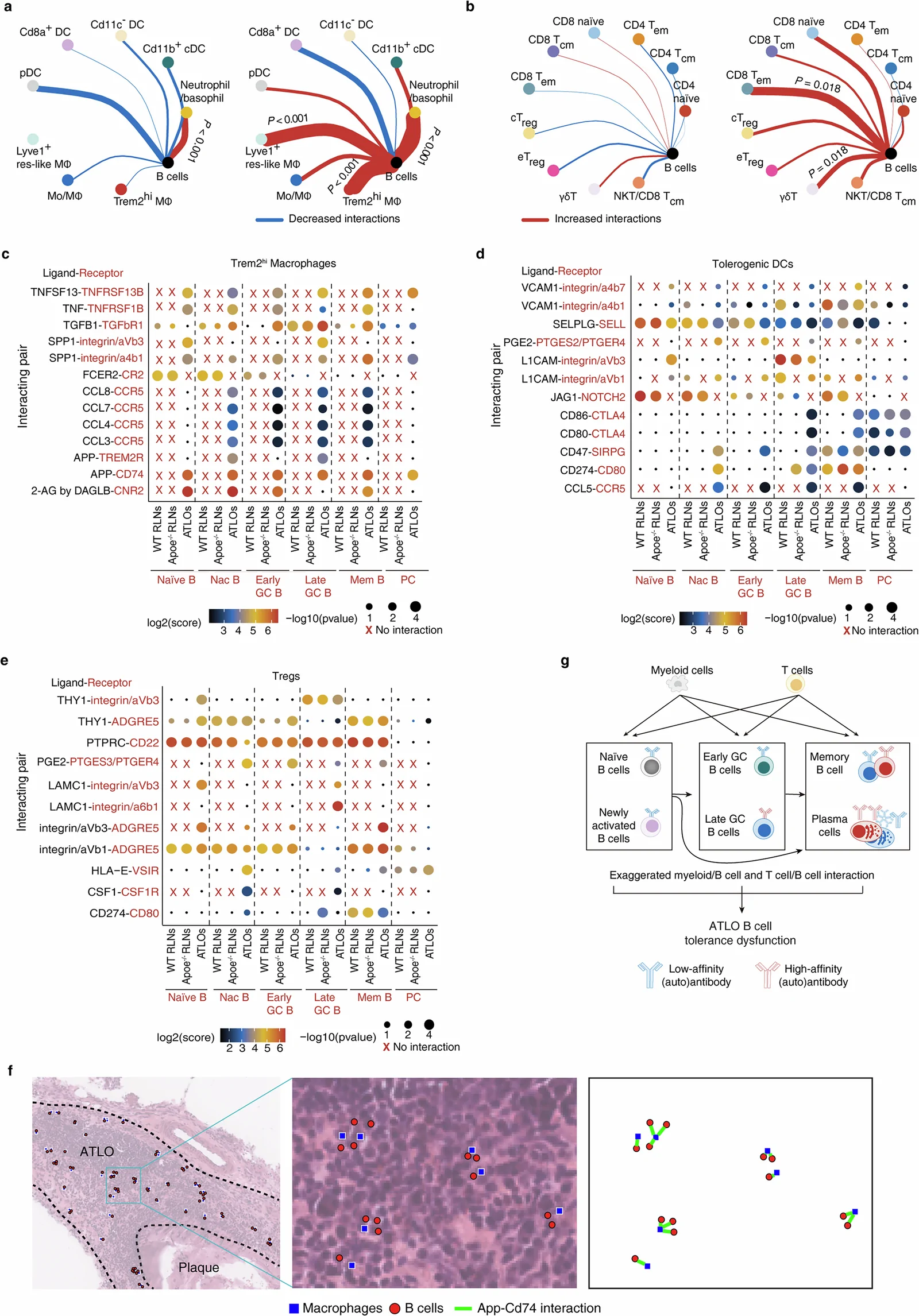
Modeling of cell-cell interactions suggests aberrant regulation of ATLO B cell immunity. (**a**) Aberrant interactions between myeloid-cell subtypes and B cells. Cell-cell interactions between myeloid-cell subsets and B cells were modeled as described in Methods. The left circus plot shows modeling of the potential number of different interactions between Apoe^−/−^ RLNs when compared to WT RLNs, the right circus plot shows modeling the numbers of potential interactions between ATLOs when compared to WT RLNs. Differences were performed by using Chi-square test with Benjamini-Hochberg correction. (**b**) Aberrant interactions between T cell subsets and B cells. Interactions between T cell subsets and B cells were modeled as described in Methods. Differences were performed by using Chi-square test with Benjamini-Hochberg correction. (**c-e**) Aberrant interaction maps in different tissues. Predicted interactions between Trem2^high^ macrophages (c), tolerogenic DCs (d), and T_reg_ cells (d) with naïve B cells, Nac B cells, early GC B cells, late GC B cells, Mem B cells, and PCs in WT RLNs, Apoe^−/−^ RLNs, and ATLOs. Color scale indicates normalized expression, dot size indicates significance (-log10 p value), x represents no predicted interactions was detected. Statistical significance of ligand–receptor interactions was assessed using a one-sided permutation test implemented in CellPhoneDB. (**f**) Validation of APP-CD74 interaction pairs by macrophages and B cells in ATLOs of aged Apoe^−/−^ mice. Left panel: Spatial distribution of the APP-CD74 interaction pair between macrophages and B cells within ATLO. Middle panel: Representative high-magnification view of a selected ATLO region showing the APP-CD74 interaction pair at single-cell level. Right panel: Schematic figure illustrating the interaction mode in the zoomed-in region shown in the middle panel. (**g**) Aberrant myeloid-cell and T cell interactions with B cell subsets indicate dysregulation of major immune checkpoints in ATLOs. Red arrows indicate aberrant interactions between myeloid cell and T cell interactions with B cell subsets.

We next modeled T−B and myeloid cell−B cell interaction networks within ATLOs versus those of WT and *Apoe*^−/−^ RLNs (Extended Data Fig. 4a,b). ATLO B cells showed enhanced interactions with Lyve1^+^ resident-like macrophages, Trem2^high^ macrophages, CD8^+^ effector memory T cells and γδ T cells versus their counterparts in RLNs (Extended Data Fig. 4a,b). ATLO B cell transcriptomes were enriched in B cell survival, migration and tolerance-regulating genes including *Tnfrsf13b* (encoding transmembrane activator and calcium modulator and cyclophilin ligand interactor, a B cell survival factor), *Ccr5* (involved in immune cell migration), integrins (controlling B cell trafficking), *Cd86* (associated with breakdown of self-tolerance^42^) and *Cd80* (promoting B cell activation) (Extended Data Fig. 4c−e). By spatial transcriptomic analyses, we observed that key interactions may occur, including the APP−CD74 interaction pair by macrophages and B cells in ATLOs (Extended Data Fig. 4f). These data indicated that enhanced T−B and myeloid−B interactions during the pre-GC, GC and post-GC compartments of ATLOs may contribute to B cell immune responses specifically in ATLOs (Extended Data Fig. 4g).

### Dysregulated GC B cells in ATLOs

**Fig. 2:**
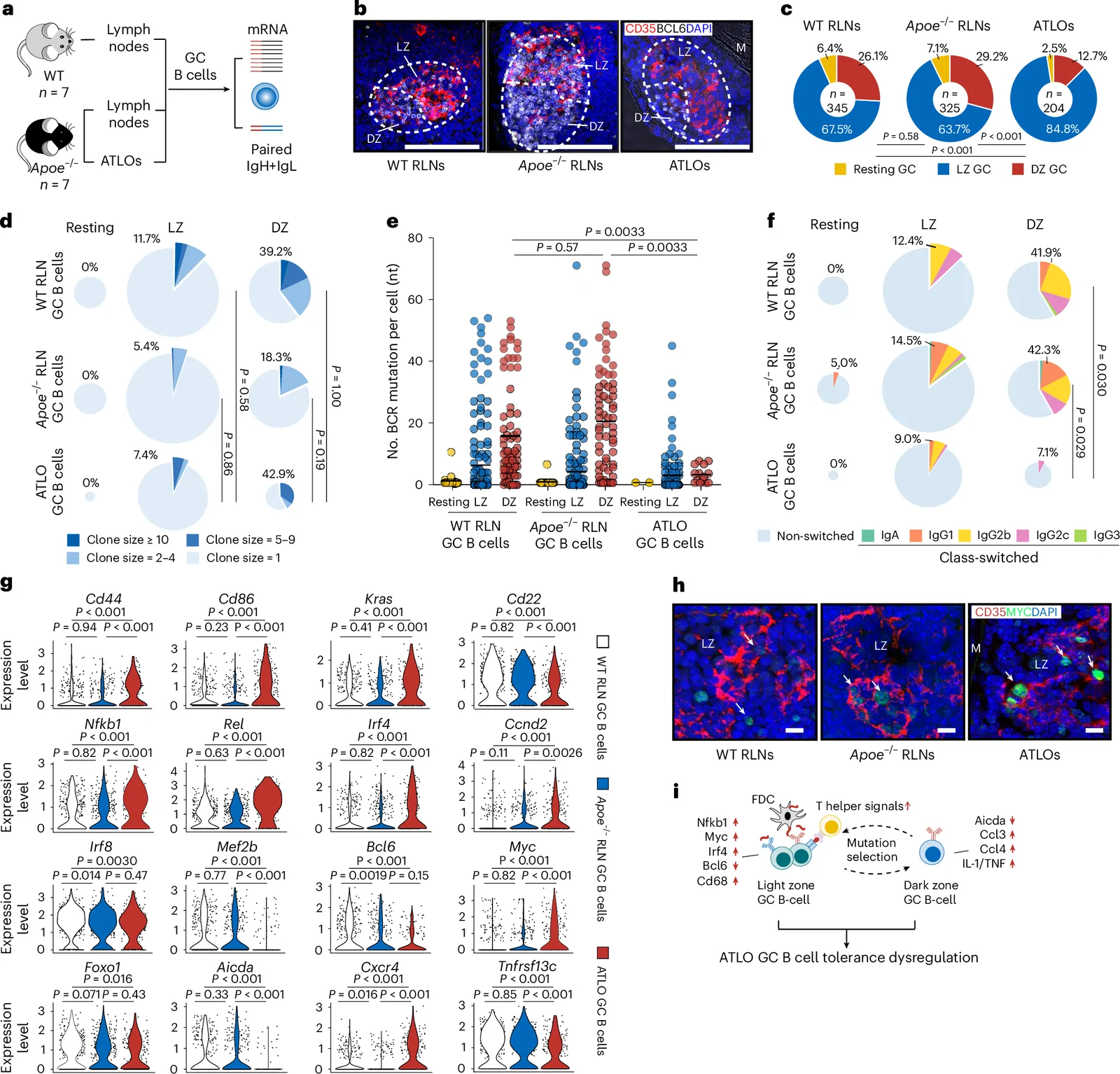
ATLO GCs reveal altered DZ−LZ structures, and their B cell transcript signatures are dysregulated. **a**, GC responses using pairing of scRNA-seq with scBCR-seq; pairing of scRNA-seq/scBCR-seq was performed as described in the Methods. **b**, Stage III ATLOs in activated B cell follicles accommodate separate LZs and DZs in GCs. Adventitia segments adjacent of heavy atherosclerotic plaques were selected to stain FDC networks with anti-CD35 antibodies for LZs, and B cell areas outside CD35^+^ networks were stained with anti-BCL6 antibodies for DZs. Data are representative of at least three independent experiments. **c**, ATLOs reveal aberrant GC B cell composition; percentages of resting GC B cells, LZ GC B cells and DZ GC B cells in WT RLNs, *Apoe*^−/−^ RLNs and ATLOs as described in the Methods. The total number of GC B cells analyzed in each tissue is shown in the inner circle. *χ*^2^ test was used to compare the GC B cell subset compositions, with *P* values adjusted for multiple testing using the Benjamini−Hochberg method. **d**, ATLOs maintain expanded versus non-expanded B cell compositions similar to WT GCs; percentages of clonally expanded versus non-expanded B cells in different GC B cell subsets in WT and *Apoe*^−/−^ RLNs and ATLOs. *χ*^2^ post hoc test with Benjamini−Hochberg correction. Clonal expanded BCRs that have a clone size of 2−4, 5−9 and ≥10 are labeled with different blue colors. **e**, Quantification of SHM per antibody sequence imaged as violin plots in ATLOs shows lower levels of BCR mutations per GC B cell; each dot represents one B cell. Pairwise comparisons among groups were performed using the Wilcoxon rank-sum test, with *P* values adjusted for multiple testing using the Benjamini−Hochberg method. **f**, ATLOs reveal distorted CSR rates; percentages of CSR rates in different GC B cell subsets in different tissues. *χ*^2^ test with Benjamini−Hochberg correction was used to perform statistical analysis. Different switched isotypes are labeled with different colors. **g**, Function-associated GC B cell gene expression profiles reveal exaggerated patterns. Violin plots show representative gene expression levels associated with GC B cell features: B cell activation (*Cd44*, *Cd86*), inhibitor of B cell activation (*Cd22*), BCR activation (*Kras*, *Ccnd2*), activation of co-stimulation (*Nfkb1*, *Rel*), master transcription regulators (*Irf8*, *Mef2b*, *Myc*, *Bcl6*), SHM signals (*Foxo1*, *Aicda*), B cell migration (*Cxcr4*) and survival signals (*Tnfrsf13c*/BAFFR). Each dot represents one B cell. Statistical analysis was performed using the Kruskal−Wallis *H*-test with Benjamini−Hochberg correction. **h**, Higher numbers of MYC^+^ GC B cells in ATLOs; anti-CD35 antibodies and anti-MYC antibodies were used to stain WT RLN, *Apoe*^−/−^ RLN and ATLO sections of aged WT and *Apoe*^−/−^ mice. Representative images are shown from 3−4 mice per group. **i**, Schematic of aberrant features indicates ATLO GC B cell dysregulation. nt, nucleotides. Source data

GCs arise in activated B cell follicles of lymphoid organs to organize B cell clonal expansion, SHM and BCR affinity maturation. Powerful tolerance mechanisms in GCs eliminate autoreactive high-affinity pathogenic antibodies^34,36,40^. TLO GCs have been reported to contain FDCs in activated B cell follicles in multiple disease conditions^23,24,40^. We observed considerable heterogeneity within ATLOs as defined by scattered T−B clusters (stage I) to advanced stages with CD35^+^ FDCs ranging from small patches of FDCs to larger GCs with well-separated light zones (LZs, BCL6^+^CD35^+^) and dark zones (DZs, BCL6^+^CD35^−^) (stage III) (Fig. 2a,b). ATLO GCs were exclusively observed in *Apoe*^−/−^ mice with heavy atherosclerosis burdens^29^ (Fig. 2b). These data indicated that ATLOs contain a range of immature (without GCs) to mature (with GCs) stages, reminiscent of those found in cancers^43^.

**Extended Data Fig. 5:**
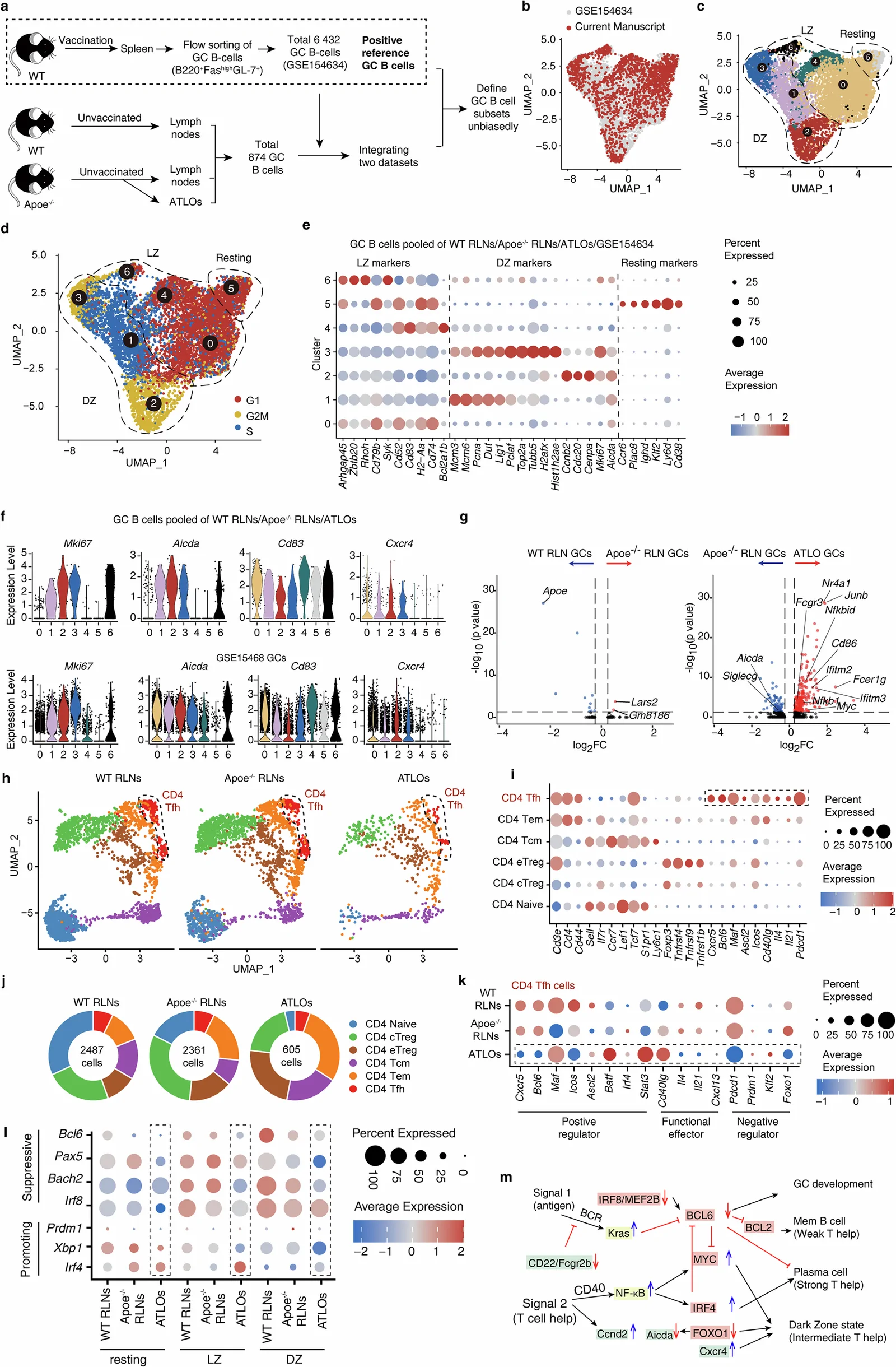
B cell transcript signatures are dysregulated in ATLO GCs. (**a**) Integration of published B-cell subsets of vaccinated WT mouse spleens (GSE154634) into B-cell subsets in different tissues and ATLOs. (**b**) Integration of GC B cells from WT RLNs, Apoe^−/−^ RLNs, and ATLOs with GC B cells in spleens of vaccinated WT mice; GC B cells of vaccinated WT mice (obtained from a publicly available databank: GSE154634) were integrated into our own GC B cells from unvaccinated WT GC B cells isolated from RLNs or Apoe^−/−^ RLNs or ATLOs. B cells from our own data are colored, cells from their spleen counterparts of vaccinated mice (GSE154634) were labeled in grey. (**c**) Identification of 7 GC B cell subtypes in all pooled GC B cells. All GC B cells are grouped into 7 subtypes following in tSNE map analyses based on their top 2000 highly expressed genes; 7 GC B cell subtypes are further grouped into light zone (LZ) GC B cells (clusters 0, 4, 6), dark zone (DZ) GC B cells (clusters 1, 2, 3), and resting GC B cells (cluster 5). (**d**) TSNE map shows cell cycle markers (G1, G2M, and S phase) of GC B cells from pools of GC B cells. (**e**) Prototypic marker genes to profile LZ, DZ and resting GC B cells in pooled GC B cells; dot plot shows expression of GC B cell-related markers in 7 GC subtypes obtained from vaccinated WT mice plus GC B cells of WT RLNs, Apoe^−/−^ RLNs, and ATLOs. (**f**) Prototypic marker genes expressed by GC B cells obtained from pooled of WT RLNs, Apoe^−/−^ RLNs, and ATLOs (upper panel). Prototypic marker genes expressed by spleen GC B cells from GSE154634 were showed in lower panel. Violin plots show representative gene expression levels associated with GC B cell subsets including *Mki67* (proliferation), *Aicda* (enzyme mediates BCR mutation and class switching), *Cd83* (activation) and *Cxcr4* (migration) in WT RLNs, Apoe^−/−^ RLNs, and ATLOs; Each dot represents one single B cell. (**g**) DEGs in GC B cells in WT RLN/Apoe^−/−^ and Apoe^−/−^ RLN/ATLO comparisons; Volcano plot of DEGs of WT RLN GCs, Apoe^−/−^ RLN GCs, ATLO GCs. Genes with |Log2(fold change)|>0.25 and adjusted P value < 0.05 were considered as DEGs. The adjusted P value was determined by two-sided Wilcoxon-Rank Sum test with Bonferroni correction. (**h**) UMAP visualizes the CD4 T cell subsets among different tissues in aged WT and Apoe^−/−^ mice. (**i**) Dotplot displays the highly expressed genes in different CD4 T cell subsets. (**j**) Proportion of CD4 T cell subsets in three different tissues. (**k**) Expression levels of Tfh phenotype and functional associated genes among different tissues. (**l**) Key regulatory genes involved in the plasma cell differentiation pathway in different tissues. Dot plots visualize the expression level of genes promoting or suppressing plasma cell differentiation among GC subsets across different tissues. (**m**) The transcriptional regulation network was constructed based on current understanding of GC B cell biology in SLOs. ATLO GC B cells showed both upregulation (blue arrows) and downregulation (red arrows) of genes in the regulation networks. The expressions of BCL6, CXCR4, RelB were confirmed at protein levels. Source data

We next explored ATLO GC B cells versus WT and *Apoe*^−/−^ RLN GCs at single-cell resolution. To define GC B cell subsets in scRNA-seq data, we integrated 874 GC B cells (WT RLNs, *Apoe*^−/−^ RLNs and ATLOs) into a well-established GC B cell pool as reported previously (GC B cells obtained from WT mice vaccinated with 4-hydroxy-3-nitrophenylacetyl or ovalbumin; GSE154634 (ref. ^44^)) (Extended Data Fig. 5a). This strategy allowed us to evaluate the relatively small number of GC B cells (874 GC B cells) representing the immobile bona fide GC B cells. Data showed that GC B cells obtained from WT RLNs, *Apoe*^−/−^ RLNs and ATLOs were also separated into DZ GC B cells (*Cxcr4*^+^, *Mki67*^+^, *Aicda*^+^, *Cdc20*^+^; proliferating cells in the S−G2/M transition of the cell cycle), LZ GC B cells (*Cd83*^+^, *Cxcr4*^low^, *Cd74*^+^) and resting GC B cells (*Ccr6*^+^, *Ighd*^+^, *Ly6d*^+^) (Extended Data Fig. 5b−f). WT and *Apoe*^−/−^ RLN GC B cells showed similar ratios of LZ to DZ GC B cells (Fig. 2c), whereas ATLO GCs were distinct in their cellular composition with reduced DZs and increased LZs (Fig. 2c).

To examine whether ATLO GC B cells may be associated with potential functional changes, we examined clonal expansion, SHM and class-switched BCRs of each GC subset. DZ GC B cells showed the highest levels of clonal expansion among all three GC B cell subsets in all three tissues (Fig. 2d), consistent with the expression of high levels of proliferation-related genes as expected. No apparent differences were observed between BCR clonal expansion among the three GC B cell subsets in WT RLNs, *Apoe*^−/−^ RLNs and ATLOs (Fig. 2d). In WT and *Apoe*^−/−^ RLNs, DZ GC B cells showed higher levels of SHM and isotype-switched BCRs when compared to LZ GC and resting GC B cells, and no apparent difference was observed between these two genotypes (Fig. 2e,f). Interestingly, DZ GC B cells (but not LZ GC B cells) in ATLOs showed the lowest levels of BCR mutations and isotype-switched BCRs versus their counterparts in WT RLNs and *Apoe*^−/−^ RLNs (Fig. 2e,f). These B cell responses indicated that ATLO GCs include a dysregulated B cell immune response environment.

To understand the underlying mechanisms of dysregulated ATLO GCs, we examined gene expression by GC B cells obtained from different tissues. By two-group comparisons, the transcripts of GC B cells showed major overlaps between *Apoe*^−/−^ GC B cells versus WT GC B cells (Extended Data Fig. 5f,g). However, ATLO GC B cells selectively upregulated B cell activation-related (Fc receptors, NF-κB-related), interferon-related transcripts and downregulated B cell tolerance inhibitory (*Cd22*) and SHM-related transcripts (*Aicda*) (Extended Data Fig. 5f,g). These data indicated that dysregulated immune responses selectively occurred in ATLO GCs but to a much lesser degree in *Apoe*^−/−^ lymph node GCs.

The dynamics of GC B cells, Mem B cells and PCs are controlled by a wide range of B cell pathways, including BCR and co-stimulatory signaling and T follicular helper (T_FH_) cell signaling by GC B cells^34,36,40^. We next examined these transcripts expressed by GC B cells by WT RLNs, *Apoe*^−/−^ RLNs and ATLOs. We noticed significant changes of transcript repertoires in multiple B cell-regulating pathways. ATLO GC B cells expressed higher levels of B cell activation transcripts (*Cd44*, *Cd86*), BCR activation (*Kras*, *Ccnd2*), activation of co-stimulation (*Nfkb1*, *Rel*), master transcription regulators (*Irf4*, *Myc*) and B cell migration (*Cxcr4*^high^) related transcripts, and they expressed lower levels inhibitor of B cell activation (*Cd22*), master transcription regulators (*Bcl6*), positive regulation of Bcl6 (*Irf8*, *Mef2b*), BCR mutation and class switching (*Foxo1*, *Aicda*) and B cell survival (*Tnfrsf13c*/BAFFR) (Fig. 2g). Myc expression by LZ cells is one of several regulatory steps for T_FH_-dependent positive selection^45^. We confirmed the presence of Myc^+^ LZ cells in RLN LZs and in ATLO LZs (Fig. 2h), supporting the capacity of positive selection in GC B cells in RLNs and ATLOs.

T_FH_ cells contribute to the differentiation of GC B cells to PCs in SLOs. We further examined the presence of T_FH_ cells (*Cxcr5*, *Pdcd1*, *Cd40lg*, *Icos* and *Bcl6*) in RLNs and ATLOs (Extended Data Fig. 5h–j). Our data showed that ATLO T_FH_ cells expressed higher levels of transcripts related to its activation (that is, *Cd40l*) and inhibitory (that is, *Pdcdl1*) functions (Extended Data Fig. 5k). These data indicated that ATLO GC B cells receive strong T helper signals in ATLO GC reactions. We also identified gene transcripts contributing to GC B cell differentiation into PCs^46,47^. ATLO GC B cells expressed higher expression of genes promoting differentiation of GC B cells to PCs (for example, *Irf4*, *Prdm1*, *Xbp1*) and lower expression of gene transcripts suppressing this process (for example, *Pax5*, *Bach2*, *Irf8*, *Bcl6*) (Extended Data Fig. 5l).

The transcript data indicate that ATLO GC B cells receive strong signals from antigen stimulation (signal 1) and T cell helper (signal 2), which indicates an accelerated GC B cell−PC differentiation pathway in ATLOs (Extended Data Fig. 5m).

**Extended Data Fig. 6:**
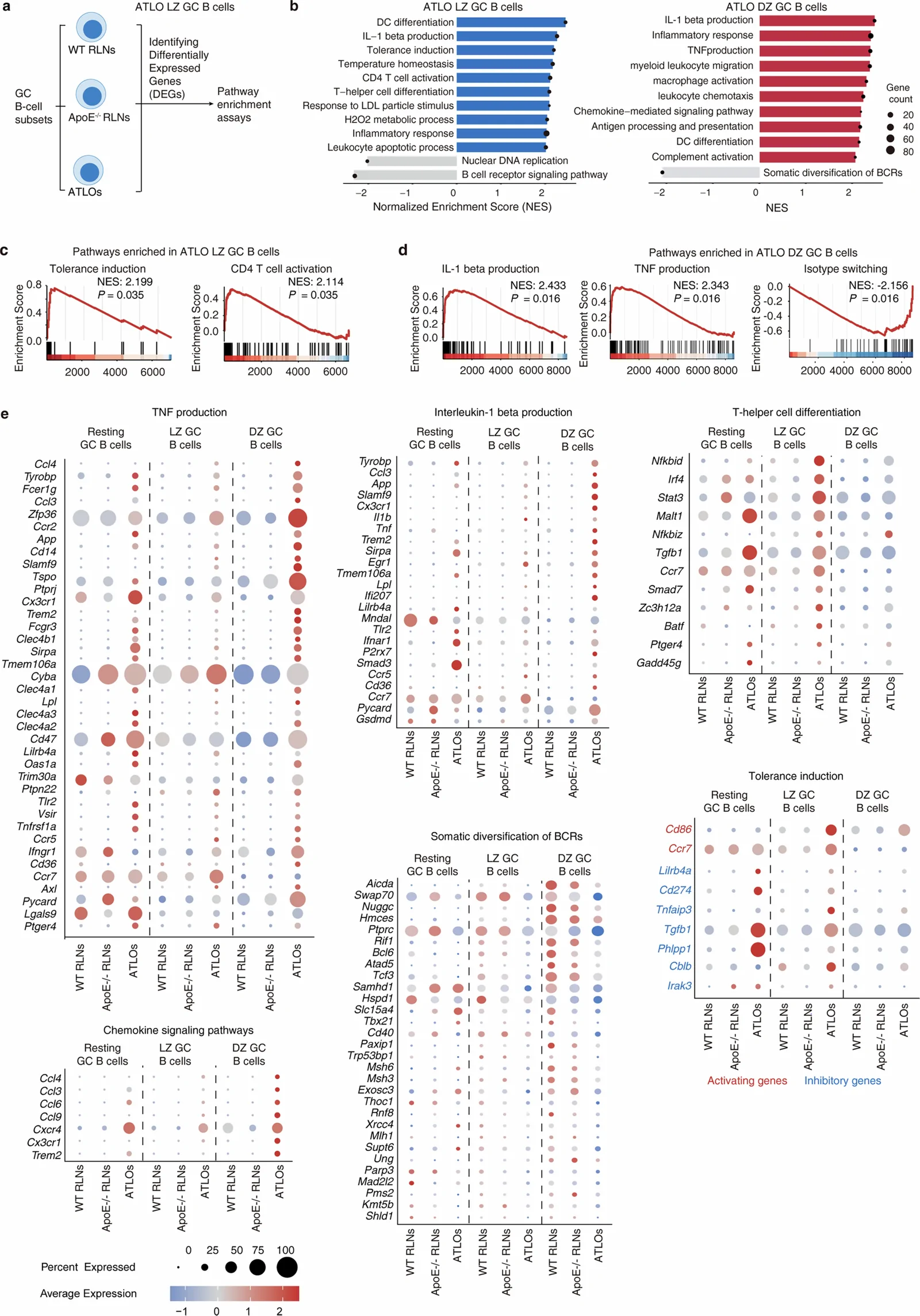
ATLO GC B cells show increases in GC B cell-associated cytokine pathways and defects of B cell maintenance. (**a**) Schematic of experimental approach to identify differentially regulated genes in SLO vs ATLO GC B cell subsets; identification of DEGs of GC B cells was followed by gene set enrichment analyses (GSEA). (**b**) Normalized enrichment scores (NES) of GO terms in ATLO GC B cells vs their counterparts in WT RLNs. (**c-d**) GSEA assays of selected GO terms enriched in ATLO LZ GC B cells and ATLO GC B cells vs WT and Apoe^−/−^ RLNs. The x-axis shows genes (vertical black lines) represented in the GO terms and the color indicated positive (red) or negative (blue) correlation. The green line connects gene and enrichment score of selected biological pathways. Enrichment significance was assessed using a permutation-based algorithm implemented in the clusterProfiler package, and P values were adjusted for multiple testing using the Benjamini–Hochberg method. NES: normalized enrichment score. (**e**) Dotplot shows individual gene expression profiles involved in different biological functions for each GC B cell subset.

Next, we performed gene set enrichment analyses (GSEAs) of ATLO LZ GC B cells and DZ GC B cells versus their counterparts in WT RLN GCs and *Apoe*^−/−^ RLN GCs (Extended Data Fig. 6a–d). ATLO LZ GC B cells were positively enriched in genes that were involved in CD4 T cell activation, dendritic cell differentiation and tolerance regulation and negatively enriched in Gene Ontology terms in DNA replication and the BCR signaling pathway (Extended Data Fig. 6b,c). Interestingly, ATLO DZ GC B cells were positively enriched in Gene Ontology terms of TNF and IL-1β production and negatively enriched in the SHM-related Gene Ontology term (Extended Data Fig. 6b,d). Moreover, ATLO LZ GC B cells expressed high levels of T helper cell differentiation-related (*Nfkbid*, *Irf4*, *Stat3*) and tolerance breakdown-related (*Cd86*, *Ccr7*) transcripts (Extended Data Fig. 6e,f). ATLO DZ-GC B cells expressed high levels of members of the TNF family and IL-1β production-mediated and chemokine-mediated signaling pathway-related transcripts (that is, *Il1b*, *Ccl3*, *Ccl4*, *Ccl6*, *Ccl9*) (Extended Data Fig. 6e,f). These data indicated that the different B cell signals in LZ and DZ GC B cells (Fig. 2i) may determine distinct B cell fates versus their counterparts in RLNs of both genotypes and that these phenotypes may contribute to the aberrant GC structure of ATLOs.

### The GC−PC differentiation pathway in ATLOs

**Fig. 3:**
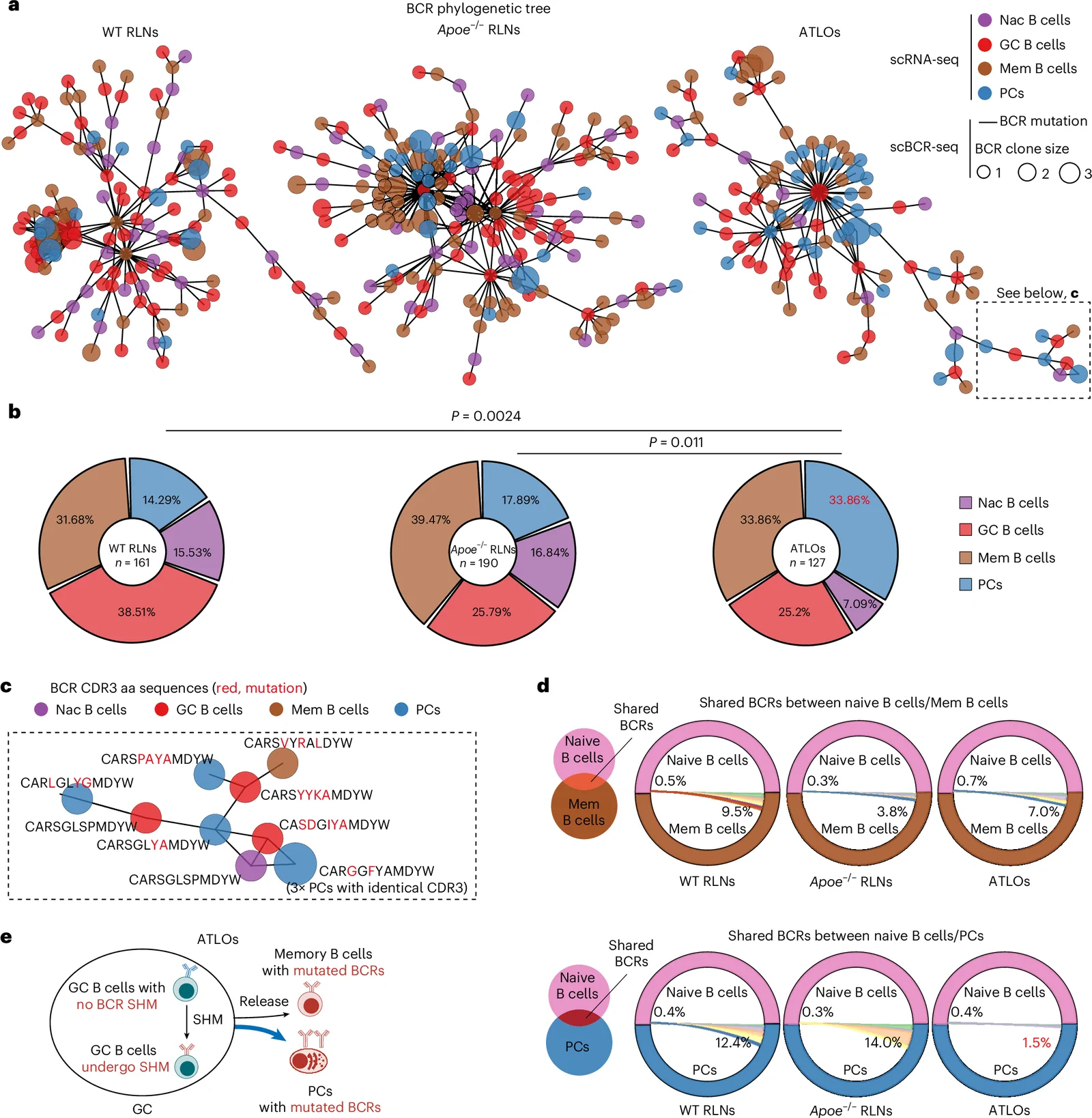
Accelerated GC B cell−PC differentiation in ATLOs. **a**,**b**, BCR phylogenetic trees reconstructed the series of subsequent clone mutations using scRNA-seq BCR pairing in different tissues. BCR phylogenetic tree analyses were performed to study the dynamics of Nac B cells, GC B cells, Mem B cells and PCs in different tissues based on scRNA-seq/scBCR-seq. The percentages of PCs within BCR phylogenetic trees were compared. Differences between groups were assessed using the *χ*^2^ test, followed by Benjamini−Hochberg correction for multiple testing. **c**, Example of how the algorism uses BCR mutation information to build the BCR phylogenetic tree in ATLOs. **d**, The shared BCRs between naive B cells/Mem B cells or shared BCR between naive B cells/PCs were examined. **e**, A hypothetical model of accelerated GC B cell−PC differentiation in ATLOs. Nac B cells, GC B cells, Mem B cells and PCs form a dynamically connected B cell reaction pathway that includes potentially different release mechanisms of the emerging Mem B cells and PCs emerging from the GC reaction. aa, amino acid. Source data

To obtain direct evidence rather than relying only on transcript repertoire-type datasets for an accelerated GC B cell−PC differentiation pathway in ATLOs, we decided to trace B cells in ATLOs. Our approach to pair scRNA-seq with scBCR-seq allows the construction of the B cell phylogenetic tree, which is a widely used approach to connect B cell subsets with BCR mutations within a clone^48^. Phylogenetic trees can link GC B cell clones, Mem B cells and PCs. We constructed BCR phylogenetic trees to study the links of GC B cell, Mem B cell and PC clones in different tissues (Fig. 3a). These data support the existence of GC B cell−PC differentiation pathway in ATLOs and RLNs (Fig. 3a). By quantification of B cell phylogenetic trees in ATLOs, WT RLNs and *Apoe*^−/−^ RLNs, our data show similar differentiation trajectories among GC B cells/Mem B cells/PCs in WT RLNs and *Apoe*^−/−^ RLNs (Fig. 3b). By contrast, the GC B cell−PC differentiation pathway is significantly enhanced in ATLOs versus their lymph node counterparts (Fig. 3b,c). The BCR phylogenetic tree data support an accelerated GC B cell−PC differentiation pathway in ATLOs.

**Fig. 4:**
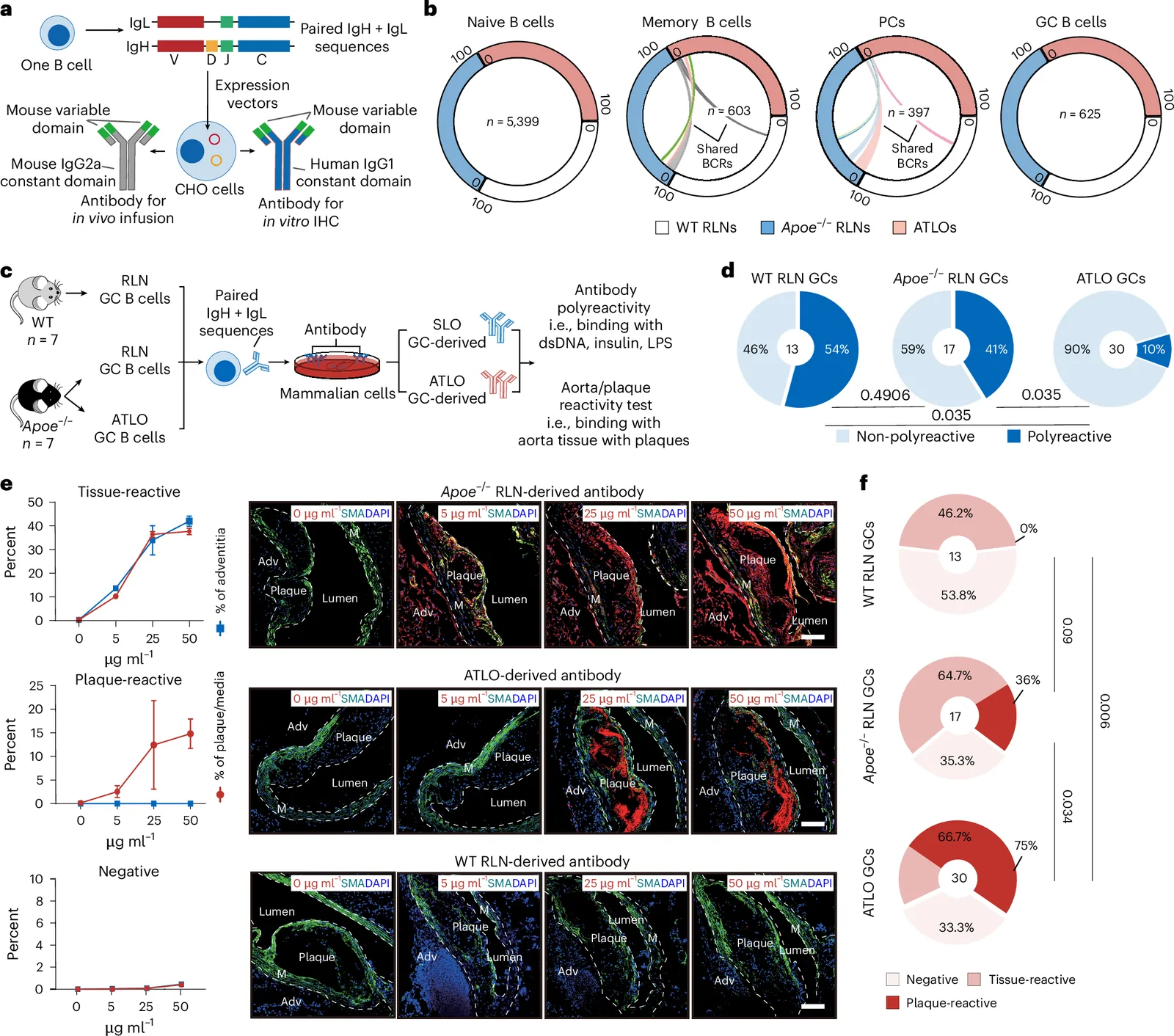
ATLO GCs generate diseased arterial wall-specific autoantibodies with high rates of plaque reactivity. **a**, Schematic diagram shows generation of recombinant mouse monoclonal antibodies. The identical mouse variable regions were used to generate mouse−human recombinant antibodies with the human IgG1 constant domain. **b**, Shared BCRs were identified between memory B cells and PCs among WT RLNs, *Apoe*^−/−^ RLNs and ATLOs. No shared BCR was observed in GC B cell compartments and naive B cells. The total number of each B cell subsets analyzed in each tissue is shown in the inner circle. **c**, Schematic view of experimental design of sequencing, cloning, expression and characterization of GC B cell-derived autoantibodies. The variable regions of paired IgH + IgL chains were sequenced, followed by cloning of the paired sequences into expression plasmids. After transfection into and expression by cultured cells, antibodies were purified from the culture supernatant. Antibodies were examined for immunoreactivities toward tissues and broad autoantigens—that is, dsDNA, insulin and LPS; antibodies were screened for reactivity toward aorta/plaque tissues by immunofluorescence staining. **d**, ATLOs selectively show markedly reduced polyreactivity; antibody polyreactivity against at least two of three structurally unrelated antigens: LPS, insulin and dsRNA were defined as polyreactive. Quantification of polyreactive antibodies in WT RLN GC-derived, *Apoe*^−/−^ RLN GC-derived and ATLO GC-derived antibodies. The number of antibodies examined is listed in the inner circle. Two-sided Fisher’s exact test. **e**, Immunofluorescence staining of three different patterns of antibody in mouse atherosclerotic plaques. Different antibody concentrations (0, 5, 25, 50 μg ml^−1^) were used to stain serial sections of arteries. Three antibody staining patterns are shown: (1) no staining (aorta-negative) of all tested antibody concentrations; (2) stains positive for atherosclerotic plaques, media, adjacent adipose and connective tissues (aorta-reactive); and (3) stains positive for plaques and media (plaque-reactive). Bars, 100 μm. Data are presented as mean ± s.e.m. Data and representative images are shown from *n* = 3 mice. **f**, Quantification of different antibody patterns in RLNs versus ATLO GC-derived antibodies; ATLO GC-derived antibodies show high plaque reactivity. The number of antibodies is listed in the inner circle. *χ*^2^ test was used to compare differences, with *P* values adjusted for multiple testing using the Benjamini−Hochberg method. Adv, adventitia; M, media; CHO, Chinese hamster ovary; IHC, immunohistochemistry. Source data

Some Mem B cells or PCs are formed without undergoing SHM, which is a well-established part of GC-independent Mem B cell and PC differentiation pathway^49^. We next examined the shared BCRs between naive B cells/Mem B cells or shared BCRs between naive B cells/PCs (Fig. 3d). These data showed that shared BCRs between naive B cells/Mem B cells exist but that they are similar in WT RLNs, *Apoe*^−/−^ RLNs and ATLOs (Fig. 3d). Of note, the number of shared BCRs between naive B cells/PCs is largely reduced in ATLOs (Fig. 3d). Together, these data are consistent with a model of an accelerated GC B cell−PC differentiation pathway in ATLOs (Fig. 4e).

### ATLO GC-derived autoantibodies skew to arterial wall and atherosclerotic plaque-reacting autoantigens

Antibodies constitute important effectors of B cells and PCs. To study the functional impact of ATLO B cell immunity, we chose to clone paired Ig heavy (IgH)/Ig light (IgL) chains from single B cells and generate the recombinant antibodies (Fig. 4a) from WT RLNs, *Apoe*^−/−^ RLNs and ATLOs. This strategy allowed us to quantitatively compare antibody characteristics originating from RLNs versus ATLOs. To ascertain that we are truly looking at B cells specifically emerging in ATLOs or their SLOs counterparts, we focused on GC B cells. GC B cells are non-migrating B cell subsets that are engaged in the process of GC reaction. In sharp contrast, Mem B cells and PCs circulate among blood, lymph nodes and spleen^34,36^. In our limited number of BCR/antibody sequences, we did not detect any shared BCRs expressed by GC B cells in different tissues, but we observed shared BCRs expressed by Mem B cells and PCs by RLNs and ATLOs (Fig. 4b).

We obtained 30 antibodies from RLNs (13 from WT RLN GCs, 17 from *Apoe*^−/−^ RLN GCs) and 30 from *Apoe*^−/−^ ATLO GCs for subsequent antibody functional assays (Fig. 4c). None of the 60 expression-cloned antibodies shared heavy or light CDR3 sequences (Supplementary Table 1). First, we determined whether ATLO GC B cells undergo affinity maturation, which is associated with reduced polyreactivity^50^. To understand polyreactivity versus oligoreactivity in atherosclerosis and in ATLOs in particular, we examined antibody binding to three structurally unrelated antigens—that is, insulin, double-stranded DNA (dsDNA) and lipopolysaccharide (LPS)—all representing self-antigens associated with low-affinity-binding antibodies as reported^50^. Fifty-four percent of WT RLN GC-derived antibodies and 41% of *Apoe*^−/−^ RLN GC-derived antibodies were polyreactive (binding at least two of the three antigens; Fig. 4d and Supplementary Table 1). By contrast, only 10% of ATLO GC-derived antibodies were polyreactive (Fig. 4d and Supplementary Table 1). Decreasing antibody polyreactivity in GC reactions is expected to be associated with increasing antigen specificity^50^. These data provide functional evidence for dysregulated ATLO GC B cell autoimmune responses.

Next, we examined the ATLO GC-derived autoantibodies and RLN GC-derived autoantibodies for binding to arterial walls, including atherosclerotic lesions. For this purpose, we stained atherosclerotic mouse aorta tissues, including plaque, media, adventitia and adjacent adipose tissue (Fig. 4e). By immunofluorescence staining, we observed that 46% (6/13) of WT RLN GC-derived antibodies, 65% (11/17) of *Apoe*^−/−^ RLN GC-derived antibodies and 67% (20/30) of ATLO GC-derived antibodies recognized the diseased aorta and adjacent adipose tissue (referred to hereafter as ‘tissue-reactive antibodies’; Fig. 4e,f and Supplementary Table 1). A subset of tissue-reactive antibodies (36%, 4/11 *Apoe*^−/−^ RLN GC B cells; 75%, 15/20 ATLO GC B cells) specifically recognized atherosclerotic plaques (‘plaque-reactive’) (Fig. 4e,f and Supplementary Table 1). By contrast, none of the tissue-reactive antibodies (0/6) obtained from WT RLN GCs was plaque-reactive (Fig. 4f). The significantly higher levels of plaque-reactive antibodies in ATLO GCs (Fig. 4f) suggests that ATLO GCs generated self-reactive B cells that were skewed toward arteries and/or plaques. These data were consistent with the possibility that ATLO GC B cells were functionally distinct when compared to their brethren in SLO GCs.

### ATLO GCs encode a high-affinity autoantibody in atherosclerosis

**Fig. 5:**
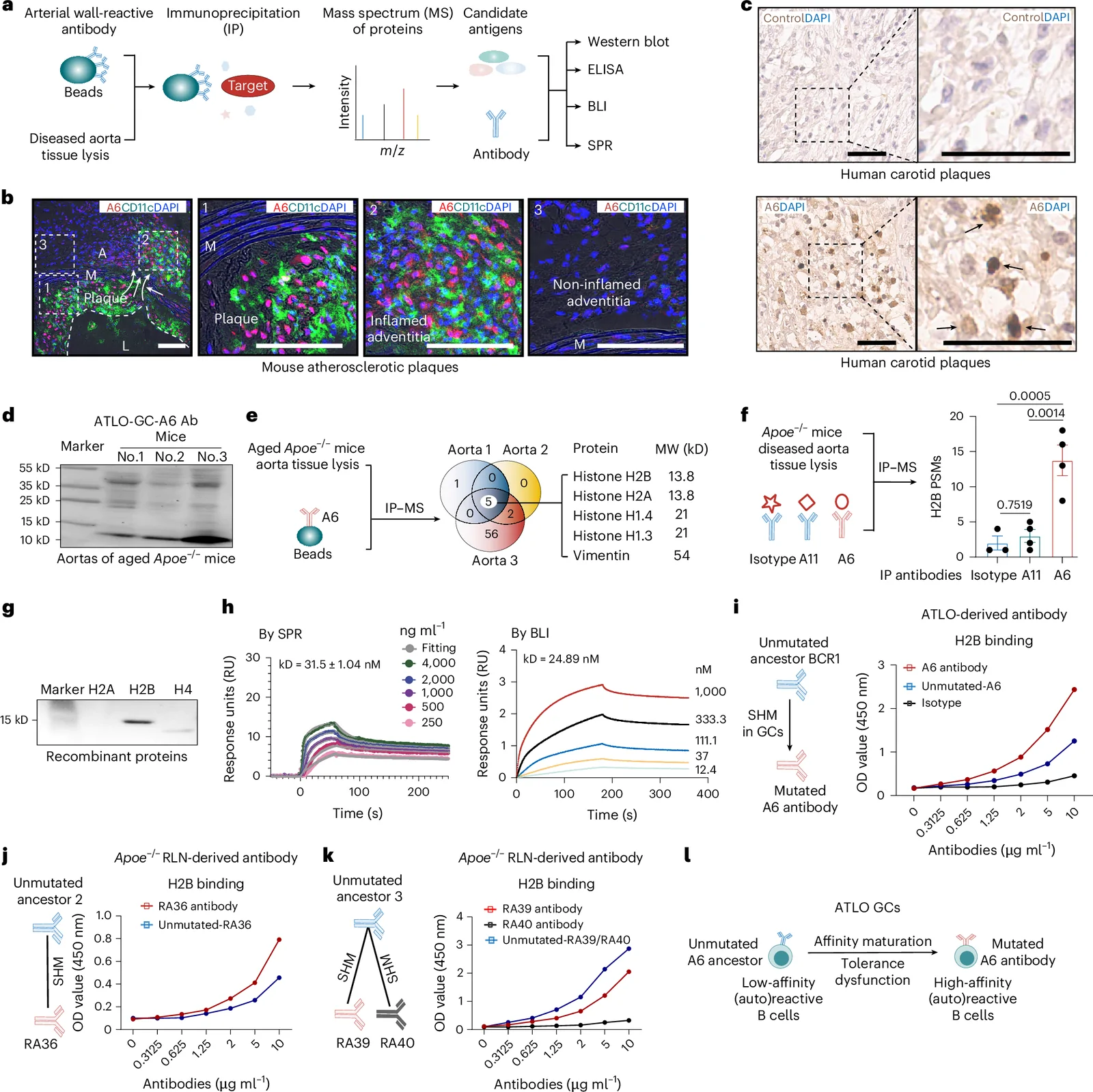
ATLO GCs give rise to a high-affinity autoantibody clone (termed A6) directed against H2B. **a**, Identification of the cognate autoantigen of A6. Schematic view shows the workflow to identify atherosclerosis autoantigens using diseased artery-detecting antibodies. A6-loaded protein A beads were incubated with tissue lysates prepared from diseased mouse arteries, followed by elution of proteins from the beads to undergo autoantigen screening of candidates by mass spectrometry (MS) analyses. Several autoantigen−antibody interaction assays were used to confirm protein−protein interaction in vitro, including western blots, ELISA, BLI and SPR analyses using various expression-cloned autoantibodies derived from RLNs and ATLO GCs. **b**, A6 reacts with mouse aorta-associated atherosclerotic plaques; co-staining of A6, macrophages/dendritic cells and nuclei in *Apoe*^−/−^ atherosclerosis plaque sections. A6 (red), CD11c (green) and nuclei (DAPI, blue). Bars, 100 μm. Representative images are shown from *n* = 5 mice. **c**, A6 reacts with human aorta or carotid artery atherosclerotic plaques; staining of A6 in human carotid atherosclerosis plaques by immunohistochemistry. Bars, 50 µm. Representative images are shown from *n* = 6 samples. **d**, A6 identifies a 10−15-kDa protein in mouse diseased artery lysates; western blot shows A6 staining of aorta tissues obtained from three individual aged *Apoe*^−/−^ mice. **e**, Immunoprecipitation (IP) combined with MS analyses to identify autoantigen candidates. The ATLO GC-derived A6 antibody was used as a bait to capture autoantigens from protein lysates of diseased aorta of 78-week aged *Apoe*^−/−^ mice. Beads without primary antibody were used as control. Label-free quantitation values of A6 were compared to controls. A6 antibody-enriched candidates are shown as a Venn diagram from three aorta samples from three individual aged *Apoe*^−/−^ mice. The molecular weight (MW) of each candidate protein is shown. **f**, A6 antibody captured H2B protein in the diseased aorta. The ATLO GC-derived A6 antibody was used as a bait to capture autoantigens from protein lysates of diseased aorta of 32-week *Apoe*^−/−^ mice. An isotype control antibody and the ATLO-GC-derived A11 antibody were used as controls. The peptide spectrum match values of H2B are shown. Each dot represents one individual biological repeat. Three 32-week-old *Apoe*^−/−^ aortas were combined as one sample. Data are presented as mean ± s.e.m. Statistical analyses were performed using one-way ANOVA with Tukey’s multiple comparison test. A two-sided *P* < 0.05 was considered significant. Isotype control (*n* = 3), A11 (*n* = 4) and A6 (*n* = 4). **g**, A6 binding to recombinant H2B by western blot; recombinant H2a, H2b and H4 were used for comparison. Experiments were repeated three times independently. **h**, A6 reacts with human recombinant H2B at high affinity; binding affinity of A6 to human recombinant H2B by SPR and BLI analyses. **i**, A6 acquires high-affinity binding to the H2B autoantigens via SHM from its unmutated ancestor; binding of ATLO GC-derived A6 antibody to H2B determined by ELISA: mutated A6 (red line), the unmutated parent of A6 (unmutated A6, blue line) and the isotype control antibody (black line). Each dot represents the mean of two technical replicates from one independent experiment. Experiments were performed independently three times. **j**,**k**, Anti-H2B autoantibodies isolated from *Apoe*^−/−^ RLN GC B cells (RA36, RA39, RA40). Binding of recombinant antibody to H2B determined by ELISA: mutated RA36/RA39/RA40 (red line); the unmutated parent of A6 (unmutated ancestors, blue line). Each dot represents the mean of two technical replicates from one independent experiment. Experiments were performed independently three times. **l**, Summary figure: A6 acquires high-affinity binding to H2B via GC responses, indicating tolerance dysfunction of ATLOs. A, adventitia; L, lumen; M, media; Ab, antibody; OD, optical density; PSM, peptide spectrum match. Source data

**Extended Data Fig. 7:**
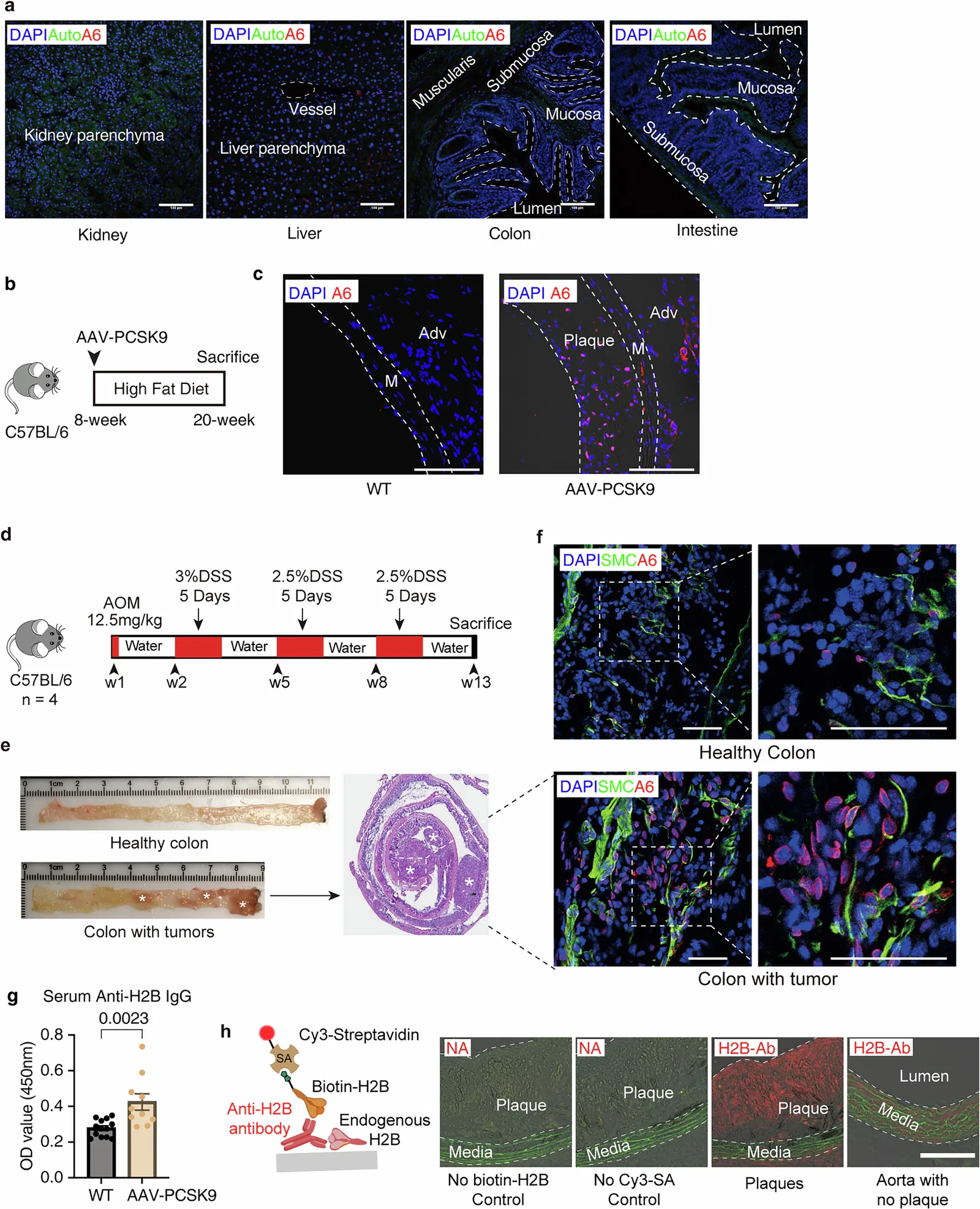
Characterization of ATLO GC B cell derived A6 antibody. (**a**) A6 staining in non-atherosclerotic tissues in WT mice. Representative images of IF staining of A6 antibody (25 µg/ml) in tissue array from aged WT mice. Kidney, liver, colon, and intestine tissues were collected from aged WT mice (n = 3). Tissues were prepared on one slide, and tissues were stained with identical conditions. A6 antibody was stained (red); Green autofluorescence signals were also acquired to delineate tissue background. nuclei were stained with DAPI (blue). Scale bars: 100 μm. (**b**) 8-week-old C57BL/6 mice were treated with AAV-PCSK9, meanwhile, these mice were fed with high fat diet for 12 weeks. (**c**) A6 antibody stains atherosclerotic plaques in AAV-PCSK9-induced atherosclerosis in C57BL/6 mice. This indicates H2B autoantigen is presented in plaques of this mouse model as well. Representative images are shown from *n* = 3 mice per group. (**d**) A6 stains tumor of colon cancer. Experimental design. C57BL/6 J mice were administered azoxymethane (AOM) -injection combined with three cycles of dextran sodium sulfate (DSS) treatment. After 13 weeks of treatment, all mice were sacrificed to collect colon tissues. (**e**) Representative image showing *en face* of colon from the lumen between healthy mice and mice with colorectal cancer. Multiple colon tumors (labeled with white asterisks) can be visualized at colon and were further confirmed by histological examination following hematoxylin staining. Control (n = 3), AOM-DSS treated mice group (n = 4). (**f**) Fresh frozen colon tissue sections were stained with A6 antibody (red) and smooth muscle cells (SMC, green). Representative images are shown from three WT mice and four colitis-associated cancer induced by AOM/DSS. Bar represents 50 μm. (**g**) Serum anti-H2B antibody titer was measured by ELISA. AAV-PCSK9-induced atherosclerosis in C57BL/6 mice and control mice were used. Each dot represents one individual mouse. Data are presented as mean ± SEM. Comparisons between two groups were performed using an unpaired two-tailed Student’s t-test. WT control (n = 13), AAV-PCSK9 (n = 10). (**h**) Detection of endogenous anti-H2B antibodies within atherosclerotic plaques in aged Apoe^−/−^ mice. Schematic view shows the workflow to detect anti-H2B antibodies in tissue sections: Biotin-labeled H2B recombinant protein was incubated with fresh frozen sectioned of mouse plaques, which allows the binding of H2B antigen to plaque endogenous anti-H2B antibodies *in situ*. Fluorescent dye labeled (Cy3 dye) streptavidin was used to detect biotin-H2B. Negative controls staining (including no bio-H2B antigen incubation, no cy3-streptavidin incubation) were used to show the staining specificity. Autofluorescence of collagen (green) was used to delineate the media layer of aorta. Phase contrast was used to delineate the tissue background. Aorta section without plaques was used to show the enrichment of endogenous anti-H2B antibodies within atherosclerotic plaques. Bar represents 100 µm. Representative images are shown from *n* = 5 mice per group. Source data

To examine the specificity of the ATLO GC-derived antibody responses, we focused on all 15 ATLO GC B cell-derived ‘atherosclerotic plaque-reactivity’ antibodies. We applied the following additional selection criteria: (1) plaque reactivity; (2) antigen specificity recognized by the antibody; (3) the identical antigen to also be presented in human plaques; and (4) western blots. A6 derived from ATLO GC B cells emerged as the top candidate that fulfilled all of the above criteria. Further studies revealed that A6 antibody reactivity is enriched in atherosclerotic plaques and the adjacent inflamed adventitia but much less or absent in non-inflamed arterial wall segments or non-inflamed tissues of aged *Apoe*^−/−^ mice (Fig. 5b, Extended Data Fig. 7a and Supplementary Table 1). Furthermore, A6 stained atherosclerotic plaque tissues in AAV-PCSK9-induced atherosclerosis^51^ (Extended Data Fig. 7b,c). These data indicate that the cognate antigen recognized by A6 is present in atherosclerotic plaques in both *Apoe*^−/−^ mice and AAV-PCSK9-injected C57BL/6 mice. Interestingly, A6 antibody also reacts with highly inflamed colon tissues where cell damage is prominent, as shown in a mouse colon cancer model, but to a much lesser degree in healthy colon (Extended Data Fig. 7d−f). The reactivity of A6 antibody with both inflamed colon and atherosclerotic plaque suggests that the target autoantigen(s) is not atherosclerosis specific but may be a general marker of cell damage and inflammation.

A6 antibody revealed a nuclear binding pattern (Fig. 5b) and also showed reactivity to nuclei in human atherosclerotic plaques (Fig. 5c), suggesting that the target autoantigen(s) recognized by A6 is also presented in human atherosclerosis. A6 recognized an approximately 10−15-kDa autoantigen in mouse atherosclerotic tissues (Fig. 5d). Pulldown of the A6 candidate autoantigens from mouse aorta extracts using immunoprecipitation followed by mass spectrometry (Fig. 5e,f) identified H2B as the cognate autoantigen for ATLO GC-derived A6 (Fig. 5e,f). Being phylogenetically ancient and also critical to maintain the three-dimensional structure of DNA helices, H2B is identical at the amino acid sequence level between mice and humans, explaining the mouse−human cross-immune reactivity of A6. H2B is a known autoantigen involved in several clinically important diseases, including systemic lupus erythematosus^52^. However, its participation in atherosclerosis autoreactivity has not been reported. We validated the binding characteristics of recombinant H2B to A6 by multiple methods, including western blot, surface plasmon resonance (SPR) interferometry and biolayer interferometry (BLI) (Fig. 5g,h).

Under homeostatic conditions, preventing the generation of high-affinity pathogenic self-reactive BCRs is indispensable for the maintenance of self-tolerance, although the underlying mechanisms are not completely understood^34,36,40^. Here, we observed that A6 bound recombinant H2B with high affinity (25−30 nM; Fig. 5h), suggesting that ATLO GCs allowed the survival of B cells expressing A6 high-affinity self (H2B)-autoreactive BCRs during affinity maturation. To directly test the impact of the ATLO GC reaction on affinity maturation, we generated the germline unmutated A6 precursor (unmutated A6 monoclonal antibody; Fig. 5i). Unmutated A6 showed a lower binding affinity to H2B than the highly mutated A6 (Fig. 5i), demonstrating that the ATLO GC-derived A6 had acquired high-affinity binding for H2B during ATLO GC SHM and affinity maturation.

We further analyzed the RLN GC B cell-derived antibodies and identified three autoantibodies reactive to H2B from *Apoe*^−/−^ RLN GC B cells—that is, RA36, RA39 and RA40 (Fig. 5j,k). RA36 acquired higher binding capacity to H2B versus their germline BCRs after SHM in *Apoe*^−/−^ RLN GCs; however, the binding capacity of RA36 to H2B turned out to be much lower than the ATLO GC B cell-derived A6 antibody, indicating that this is a low-affinity H2B autoantibody (Fig. 5j). Interestingly, we identified two further antibodies (RA39 and RA40) whose unmutated germline BCRs showed strong H2B binding capacity. These data confirmed that germline-derived high-affinity H2B autoantibodies undergo a reduction in affinity in RLN GCs. This phenotype has been termed ‘redemption’ (a mechanism to delete high-affinity binding of autoantibodies as one of many mechanisms to maintain tolerance to self^53^).

That B cells with BCRs encoding high-affinity A6 to the self-antigen H2B were found in ATLO GCs indicates that B cell immunity-specific dysregulation of redemption in ATLO GC reactions may have led to the formation of A6 (Fig. 5l).

### Humoral H2B autoimmunity promotes atherosclerosis in mice

**Fig. 6:**
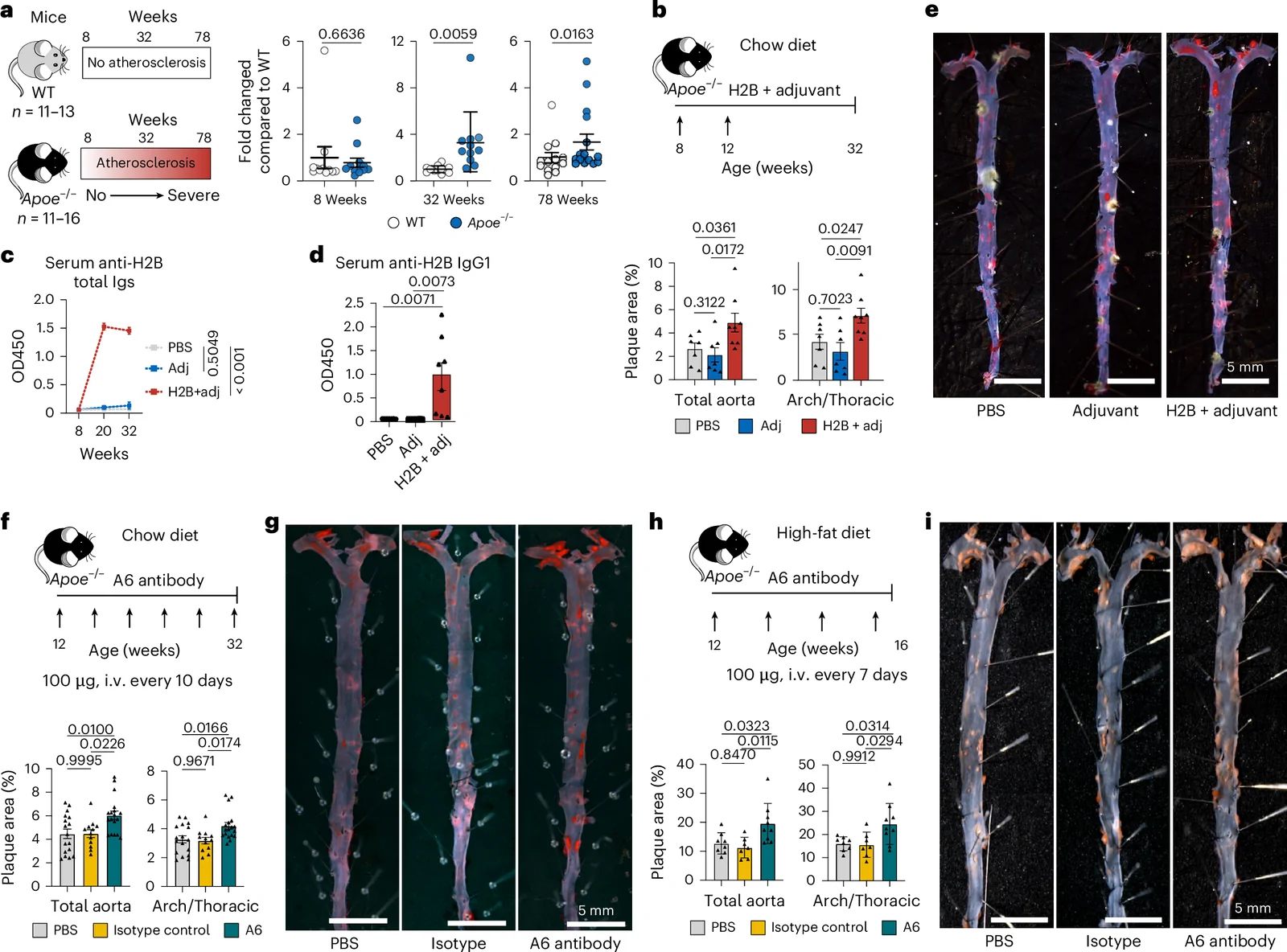
Atherosclerosis pathogenicity of the H2B−A6 pair. **a**, Anti-H2B autoantibody titers increase in *Apoe*^−/−^ mice during aging. Anti-H2B IgG1 serum antibody titers of WT and *Apoe*^−/−^ mice during aging were examined by ELISA; each dot represents one mouse. Data are presented as mean ± s.e.m. Eight-week WT (*n* = 11), 8-week *Apoe*^−/−^ (*n* = 12); 32-week WT (*n* = 12), 32-week *Apoe*^−/−^ (*n* = 11); 78-week WT (*n* = 13), 78-week *Apoe*^−/−^ (*n* = 16). Two-tailed Studentʼs *t*-test. **b**, H2B vaccination promotes atherosclerosis. Vaccination using recombinant H2B in young *Apoe*^−/−^ mice. *Apoe*^−/−^ mice were vaccinated with recombinant H2B with adjuvant (termed as H2B vaccine), adjuvant alone or PBS alone at the age of 8 weeks with a boost at the age of 12 weeks. The experiment was completed at 32 weeks. Blood was collected at different timepoints before and after the injection. **c**,**d**, H2B vaccination induced long-lasting T cell-dependent autoantibody responses in *Apoe*^−/−^ mice. Serum anti-H2B total antibody and anti-H2B IgG1 antibody titers were determined by ELISA. Data are presented as mean ± s.e.m. Statistical analyses were performed using two-way (**c**) or one-way (**d**) ANOVA with Tukey’s multiple comparison test. A two-sided *P* < 0.05 was considered significant. PBS control (*n* = 7), adjuvant (*n* = 7) and H2B+adjuvant (*n* = 8). **e**, En face staining for atherosclerotic plaques in the whole aorta. Atherosclerotic plaques were quantified, as described in the Methods. Scale bar, 5 mm. Data are presented as mean ± s.e.m. Statistical analyses were performed using one-way ANOVA with Tukey’s multiple comparison test. A two-sided *P* < 0.05 was considered significant. PBS control (*n* = 7), adjuvant control (n = 7), H2B+adjuvant (*n* = 8). **f**,**g**, A6 antibody transfer accelerated atherosclerosis in chow diet-fed *Apoe*^−/−^ mice. One hundred micrograms of A6 antibody was transferred via intraperitoneal injection at 12 weeks, and injections were repeated every 10 days. The experiment was completed at 32 weeks. Blood was collected at multiple timepoints before and after the injection; en face staining for whole aorta. Atherosclerotic plaques were quantified, as described in the Methods. Each dot represents one mouse. Scale bar, 5 mm. Data are presented as mean ± s.e.m. Statistical analyses were performed using one-way ANOVA with Tukey’s multiple comparison test. A two-sided *P* < 0.05 was considered significant. PBS control (*n* = 17), isotype antibody control (*n* = 12), A6 antibody (*n* = 18). **h**,**i**, A6 antibody transfer accelerated atherosclerosis in high-fat diet-fed *Apoe*^−/−^ mice. One hundred micrograms of A6 antibody was transferred via intraperitoneal injection at 12 weeks, and injections were repeated every 7 days. The experiment was completed at 16 weeks. Blood was collected at several timepoints before and after the injection; en face staining for whole aorta. Atherosclerotic plaques were quantified, as described in the Methods. Each dot represents one mouse. Scale bar, 5 mm. Data are presented as mean ± s.e.m. Statistical analyses were performed using one-way ANOVA with Tukey’s multiple comparison test. A two-sided *P* < 0.05 was considered significant. PBS control (*n* = 8), irrelevant isotype antibody control (*n* = 7) and A6 antibody (*n* = 9). Adj, adjuvant; i.v., intravenously; OD450, optical density at 450 nm. Source data

We next examined the pathogenicity of A6 antibody as well as its corresponding H2B autoantigen on atherosclerosis. We first determined the total anti-H2B antibody titers in WT and *Apoe*^−/−^ mice during aging (Fig. 6a). Serum anti-H2B IgG1 antibody (Fig. 6a) titers increased in *Apoe*^−/−^ mice during atherosclerosis progression versus age-matched WT controls. AAV-PCSK9-induced atherosclerotic mice also showed significantly higher levels of serum anti-H2B antibody levels (Extended Data Fig. 7g). These data indicated that T cell-dependent H2B autoimmune GC B cell responses and anti-H2B autoantibodies are associated with atherosclerosis disease severity in *Apoe*^−/−^ and AAV-PCSK9-injected C57BL/6 mice. Of note, endogenous anti-H2B antibodies are detectable within atherosclerotic plaques but not, or to a much lesser degree, in the aorta without plaques (Extended Data Fig. 7h), supporting the concept that circulating autoantibodies are present in atherosclerotic plaques^54^.

**Extended Data Fig. 8:**
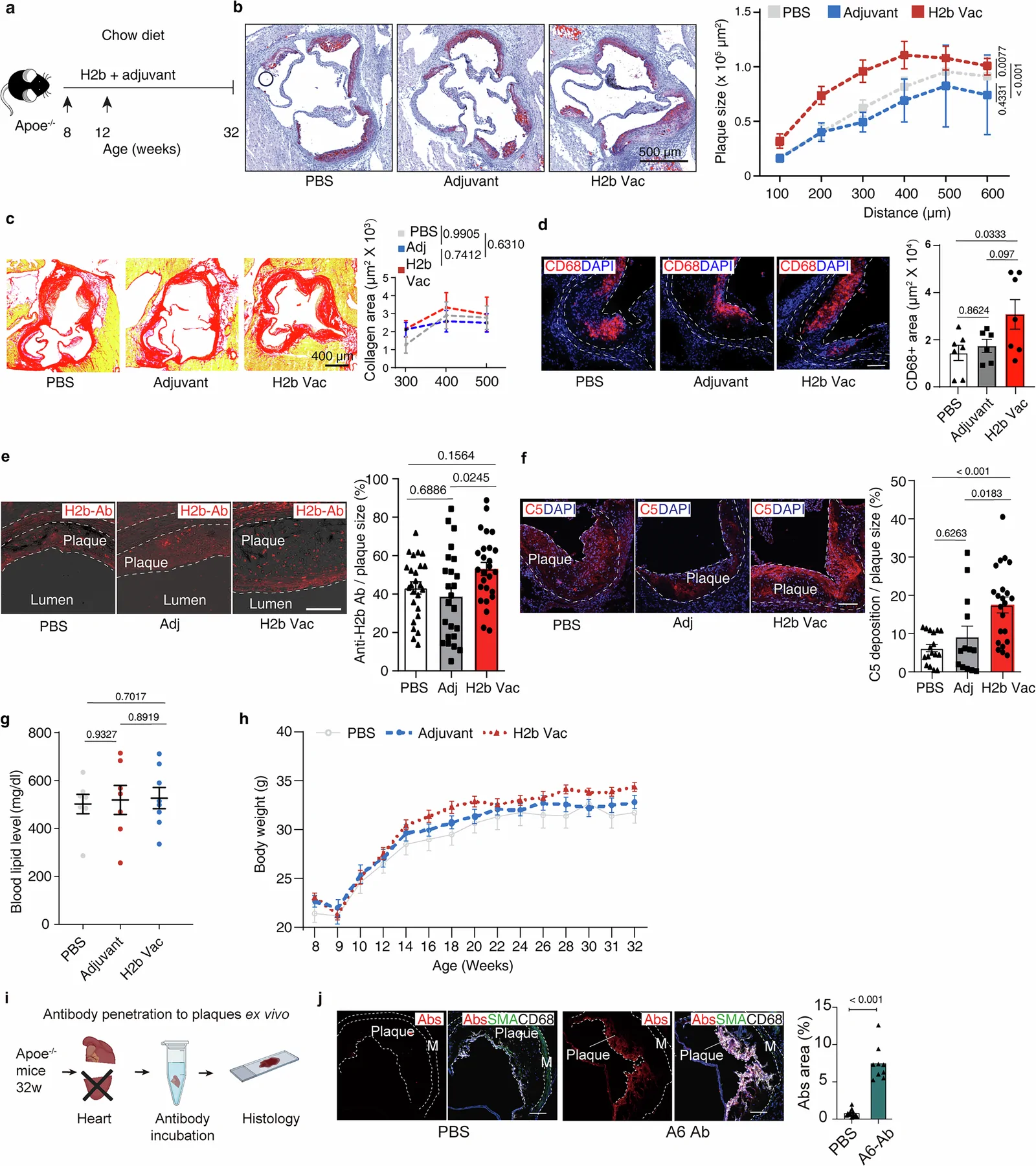
H2B autoimmune pathway does not impact blood lipids. (**a**) Experimental design of the H2B vaccination mouse model; vaccination using recombinant H2B in young Apoe^−/−^ mice. Apoe^−/−^ mice were vaccinated with recombinant H2B with adjuvant (termed as H2B vaccine), adjuvant alone, or PBS alone at the age of 8 weeks with a boost at the age of 12 weeks. The experiment was completed at 32 weeks. Blood was collected at multiple time points before and after the injection; (**b**) aortic root sections were stained for Oil red O (ORO). Plaque sizes were quantified as described in Methods; Scale bar 500 μm. Data are presented as mean ± SEM. Statistical analyses were performed using two-way ANOVA with Tukey’s multiple-comparison test. A two-sided P < 0.05 was considered significant. PBS control (n = 5), adjuvant control (n = 4), H2B + adjuvant (n = 6). (**c-d**) aortic root sections were stained for collagens and macrophages. Collagens sizes and macrophages (CD68^+^) were quantified as described in Methods; Scale bar 400 μm for collagen staining and 100 μm for CD68 staining. Data are presented as mean ± SEM. Statistical analyses were performed using two-way (c) or one-way (d) ANOVA with Tukey’s multiple-comparison test. A two-sided P < 0.05 was considered significant. For (c), PBS control (n = 7), adjuvant control (n = 6), H2B + adjuvant (n = 8). For (d), PBS control (n = 7), adjuvant control (n = 6), H2B + adjuvant (n = 7). (**e**) Endogenous anti-H2B antibodies within mouse atherosclerotic plaques were examined in H2B vaccinated mice. Quantification of plaque anti-H2B antibodies are shown. Each dots represents one plaque section, PBS (n = 4), Adjuvant (n = 3), H2B Vac (n = 5) mice per each group. Data are presented as mean ± SEM. Statistical analyses were performed using one-way ANOVA with Tukey’s multiple-comparison test. A two-sided P < 0.05 was considered significant. (**f**) Complement C5 protein deposition was examined within plaques. Quantification of plaque anti-H2B antibodies is shown. Statistical analyses used one-way ANOVA. Each dot represents one plaque section, PBS (n = 3), Adjuvant (n = 3), H2B Vac (n = 4) mice per each group. Data are presented as mean ± SEM. Statistical analyses were performed using one-way ANOVA with Tukey’s multiple-comparison test. A two-sided P < 0.05 was considered significant. (**g-h**) Vaccination of H2B in hyperlipidemic mice does not affect blood lipids (g) or body weight (h); blood was collected at the end of the experiment; body weight was measured at multiple time points before and after the injection. Data are presented as mean ± SEM. Statistical analyses were performed using one-way ANOVA with Tukey’s multiple-comparison test (g). A two-sided P < 0.05 was considered significant. PBS control (n = 7), adjuvant control (n = 7), H2B + adjuvant (n = 8). (**i**) A6 antibody penetrates atherosclerosis plaques *ex vivo*. Experimental design. Heart tissues with intact atherosclerosis plaques located in the aortic root were freshly isolated from 32 weeks Apoe^−/−^ mice. After the recombinant antibody incubation period, aortic root tissues sections were stained to detect the recombinant antibody by IF staining. (**j**) A6 antibody penetrated atherosclerosis plaques measured by IF staining. Each dot represents one mouse. Data are presented as mean ± SEM. Comparisons between two groups were performed using an unpaired two-tailed Student’s t-test. PBS control (n = 13), A6 antibody (n = 9). Source data

To study the potential pathogenicity of the H2B autoimmune responses in atherosclerosis, we established an H2B-induced vaccination protocol in *Apoe*^−/−^ mice. Recombinant H2B antigen and adjuvant (termed here as ‘H2B vaccine’) were given to 8-week-old *Apoe*^−/−^ mice—that is, before major atherosclerosis plaques emerge in large arteries in this mouse model. This initial vaccination was followed by a boost 4 weeks after the first injection. Six months after the first injection, mice were euthanized to determine atherosclerosis plaque loads (Fig. 6b). H2B vaccination significantly increased atherosclerotic plaque sizes in the whole aorta (Fig. 6b), including aortic roots and the thoracic and abdominal aorta segments (Extended Data Fig. 8a–d). Serum anti-H2B-specific antibody responses were determined by ELISA. The H2B vaccine induced long-lasting anti-H2B-specific antibody responses (Fig. 6c). Significantly higher levels of T cell-dependent IgG1-specific H2B antibody titers were observed in the H2B vaccine group when compared to controls (Fig. 6d), indicating that the H2B vaccine involved a T cell-dependent H2B-specific GC B cell responses in mice. We observed significantly more endogenous anti-H2B antibodies and complement C5 protein depositions in H2B vaccinated mice when compared to PBS or adjuvant only-treated mice (Extended Data Fig. 8e,f). These data support the concept that A6 and other autoantibodies directed against H2B form immune complexes to trigger complement activation and inflammation in atherosclerotic plaques. These observations are consistent with and extend our previous study that silencing complement C5 reduces atherosclerosis in *Apoe*^−/−^ mice^28^. Body weight and blood lipid levels remained unchanged (Extended Data Fig. 8g,h). They indicate that H2B autoimmune reactivity by A6 promotes atherosclerosis independent from lipid levels.

### A6 autoantibody transfer accelerates atherosclerosis in mice

**Extended Data Fig. 9:**
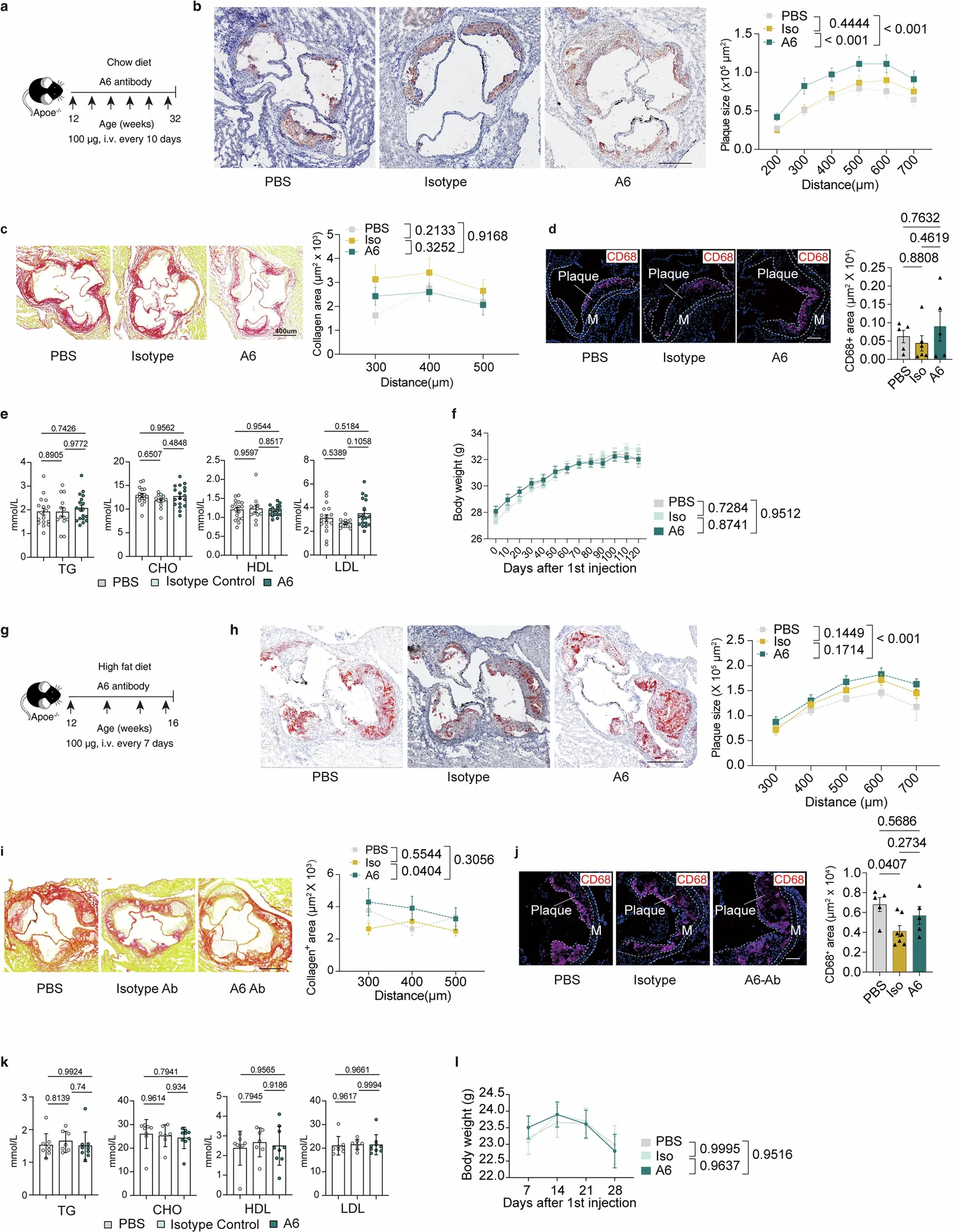
A6 autoantibody does not impact blood lipids. (**a**) Experimental design of the A6 monoclonal antibody treatment mouse model; A6 antibody transfer accelerated atherosclerosis in chow diet-fed Apoe^−/−^ mice. 100 µg A6 antibody was transferred via intraperitoneal (i.p.) injection at 10 weeks, and injections were repeated every 10 days. The experiment was completed at 32 weeks. (**b**) aortic root sections were stained for Oil red O (ORO). Plaque sizes were quantified as described in Methods; Scale bar 400 μm. Data are presented as mean ± SEM. Statistical analyses were performed using two-way ANOVA with Tukey’s multiple-comparison test. A two-sided P < 0.05 was considered significant. PBS control (n = 15), isotype antibody control (n = 12), A6 antibody (n = 14). (**c-d**) aortic root sections were stained for collagens and macrophages. Collagens sizes and macrophages (CD68^+^) were quantified as described in Methods; Scale bar 400 μm for collagen staining and 100 μm for CD68 staining. Data are presented as mean ± SEM. Statistical analyses were performed using two-way (c) or one-way (d) ANOVA with Tukey’s multiple-comparison test. A two-sided P < 0.05 was considered significant. For (c) PBS control (n = 5), isotype antibody control (n = 5), A6 antibody (n = 7). For (d) PBS control (n = 5), isotype antibody control (n = 6), A6 antibody (n = 5). (**e-f**) A6 antibody transfer does not affect blood lipids and body weight in Apoe^−/−^ few with chow diet. Triglyceride (TG), cholesterol (CHO), high-density lipoprotein cholesterol (HDL), and low-density lipoprotein cholesterol (LDL). Data are presented as mean ± SEM. Statistical analyses were performed using one-way (e) or two-way (f) ANOVA with Tukey’s multiple-comparison test. A two-sided P < 0.05 was considered significant. For (e) PBS control (n = 17), isotype antibody control (n = 12), A6 antibody (n = 18). For (f) PBS control (n = 17), isotype antibody control (n = 12), A6 antibody (n = 18). (**g**) A6 antibody transfer accelerated atherosclerosis in high fat diet-fed Apoe^−/−^ mice. 100 µg A6 antibody was transferred via i.p. injection at 12 weeks, and injections were repeated every 7 days. The experiment was completed at 16 weeks. Blood was collected at several points before and after the injection; PBS control (n = 8), irrelevant isotype antibody control (n = 7), A6 antibody (n = 9). (**h**) aortic root sections were stained for Oil red O (ORO). Plaque sizes were quantified as described in Methods; Scale bar 400 μm. Data are presented as mean ± SEM. Statistical analyses were performed using two-way ANOVA with Tukey’s multiple-comparison test. A two-sided P < 0.05 was considered significant. PBS control (n = 7), isotype antibody control (n = 7), A6 antibody (n = 8). (**i-j**) aortic root sections were stained for collagens and macrophages. Collagens sizes and macrophages (CD68^+^) were quantified as described in Methods; Scale bar 400 μm for collagen staining and 100 μm for CD68 staining. Data are presented as mean ± SEM. Statistical analyses were performed using two-way (i) or one-way (j) ANOVA with Tukey’s multiple-comparison test. A two-sided P < 0.05 was considered significant. For (i) PBS control (n = 5), isotype antibody control (n = 5), A6 antibody (n = 5). For (j) PBS control (n = 5), isotype antibody control (n = 7), A6 antibody (n = 5). (**k-l**) A6 antibody transfer does not affect blood lipids and body weight in Apoe^−/−^ few with high fat diet. Data are presented as mean ± SEM. Statistical analyses were performed using one-way ANOVA with Tukey’s multiple-comparison test. A two-sided P < 0.05 was considered significant. For (k) PBS control (n = 8), isotype antibody control (n = 7), A6 antibody (n = 9). For (l) PBS control (n = 8), isotype antibody control (n = 7), A6 antibody (n = 9). Source data

A6 antibody can penetrate the plaque ex vivo (Extended Data Fig. 8i,j). To examine potential mechanisms of A6 high-affinity autoantibody actions, in preliminary studies we transferred A6 to two widely used mouse models of atherosclerosis—that is, young *Apoe*^−/−^ mice maintained on either chow or high-fat diet, respectively (Fig. 6e,f). A6 antibody transfer significantly increased atherosclerosis plaque loads under both dietary regimens compared to saline or irrelevant isotype controls (Fig. 6e,f and Extended Data Fig. 9). Mouse body weights and blood lipid levels were not affected by the A6 transfer (Extended Data Fig. 9e,f,k,l). More work is needed to understand the mechanisms of A6 pathogenicity, however. These data show that A6 high-affinity autoantibody is pathogenic and promotes atherosclerosis in mice.

### Serum anti-H2B antibody titers are associated with aortic calcification in human atherosclerosis

**Fig. 7:**
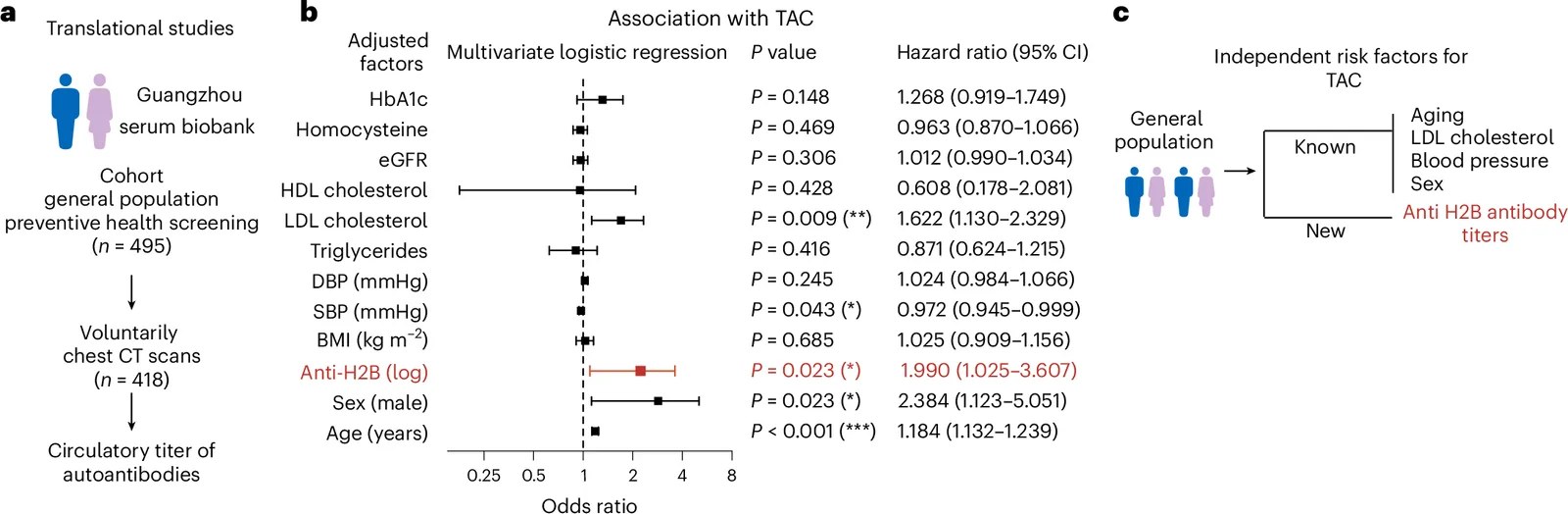
Serum anti-H2B antibody titers are associated with TAC. **a**, Study design of the clinical cohort. A cross-sectional cohort of 495 individuals of the general population aged 30−70 years who underwent routine medical screening of preventive healthcare was studied. Exclusion criteria included patients with previously diagnosed cardiovascular diseases, strokes, autoimmune diseases requiring systemic treatment, active malignancies and severe kidney or liver dysfunctions. As part of the preventive health screening program, 84.4% (418/495) of individuals voluntarily underwent chest three-dimentional CT scans. Serum anti-H2B antibody titers were examined. **b**, Serum anti-H2B antibody titers is an independent risk factor for TAC. Anti-H2B antibody titers were logarithmically transformed before entering the model to approximate Gaussian distribution. Odds ratios and 95% confidence intervals were calculated by binary logistic regression with simultaneous entry of all variables (Cox−Snell *R*^2^ = 0.296, *P* < 0.001). **c**, Serum anti-H2B antibody is an independent risk factor for TAC. CI, confidence interval; DBP, diastolic blood pressure; SBP, systolic blood pressure.

Finally, we tested whether the humoral autoimmune response to H2B is associated with vascular pathologies in humans (Fig. 7a). Serum anti-H2B antibody titers were determined in a cross-sectional cohort of 495 individuals representative of the general population aged 30−70 years who underwent chest computed tomography (CT) scans as part of a routine medical preventive screening program (Fig. 7a; demographic and clinical characteristics in Supplementary Table 2). Higher serum anti-H2B antibody titers were associated with the prevalence of thoracic aortic calcification (TAC) (Fig. 7b). TAC is widely used in chest CT screening and is used as a non-invasive diagnostic tool before coronary angiography and stent implantation in clinical settings^55^. The prevalence of TAC was also significantly associated with age, male sex, low-density lipoprotein (LDL) cholesterol and systolic blood pressure (Fig. 7b). However, the association between TAC and anti-H2B antibody titers persisted (*P* = 0.023) to be statistically significant even after adjustments for the traditional risk factors, including age, male sex, LDL cholesterol, systolic blood pressure, glycated hemoglobin A1c (HbA1c) (reflecting long-term plasma glucose homeostasis) and estimated glomerular filtration rate (eGFR, reflecting kidney function) by logistic regression analyses (Fig. 7b). We further performed sensitivity analyses by including high-sensitivity C-reactive protein (hsCRP) as a covariate in a subcohort sample. Our analyses confirmed a significant association between anti-H2B antibody titers and TAC (odds ratio = 2.43, *P* = 0.023; Supplementary Table 3). Notably, anti-H2B antibody and hsCRP showed no correlation (*r* = 0.046, *P* = 0.434). These data indicated that mechanisms independent of conventional risk factors for aortic calcification may explain this association, and these data are consistent with the possibility that humoral autoimmunity to H2B may contribute to atherosclerosis in humans (Fig. 7c).

## Discussion

**Extended Data Fig. 10:**
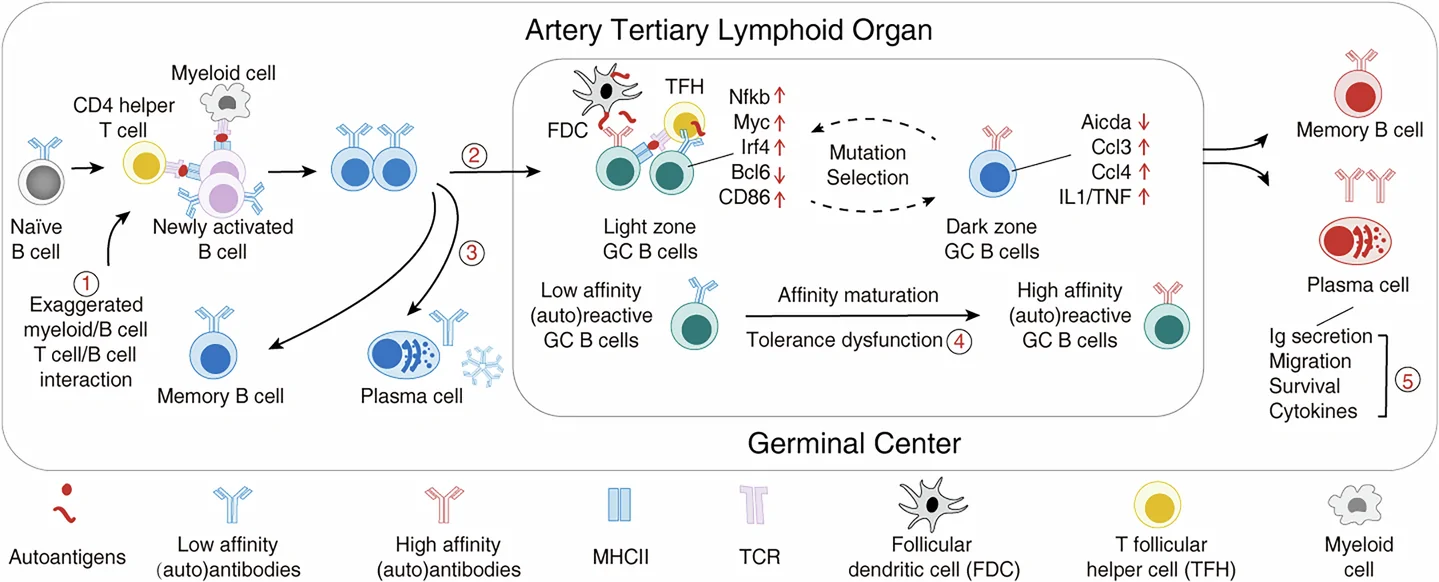
Hypothetical choreography of B cell Immunity in ATLOs. A fraction of naïve B cells after entering the ATLO find cognate (auto)antigens and - upon subsequent help received from CD4^+^ T helper cells and TFH cells - become newly activated B cells followed by proliferation; some of these B cells may differentiate into Mem B cells or PCs without entering the GC; others are recruited into the GC to advance to early GC B cells; low-affinity early GC B cells subsequently engage in the GC high affinity maturation cycle via interaction with cognate (auto)antigens bound to the surface of FDCs and they also receive helper signals from TFHs; after repeated cell cycles, some of these GC B cells become Mem B cells or PCs, while others are deleted or silenced via tolerance mechanisms; Multiple tolerance checkpoints are compromised at ATLOs: (1) Exaggerated myeloid/B cell and T cell/B cell interactions at pre-GC phase; (2) Exaggerated B cell recruitment of newly activated B cells into the GC and (3) differentiation of newly activated B cells that do not enter the GC into extrafollicular PC formation pathway; (4) dysregulation of GC B cell tolerance permitting formation of high affinity autoantibodies for self-antigen during affinity maturation; (5) formation of abnormally large PC niches in post-GC events. ATLO-specific B cell dysfunction is consistent with the possibility that ATLOs are preferred lymphoid organs to generate atherosclerosis-directed high affinity autoantibodies against.

Here we identify a pathogenic high-affinity autoantibody, A6, and its cognate self-autoantigen, H2B, as an endogenous ATLO-derived and atherosclerosis-relevant autoantibody−autoantigen pair. Vaccinating *Apoe*^−/−^ mice with H2B or adoptive transfer of A6 both significantly exacerbated atherosclerosis, and antibody titers to H2B in a human cohort were positively correlated with TAC. A6 is a high-affinity pathogenic autoantibody cloned from paired heavy and light chain BCR sequences from ATLO GC B cells. Together with the results of our clinical trial, this provided evidence for the possibility that ATLOs are lymphoid tissues with an impaired tolerance to self (Extended Data Fig. 10). This conclusion is supported by significant transcriptomic differences between ATLO GC B cells versus GC B cells in lymph nodes—that is, *Bcl6*, *Cxcr4* and *Nfkb1*. Among all B cell subsets, the rare GC B cell subset undergoing affinity maturation and somatic mutation is the only B cell subset that does not circulate among different tissues, including lymph nodes, other SLOs and ATLOs^40,56^. By contrast, the majority of all other B cell subsets, including Mem B cells and PCs, express both emigration and homing receptors (*S1pr1*, *Sell*), allowing them to circulate between tissues^40,56^. Therefore, our strategy to focus on GC B cells as well as their autoantibodies allows us to pinpoint the differences observed on B cells that had selectively generated in ATLOs versus those in lymph nodes. As expression-cloned ATLO GC B cells were skewed to recognize atherosclerosis-related or arterial wall-derived autoantigens, further work will focus on GC B cells and other B cell subsets in ATLOs, including Mem B cells and PCs, to identify additional high-affinity autoantibody−autoantigen pairs that may protect or accelerate the disease.

The generation of a high-affinity autoantibody, lower levels of SHM by GC B cells and higher levels of SHM by PCs in ATLOs deserve attention. These data may indicate a dysbalance of release mechanisms from the GC reaction of Mem B cells versus PCs to enter the non-ATLO GC compartment. Affinity-dependent Mem B cell and PC release mechanisms have been reported in GCs of SLOs but not in TLOs. High-affinity BCRs are selected to be released from GCs as PCs, whereas intermediate/lower-affinity BCRs are selected to be released as Mem B cells from GCs^34,36^. Our model of ‘accelerated GC B cell−PC differentiation pathways in ATLOs’ may explain our observation that ATLO GC B cells have lower SHM and form high-affinity mutated antibodies, such as A6. The model is supported by the phylogenetic tree construction data tracing B cell clone origins in RLNs versus their counterparts in ATLOs. However, other possibilities may exist to explain this phenotype. For example, recent studies have shown that high-affinity antibody-producing GC B cells silence their SHM rates during vaccination but divide more rigorously when compared to their lower-affinity counterparts^57,58^.

Autoantibodies in atherosclerotic mice and humans have been described for decades^4^, including IgG or IgM antibodies against oxLDL, polyclonal antibodies to β2GPI^13^, GRP78 (ref. ^14^) or CXCR3 (ref. ^15^) and monoclonal antibodies to ALDH4A1, Hsp60 or Hsp70 (refs. ^16,17^). The pathogenicity of these autoantibodies in atherosclerosis is dependent on their isotypes and their cognate antigens^8–10^. However, their affinities to the respective self-antigens have not been determined, and their clinical relevance to directly affect the disease has remained unclear. Notably, none of these previous studies identified an endogenous pathogenic autoantibody−autoantigen pair in atherosclerosis with defined specificity and affinity. First, we used an unbiased approach to clone the candidate antibodies from BCR heavy and light chain sequences. Second, we screened for plaque reactivity and against general tissue reactivity. Third, because we know the origin of each B cell based on the experimental design and the transcriptomes of the B cell subsets, we can pinpoint the origin of the A6 high-affinity autoantibody to ATLO GC B cells (Extended Data Fig. 10). ATLOs are atherosclerosis specific in that they are not found in healthy arteries^7^. Here, we show that atherosclerosis is home of this A6−H2B pathogenic autoantibody−autoantigen pair, which is a child of ATLOs.

As little is known about the specific disease impact of any TLO in any disease^23,24^, the underlying mechanisms controlling the generation of high-affinity pathogenic autoantibody by ATLO GC B cells in atherosclerosis identified here may apply to other TLO-associated diseases^23,24,43,59,60^. Furthermore, it would be of considerable interest to compare mechanisms of tolerance control in TLO-associated conditions with opposite prognostic disease outcomes, such as in most types of cancers with protective versus those with detrimental outcomes as in autoimmune diseases and atherosclerosis^23,29,43,61^. We hypothesize that TLOs of diverse diseases containing GCs in activated B cell follicles and separate T cell areas may be similar to some ATLO traits described here: all form in response to chronic inflammation close to or within the diseased target tissue; all are home to a large innate inflammatory immune cell compartment; all have separate T cell and B cell areas; and many are home to FDCs in addition to developing structures to promote T cell and B cell homing, such as high endothelial venules^23,24,29^. Although we suspect similarities of B cell tolerance mechanisms in TLO-related diseases other than atherosclerosis that show activated B cell follicles with GCs, there may also be significant dissimilarities: these may be related to different immune cell compositions regarding T cell and/or B cell subtypes as well as the nature of the autoantigens differentially driving these diseases, as well as the non-immune cell tissue cells in which TLOs emerge. For example, in atherosclerosis, CD8^+^ T cells may promote atherosclerosis while cancer autoantigen-specific CD8^+^ T cells may be beneficial to eliminate cancer cells. Recently, one of us showed that a tumor TLO PC-derived antibody is involved in controlling tumor growth^43^.

Our discovery of a high-affinity and pathogenic autoantigen−autoantibody pair in ATLOs represents, to our knowledge, the first identification of such a pair in any TLO of any organ or disease. Thus, we hypothesize that the immune cell regulatory balance in ATLOs may not be as tightly controlled when compared to SLOs. Moreover, DEGs in multiple immune cell transcriptomes and prominent innate immune cells, such as multiple macrophage subsets, indicate persistent and marked chronic inflammation in TLOs when compared to SLOs. Both the innate and the adaptive immune cell compartments are different in ATLOs versus SLOs in addition to each cell subtypeʼs differential transcriptomes.

The nature of the atherosclerosis-relevant antigen-presenting cells is not known, but we speculate that they may be some kind of a dendritic cell subtype. TLOs are enriched with inflammatory macrophages and γδ T cells^7,43,62^, and it is unknown how this trait may alter the immune environment in ATLOs or other TLOs. Because our approach is, in principle, applicable to other TLO-related diseases and disease models and humans, high-affinity autoantibodies can perhaps be cloned from cancer TLOs or autoimmune diseased tissues. We propose that data described here may also have the potential to understand the opposite clinical correlations and disease outcomes in different diseases—that is, dysregulated tolerance mechanisms of B cell immunity as evidenced by the generation of high-affinity antibodies may be beneficial in cancers^24,31,43^—but these same traits may play detrimental roles to promote autoimmune diseases^26,30^.

Breaking of tolerance to self has previously been shown in CD4^+^ and CD8^+^ T cells in a mouse model of atherosclerosis^7^ and in human plaques^63^. Once T cell tolerance is lost, self-reactive CD4^+^ T cells may differentiate into T_FH_ cells and support affinity maturation of B cells in GCs. In a large number of T cells, tolerance to self is encoded in the transcriptome of the peripheral naive CD4^+^ repertoire^64^. Naive self-reactive CD4^+^ T cells express more CD73 and PD-1 than naive non-self-reactive CD4^+^ T cells, and blocking these molecules unleashes a CD4^+^ T cell-triggered autoimmune response. It remains to be seen whether a loss of FoxP3 in CD4^+^ T cells in ATLOs^7^ may be sufficient to allow autoreactive B cells to develop, proliferate and become antibody-secreting PCs.

H2B is not atherosclerosis specific, but it is a known autoantigen with strong antigenic and proinflammatory features, and it is emerging in many autoimmune diseases, including systemic lupus erythematosus and others as well as cancer^52^. The reactivity of A6 with both inflamed colon and atherosclerotic plaques suggests that H2B and one of its epitopes are likely to be recognized by the immune system. Nuclear DNA is normally tightly coiled around histones. Our findings suggest that H2B may become ‘visible’ to the immune system in atherosclerotic lesions and ATLOs, but the underlying mechanisms of this visibility remain unknown. This could be related to neutrophil extracellular traps (NETs) that are known to occur in atherosclerosis^65^ or some form of post-translational modification such as those reported for autoimmune diseases. However, to our knowledge, neither the pathogenicity nor the affinity of any of these multiple anti-H2B autoantibodies has been determined. The study of A6 provides proof-of-principle evidence for a pathogenic high-affinity autoantibody in atherosclerosis research. Moreover, A6 is not the only expression-cloned autoantibody but one of 30 studied. It is conceivable to suggest that some of these ATLO GC-derived autoantibodies may be protective or pathogenic or not functional for disease progression.

In conclusion, the discovery of pathogenic high-affinity autoantibodies to H2B provides a potentially important element supporting the autoimmune component of clinically significant atherosclerosis. These data will be taken as the basis to develop H2B-specific therapeutics aiming to lower serum anti-H2B autoantibody levels.

### Limitations of the study

Limitations of this study include that it was performed in a mouse model of atherosclerosis, which does not match all aspects of human atherosclerosis. However, the discovery that H2B antibody titers are positively correlated with TAC in humans suggest that our findings are translatable. Another limitation is that our study was not designed or powered to discover gender differences. Similarly, our clinical cohort reveals an independent association between H2B antibody titers and TAC; the fully adjusted regression model also showed a negative association between systolic blood pressure and TAC. Our study design and cohort were not sufficiently powered to examine interactions among all parameters, and future studies in larger clinical cohorts will, therefore, be required to address this apparent counterintuitive finding. Autoimmune diseases are more common in women than men, but atherosclerosis appears earlier in men than in women. We do not know at what point in time autoimmunity to H2B develops in the atherosclerosis environment. Another important limitation of the present study relates to the small number of ATLO GC B cells and that Mem B cells and PCs may contain high-affinity autoantibodies with atherosclerosis relevance. Identification of the entire autoimmune reactome, therefore, needs to be addressed in future studies to distinguish high-affinity pathogenic versus high-affinity protective autoantibodies during atherosclerosis progression. Finally, we observed dysregulated gene expression networks in ATLOs, including higher expression levels of cytokine genes (*Ccl3*, *Ccl4*, *Ccl6*) by GC B cells and lower expression levels of cytokine genes (*Il4*, *Il21*) by T_FH_ cells. Therefore, extensive protein validation and functional studies will be warranted in future studies.

## Methods

### Mice

WT and *Apoe*^−/−^ mice on a C57BL/6 background were raised in a specific pathogen-free environment at the animal facilities of Ludwig-Maximilians-Universität München (LMU) until 78 weeks of age or at the animal facilities of The First Affiliated Hospital of Sun Yat-sen University at 8−32 weeks of age. Mice were given standard rodent chow or a high-fat diet containing 40% fat and 1.25% cholesterol (Research Diets) and maintained in a climate-controlled room at 23 °C and 60% relative humidity, with a 12-hour light/dark cycle. The animal procedures were authorized by the Regierung of Oberbayern or by The First Affiliated Hospital of Sun Yat-sen University, following the guidelines of the local Animal Use and Care Committee and national animal welfare laws. To establish an atherosclerosis mouse model, 8-week-old male C57BL/6 mice were injected with AAV-PCSK9 (Vigene Biosciences, AAV8-PCSK9-D377Y) via the tail vein at a dose of 1.5 × 10^11^ vector genomes per mouse. On the same day as the viral injection, mice were switched to a high-fat diet and maintained on this diet for 12 weeks.

### Tissue collection

Mice were anesthetized using hydrochloride and xylazine hydrochloride mixed solution (2:1) through intraperitoneal injection. The peritoneal cavity was washed with 10 ml of FACS buffer (DPBS buffer with 2% FBS) to obtain lavage; blood was collected by cardiac puncture. After perfusion with 10 ml of EDTA buffer (5 mM), 20 ml of PBS and 20 ml of FACS buffer, RLNs, spleen and ATLO were isolated. All harvested tissues were immediately placed on ice for subsequent processing. ATLO tissues from the diseased aorta for scRNA-seq and scBCR-seq were collected: In short, adipose tissue and para-aortic lymph nodes were carefully removed. The whole aorta was dissected and collected in cell culture dishes with pre-cooled FACS buffer. The aorta was opened in the longitudinal direction, and plaques were carefully removed using curved forceps (Dumont 5/45; Fine Science Tools) under the dissecting microscope. The remaining aorta was collected as ATLOs. Aortas with no ATLOs (as indicated with no atherosclerosis) were excluded from the subsequent studies. Two separate cohorts of mice were used: (1) pools of three *Apoe*^−/−^ and three WT mice were collected for ATLOs and RLNs, and pools of five *Apoe*^−/−^ and five WT mice were collected for blood in the first cohort; (2) cell hashtags (BioLegend, TotalSeq C) were used to label cells of four *Apoe*^−/−^ and four WT mice individually to collect ATLOs and RLNs as reported previously^7^.

### Human carotid artery tissues

Human carotid atherosclerotic plaques were obtained from patients with high-grade carotid artery stenosis (>70%) after carotid endarterectomy and were provided by the Biobank Munich, Technical University Munich (TUM), according to the guidelines. The study was performed according to the guidelines of the World Medical Association Declaration of Helsinki. The ethics committee of the Faculty of Medicine, TUM, approved the study, and written informed consent for permission to be included into the Munich Vascular Biobank was given by all patients.

### Clinical cohort methods

We conducted a cross-sectional study of 495 individuals from the general population (registration number: ChiCTR2400089577) who underwent routine medical screening of preventive healthcare at The First Affiliated Hospital of Sun Yat-sen University. The study was approved by the Clinical Research and Laboratory Animal Ethics Committee (approval ID: [2024]374) at The First Affiliated Hospital of Sun Yat-sen University, and all participants provided informed consent to participate. The baseline demographic and clinical characteristics of the participants are summarized in Supplementary Table 2. Exclusion criteria included patients with previously diagnosed cardiovascular diseases, strokes, autoimmune diseases requiring systemic treatment, active malignancies and severe kidney or liver dysfunction. Blood samples were collected to assess general clinical laboratory parameters and serum anti-H2B antibody titers. Additionally, as part of medical screening program, chest three-dimensional CT scans were performed voluntarily for general diagnostic purposes. Non-contrast-enhanced chest CT scans were performed as part of the clinical assessment using a 64-row multidetector CT scanner. The scanned area started from the apex to the base of the lungs, including the heart and aortic arch, and was acquired in the craniocaudal direction during inspiration. The scan parameters were as follows: tube voltage, 120 kV, and tube current, 200 mAs; beam pitch, 0.828; rotation time, 0.5 seconds; beam collimation, 64 mm × 0.5 mm. TAC was considered a binary outcome defined by the presence of hyperattenuating signals (above the threshold of 100 Hounsfield units and assessed by experienced radiologists) in an area of at least three adjacent pixels within the boundaries of the thoracic aorta (ascending, arch and descending aorta). Valid CT scan data were available for 418 individuals. Regression analyses were performed with SPSS Statistics version 31.0 (IBM Corp.). The univariate association between anti-H2B antibody titers and TAC was analyzed using univariate binary logistic regression. For multivariate analyses, a logistic regression model was developed by simultaneous entry of log-transformed anti-H2B antibody titer (to approximate a Gaussian distribution), sex, age, body mass index (BMI), systolic blood pressure, diastolic blood pressure, high-density lipoprotein (HDL) cholesterol, LDL cholesterol, triglycerides, eGFR, homocysteine and HbA1c as covariates. Total cholesterol was excluded from the model due to significant collinearity with LDL cholesterol. In a sensitivity analysis on a subcohort of 289 individuals, hsCRP was included as an additional covariate. The results of the simultaneous-entry model were confirmed using a backward logistic regression approach. In this model, sex, age, anti-H2B antibody titer, LDL cholesterol and HbA1c were retained as independent predictors in the final step.

### Single-cell suspension preparation

Tissues were dissected into small pieces and mashed to allow passage through 70-µm^2^ strainers. Aorta samples were subjected to digestion with 2.5 ml of enzyme cocktail (400 U ml^−1^ Collagenase Type I, 10 U ml^−1^ Collagenase Type XI, 60 U ml^−1^ hyaluronidase, 60 U ml^−^^1^ DNase I and 20 mM HEPES in DPBS) at 37 °C for 45 minutes with slow shaking prior to filtering. The filtered cell suspension was subsequently centrifuged at 300*g* for 5 minutes. After removal of the supernatant, 5 ml of ACK lysis buffer (0.15 mM NH_4_Cl, 1 mM KHCO_3_, 0.1 mM Na_2_EDTA, pH 7.2−7.4) was added to spleen, blood and bone marrow samples to induce lysis of red blood cells. This lysis process lasted for 5 minutes at room temperature, followed by two rounds of washing to eliminate cell debris and residual lysis buffer. Cells were then resuspended in 1 ml of pre-cooled FACS buffer and passed through 100-µm^2^ filters before cell counting. The cell number was determined using a Neubauer chamber under the microscope or by an automated cell counter (Bio-Rad, TC20).

### Single-cell isolation for single-cell profiling

Samples were prepared as follows. After the single-cell suspension preparation, cells of each tissue were stained with fixable viability dye (1:1,000 dilution; eBioscience) in PBS at 4 °C for 20 minutes. After washing and blocking steps, cells were stained with CD45 labeled as fluorescence (1:200 dilution; BD Biosciences, clone 30-F11, for pooling cohort) or CD45.2 (1:200 dilution; BioLegend, clone 104, for hashtag cohort) with or without hashtagged CD45/MHC-I antibody (1:50 dilution; BioLegend, TotalSeq C) at 4 °C for 30 minutes. Single CD45^+^ live cells (see gating strategy in Supplementary Fig. 1) were sorted using a flow cytometer (BD Biosciences, FACSAria III). Single CD45^+^ live cells were sorted into a cold tube pre-rinsed with FACS buffer using a flow cytometer (BD Biosciences, FACSAria III). Cell number and viability were determined by using an automated cell counter (Bio-Rad, TC20). The cells were maintained on ice until loaded to the Chromium controller.

### Single GC B cell isolation for polymerase chain reaction cloning

After preparation of single-cell suspensions from ATLOs and RLNs, cells were stained with FVD (APC-Cy7-conjugated, 1:1,000 dilution; eBioscience) to label live cells at 4 °C for 20 minutes, followed with CD45 PercP-Cy5.5 (30-F11; eBioscience), CD19 APC (1D3; eBioscience), IgD^−^eFluor 450 (11-26c; eBioscience), GL7-Biotin (GL7; eBioscience), PNA-FITC (Vector Laboratories) and streptavidin-APC-eFluor780 (eBioscience) in pre-cooled FACS buffer. Single GC B cells (FVD^−^CD45^+^CD19^+^IgD^−^GL7^+^PNA^+^; gating strategy in Supplementary Fig. 1) were sorted into pre-cooled 96-well plates containing 4 µl of lysis buffer (final concentrations: 0.5× DPBS without Ca/Mg, 3 U µl^−1^ RNasin Plus RNase inhibitor (Promega, cat. no. N2615) and 10 mM dithiothreitol (DTT)). The 96-well plates were sealed with Microseal ‘F’ Film (Bio-Rad) and immediately frozen on dry ice before storage at −80 °C.

### Single B cell cDNA synthesis for polymerase chain reaction cloning

Total RNA from a single GC B cell on a 96-well plate was reverse transcribed in RNA/DNA-free water using 128 ng of random primers (Invitrogen, 48190-011), 16.2 U of RNasin Plus RNase inhibitor (Promega), 0.3% IGEPAL CA-630 (Sigma-Aldrich), 100 mM DTT, 12.6 mM dNTP and 50 U of SuperScript III reverse transcriptase (Invitrogen). Reverse transcription reactions were performed at 42 °C for 5 minutes, 25 °C for 10 minutes, 50 °C for 60 minutes and 94 °C for 5 minutes. cDNA was stored at −20 °C before polymerase chain reaction (PCR).

### Cloning of paired heavy and light chains of BCRs from single GC B cells

PCR primers used for single-mouse GC B cell cloning were synthesized by Metabion. The final concentration of all primers was 100 μM. Mouse BCR heavy chain and BCR light chains were amplified independently by nested PCR starting from 4 µl of cDNA as template. All PCR reactions were performed in 96-well plates in a total volume of 42 µl of PCR buffer containing 0.3 µM µl^−1^ dNTP, 0.3 µM each primer or primer mix and 0.05 U µl^−1^ Hot Start Taq enzyme (Qiagen, cat. no. 203203). Semi-nested or nested second-round PCR was performed with 4 μl of unpurified first-round PCR product at 95 °C for 15 minutes, followed by 50 cycles of 94 °C for 30 seconds, 60 °C (IgH) or 58 °C (IgL) for 30 seconds, 72 °C for 45 seconds and final incubation at 72 °C for 10 minutes. PCR products were purified by purification kit (Qiagen) according to the manufacturer’s instructions. Purified DNA was sequenced by Sanger sequencing at the Faculty of Biology, LMU (http://www.gi.bio.lmu.de/sequencing). BCR sequences were further compared to the germline line BCR sequences from the IMGT databank (https://www.imgt.org/). Paired BCR heavy chain and light chain sequences were used to generate monoclonal antibodies.

### Single-cell profiling library preparation for gene expression and BCR-seq

Cell concentrations were adjusted to 700-1,200 cells per microliter according to the protocol of 10x Genomics 5′ library and gel bead kit. Single-cell suspensions were mixed with nuclease-free water and 5′ single-cell RNA master mixture and then loaded into a Chromium chip with barcoded gel beads and partitioning oil. The chip was placed in the Chromium controller or Chromium X to generate gel beads-in-emulsion (GEMs). cDNA was obtained from 100−180 μl of GEMs per sample by reverse transcription reactions: 53 °C for 45 minutes, 85 °C for 5 minutes and then maintained at 4 °C. cDNA products were purified and cleaned using Dynabeads. cDNA was amplified by PCR: 98 °C for 45 seconds; 98 °C for 20 seconds, 67 °C for 30 seconds, 72 °C for 1 minute and amplified for 13–16 cycles; and then 72 °C for 1 minute. Amplified PCR products were cleaned and purified using SPRIselect reagent kit (Beckman Coulter, B23317). The concentration of cDNA library was determined by Qubit dsDNA HS Assay Kit (Invitrogen). The single-cell transcriptome library and B cell V(D)J library were prepared using Chromium single-cell V(D)J reagent kits following the standard guidance. In total, 3,000 single cells were expected to be recovered for each sample. The reverse transcription and following library construction were performed using a C1000 Touch thermal cycler (Bio-Rad). Fourteen cycles were set for cDNA amplification. The concentration of each library was determined using Qubit 3.0 fluorometer and NEBNext Library Quant kit, and the size range of final libraries was checked on a fragment analyzer (Advanced Analytical Technologies). 5′ gene expression libraries or BCR libraries were pooled together with the same quality for sequencing.

### Next-generation sequencing

The concentration of pooled libraries was quantified using both Qubit 3.0 fluorometer and NEBNext Library Quant kit. The size of libraries was assessed using Bioanalyzer (Agilent Technologies). Only libraries of high quality were loaded onto the Illumina platform. For the first cohort of pooled samples of scRNA-seq and scBCR-seq, 5′ gene expression libraries and BCR libraries were sequenced separately at IMGM. Around 1% PhiX control library spike-in (Illumina) was added to 5′ gene expression libraries and sequenced by NextSeq high-output reagent kit version 2.5 (150 cycles) using the NextSeq 500 platform (Illumina). Sequencing was performed as an asymmetrical manner: read 1 was adjusted to 26 bp, read 2 to 98 bp and index 1 to 8 bp. BCR libraries together with 1% PhiX control library spike-in were sequenced in a symmetrical manner (150 bp + 150 bp, paired end). For the second cohort of hashtagged samples, 5′ gene expression libraries, BCR libraries and cell hashtag libraries were sequenced together using the NovaSeq 6000 platform at IMGM. Three types of libraries were pooled at the ratio of 40:10:1. Additionally, 1% of the PhiX library (Illumina) was spiked in as a control. Sequencing was performed in an asymmetrical manner: read 1 was adjusted to 28 bp + 10 bp for i7 index, 10 bp for i5 index and read 2 to 90 bp.

### Single-cell sequencing data pre-processing analysis

The next-generation sequencing data were demultiplexed based on Illumina index using 10x Genomics Cell Ranger mkfastq pipeline (version 3.0.0). The 5′ gene expression matrix was generated by aligning the data to mm10 genome reference using the STAR aligner built into Cell Ranger. Count matrix of each sample was loaded into R (version 4.2.1) software using the Seurat (version 3.4.0) package. Cells containing fewer than 200 genes, more than 4,000 detected genes or greater than 8% of mitochondrial genes were filtered out as suspected doublets or debris. Data from different tissues were log normalized, and 2,000 highly variable features were calculated to integrate data. After integration, principal component analysis (PCA) was performed for following dimensional reduction and clustering. Twenty dims were included to reduce the dimension and assign clusters. The clustering result was visualized using UMAP dimensionality reduction methods built in Seurat. To focus on B subsets, clusters that highly expressed *Cd19* or *Sdc1* (CD138) genes were extracted and reclustered for the following analyses. GC B cells were extracted and integrated with published data (GSE154634) following the integration pipelines provided by the Seurat package. Then, unsupervised clustering of all integrated GC B cells was performed using FindClusters function with a resolution parameter of 0.2. The phase of cell cycle was annotated by calculating the expression of cell-cycle-related markers using the cell cycle scoring pipelines in the Seurat package. PCs were extracted to be reclustered base on 0.6 resolution. The BCR sequences were assembled and annotated referring to GRCm38 reference. The information of BCR sequences in each B cell was integrated into metadata of 5′ gene expression data for further analysis. CD4^+^ T cells were retrieved from the single-cell dataset reported in our previous publication (see ‘Data availability’ section) and reanalyzed via unsupervised clustering. T_FH_ cells were defined by the expression of well-characterized T_FH_-associated markers and genes implicated in T_FH_ functions.

### DEGs and pathway enrichment analyses

In scRNA-seq analysis, DEGs for each tissue or each B cell subset were determined with the following criteria: |log(fold change)| > 0.25 and adjusted *P* < 0.05. The normalized expression of DEGs in each cell was visualized with the DoHeatmap function built in the Seurat package. DEGs obtained from each B cell subsets were parsed to the clusterProfiler package for functional enrichment and multiple comparisons. Terms showing a false discovery rate (FDR) less than 0.05 were considered as significant. Selected enriched terms were illustrated by ggplot2. The results of GSEA were also obtained using the clusterProfiler package, and the GSEA plot for selective Gene Ontology term was visualized by the enrichplot package.

### Differential abundance analysis of cell populations

Differential abundance of B cell subsets was assessed based on raw cell counts using the edgeR package. A negative binomial model was fitted with a design matrix including tissue type and batch effects. Statistical significance was determined by likelihood ratio tests with Benjamini−Hochberg correction. Results were visualized using ggplot2, with color indicating log fold change and point size representing the absolute log fold change.

### Clonal diversity analysis and rarefaction/extrapolation

Clonal diversity within each B cell subset was evaluated using multiple diversity metrics. Rarefaction and extrapolation analyses were performed with the iNEXT R package to estimate clonal diversity across a standardized range of sampling depths. Simpson diversity was calculated based on clonotype abundance, and both rarefaction (solid lines) and extrapolation (dashed lines) curves were generated. Confidence intervals were estimated by bootstrap resampling and visualized as shaded areas. All diversity metrics were visualized using ggplot2.

### Lineage tree construction

Within each clonotype, full-length immunoglobulin heavy chain nucleotide sequences were compared, and pairwise sequence differences were used to infer lineage relationships. Lineage trees were constructed by connecting sequences according to nucleotide divergence and visualized using the igraph package in R. In each lineage tree, the number of cells corresponding to each cell type was quantified, and their relative proportions were calculated for each tissue. Aggregated cell type proportions were visualized as pie charts using ggplot2.

### BCR mutation analyses

Sequences of each unique BCR heavy chain were realigned with the IMGT/HighV-QUEST database to summarize the number of mutations in V region. The number of mutations in each cell was visualized with dot plots using ggplot2.

### CSR event analyses

The distribution of class-switched isotypes was summarized using pie charts, where each slice represents the proportion of different isotypes. The overall size of each pie chart was scaled according to the total number of cells in the corresponding sample.

### Clonotype tracking analyses

To identify shared clonotypes across different cell types or tissues, clonotype overlap between sample pairs was assessed using the VennDiagram package in R. The fraction of overlapping clonotypes was calculated based on the resulting intersection tables and subsequently visualized using the circlize package.

### Cell−cell interaction modeling analyses

The count of cell−cell interactions was predicted and visualized with the CellChat (version 2.1.2) package in R. The ligand−receptor interaction pairs were analyzed using CellPhoneDB (version 5). Specifically, gene symbols in our dataset were converted to corresponding human gene symbols using biomaRt (version 2.54.1). Genes that did not match human genes were removed, and, in cases where multiple human genes matched, only one was retained. The gene expression count matrix and cell annotation data (metadata) were used as input for analysis in each tissue. The cell−cell interaction analysis was performed using Python (3.8.13) with default settings. For visualization, interaction pairs with an interaction score greater than 0 and *P* values less than 0.05 were considered significant. *P* values less than 0.0001 were represented as 0.0001 in the dot plot for better visualization.

### Spatial cell−cell interaction analysis

Aorta from 78-week-old *Apoe*^−/−^ mice were dissected and processed following the 10x Genomics Visium HD Fixed Frozen Tissue Preparation Handbook (CG000764, Rev A) to prepare the spatial transcriptional library. The library was sequenced on a NovaSeq X Plus platform according to the manufacturer’s instructions. Raw sequencing reads were aligned to probe sequences and mapped to the corresponding CytAssist image using Space Ranger (version 3.1.2). Nucleus segmentation was performed using a Python script based on StarDist and provided by 10x Genomics (https://www.10xgenomics.com/cn/analysis-guides/segmentation-visium-hd). The resulting gene-by-binned barcode data were analyzed in R (version 4.4.3) for cell annotation and visualization. Cell deconvolution was conducted using robust cell type decomposition (RCTD, spacexr package, version 2.2.1), leveraging an integrated scRNA-seq dataset from atherosclerotic tissues as the reference for cell type annotation. Spatial cell−cell interaction analysis was performed using spateo (version 1.1.0) on the nucleus-segmented AnnData object obtained from the above steps. Spatial neighborhoods were constructed for each cell using *k*-nearest neighbors based on physical coordinates calculated by the squidpy package (version 1.6.1). Candidate ligand−receptor interactions between predefined sender and receiver cell type groups were then identified using the ‘st.tl.find_cci_two_group’ function. A curated mouse-specific ligand−receptor interaction pairs database derived from CellTalkDB was used as input to define candidate ligand−receptor pairs. Only ligand−receptor pairs with significant spatial co-expression were retained and used for visualization.

### Immunization *Apoe*^−/−^ mice with H2B protein

Equal amounts of TiterMax@Gold Adjuvant (Sigma-Aldrich, T2684-1ML) and recombinant H2B protein (Active Motif, https://www.activemotif.com/) was used to prepare the adjuvant−H2B emulsion. After preparing the adjuvant−H2B emulsion, 8-week-old *Apoe*^−/−^ mouse received 200 μl of PBS alone, adjuvant or adjuvant−H2B (25 µg per mouse) emulsion by intraperitoneal injection. Four weeks after the primary injection, H2B (25 µg per mouse) in 200 μl of PBS was used as the booster injection. The control group received the same volume of PBS. Every 1 or 2 weeks, the body weight was measured. Blood was collected through the tail vein to measure the titer of antibodies and cholesterol levels. Animals were euthanized at 32 weeks of age. Whole aortas and aortic roots were collected to quantify atherosclerosis.

### Methods for detecting tissue endogenous H2B autoantibodies

Endogenous anti-H2B antibodies within mouse atherosclerotic plaques were examined in H2B vaccinated mice. Fresh-frozen sections (10 µm) of atherosclerotic plaques from H2B vaccinated *Apoe*^−/−^ mice were prepared by a cryostat and stored at −80 °C. Tissue sections were heated on a hotplate at 37 °C for 1 minute and air dried at room temperature for 30 minutes. Sections were rinsed in PBS for 10 minutes. Sections were blocked according to the kit manufacturer’s instructions (Endogenous Biotin-Blocking Kit; Thermo Fisher Scientific, E21390) to minimize non-specific staining caused by endogenous biotin and biotin-binding proteins in tissues. Biotinylated H2B recombinant protein was incubated overnight at 4 °C in a humid chamber, followed by rinsing with PBS. Sections were incubated with Cy3-labeled streptavidin (Jackson ImmunoResearch, AB_2337244) for 60 minutes in the dark after washing three times with PBS. Sections were fixed in 2% paraformaldehyde (PFA) after the staining and wash steps and then mounted in mounting medium (Vector Laboratories). For negative controls, staining was performed without H2B recombinant protein biotinylated or Cy3-streptavidin dye. Stained sections were analyzed using a confocal laser scanning microscope (Leica, SP8 3X). All images were prepared as TIFF files by ImageJ or Leica LAS-X (version 1.2) software and exported into Adobe IIIustrator CS6 for figure arrangements.

### A6 antibody penetration assay of atherosclerotic plaques ex vivo

Mice were euthanized, and the heart was rapidly excised and gently rinsed in ice-cold PBS to remove residual blood. The heart was then transected horizontally at the mid-ventricular level; the apical portion was discarded; and only the upper half containing the intact aortic root region was retained to preserve the architecture of the aortic sinus-associated plaques as intact tissues. The tissues were incubated PBS buffer with chimeric A6 recombinant monoclonal antibody (mouse A6 antibody variable region and the human antibody constant regions). After 2-hour incubation at 37 °C (to mimic the approximate physiological temperature), the tissues were thoroughly washed with PBS to remove surface unbound antibody. Frozen tissue sections were prepared. The fluorophore-conjugated secondary antibody recognizing human antibody constant regions was used to analyze antibody penetration into atherosclerotic plaques ex vivo.

### Transfer of A6 antibody in *Apoe*^−/−^ mice

Plasmids encoding the heavy (IgH) and light (IgL) chains of the A6 antibody were transfected into Expi293F cells (Thermo Fisher Scientific, A14527) using polyethyleneimine (Sigma-Aldrich) as the transfection reagent. Seven days after transfection, the cell culture supernatant was harvested and purified using a Protein A Sepharose (Sigma-Aldrich, P3391) chromatography column. The eluate was collected and transferred into pre-soaked dialysis membranes (Spectrum Labs, 132554), which were placed in PBS and gently stirred overnight at 4 °C. The dialyzed antibodies were then concentrated using Amicon Ultra Centrifugal Filters with a 30-kDa molecular weight cutoff (Millipore, UFC903024). IgG concentrations were determined by ELISA, and the purity of IgG was assessed by SDS-PAGE (4−12%). Low/no endotoxin levels (<5 EU ml^−1^) were confirmed by endotoxin assay kit (Thermo Fisher Scientific, A39552). To study the effect of A6 antibody in atherosclerosis mice, twelve-week-old *Apoe*^−/−^ male mice were maintained on a chow diet for 20 weeks. Every 10 days, 100 μg of A6 antibody (mA6-IgG2a) or mouse IgG2a isotype antibody (Abinvivo, B115101) or equal volume of PBS control were intravenously administered. After 20 weeks of treatment, the mice were euthanized for tissue collection. To study the effect of A6 in a high-fat diet-induced atherosclerosis mouse model, 10-week-old *Apoe*^−/−^ male mice were fed a high-fat diet for 6 weeks. Then, 100 μg of A6 antibody (mA6-IgG2a) or mouse IgG2a isotype antibody (Abinvivo, B115101) or equal volume of PBS control was intravenously administered at 12 weeks. After 4 weeks of treatment, the mice were euthanized for tissue collection.

### Atherosclerosis quantification

En face Sudan IV and Oil-Red-O (ORO) staining were used to analyze the mouse aortas. To assess lipid-rich areas in the whole aorta (en face staining), arch, thoracic and abdominal parts, a digital camera (Leica) was used to capture images with a standard bar. ORO staining was used to analyze atherosclerotic plaque areas. The Thunder imaging system (Leica) was used to capture ORO staining images. ImageJ was used to measure the percentages of lipid deposition in the whole aorta, arch, thoracic, abdominal parts and aortic root and quantified atherosclerotic lesion areas.

### Determination of antibody−antigen binding affinity by SPR

SPR analyses were performed using Biacore X100 (Cytiva) equipped with a research-grade C1 sensor chip. The ligand was immobilized using amine-coupling chemistry at a density of 492 resonance units (RUs) on flow cell 2; flow cell 1 was left empty to be used as a reference surface. Analytes in running buffer (PBST: 0.1% Tween 20 with 0.05% BSA in PBS, pH 7.4) were injected at a flow rate of 90 μl min^−1^. The complex was allowed to associate and dissociate for 60 seconds and 240 seconds, respectively. The surfaces were regenerated with two pulses (60 seconds) of 30 mM NaOH, 2 M NaCl and 0.3% Triton X-100. Responses from analyte injections (colored curves) were overlaid with the fit of the 1: 1 interaction model (shown as red curve) determined using Biacore X100 evaluation 2.0 software.

### Examination of antibody−antigen binding affinity by BLI

The BLI analyses were performed using an Octet R8 (Sartorius) equipped with research-grade Protein A biosensors (Sartorius, 18-5010). Different antibodies (for example, A6 and isotypes) were coupled to Protein A biosensors, and antigen−antibody affinity was analyzed using the same concentration gradient of H2B as the analyte. Protein A biosensors were hydrated for 10 minutes in DPBS (Gibco, 70011044) before loading 10 µg ml^−1^ antibody for 120 seconds. After a 60-second baseline step, protein analyte was associated for 180 seconds to the sensor, followed by a 180-second dissociation step. *K*_d_ values were determined with a global fit 1:1 binding algorithm using Octet Analysis Studio software.

### Western blot

Aortas were lysed in ice-cold radioimmunoprecipitation assay lysis buffer containing a protease inhibitor cocktail after removing blood. Equal amounts of protein lysates were separated on the gel and transferred from the gel to the membrane. Membranes were subsequently blocked for 2 hours at room temperature in 5% BSA blocking buffer. After that, the membrane was incubated with primary antibodies. The membrane was washed with TBST and incubated with the horseradish peroxidase (HRP)-conjugated secondary antibody in blocking buffer at room temperature for 1 hour. The last step was to wash the membrane three times with TBST for 10 minutes. The HRP chemiluminescent substrate (Thermo Fisher Scientific) working solution was prepared by mixture of equal parts stable peroxide solution and enhanced substrate solution in a clear tube (600 μl per membrane) and added to the membrane. CCD camera imaging devices (Fuji, LAS3000 Imaging System) were used to detect chemiluminescence.

### Antibody polyreactivity assay by ELISA

Antibody binding capacity to three structural unrelated proteins was determined by ELISA, including dsDNA, LPS and insulin. ELISA plates (Costar, 9018) were prepared by coating with 5 μg ml^−1^ human recombinant insulin (Yeasen Biotechnology, 40112ES25) or 10 μg ml^−1^ dsDNA (Sigma-Aldrich, D4522) or 10 μg ml^−1^ LPS (Sigma-Aldrich, L2630) in a 0.05 M carbonate-bicarbonate buffer (Sigma-Aldrich, C3041-50CAP) and incubated overnight at room temperature. Subsequent blocking was achieved using 2% BSA. Antibodies were applied at a concentration of 4 μg ml^−1^, followed by three serial dilutions (1:4), and incubated at room temperature for 1 hour. Detection of bound antibodies was performed using a rabbit anti-human IgG HRP-conjugated antibody (Abcam, ab6759) and an HRP substrate (Sigma-Aldrich, T4444). Optical density at 450 nm was measured using a Multiskan SkyHigh (Thermo Fisher Scientific). RA32 antibody was cloned from *Apoe*^−/−^ RLN GC B cells; RW49 antibody was cloned from WT RLN GC B cells; and AT5 antibody was cloned from ATLO GC B cells. RA32 antibody recognizing all three antigens strongly was used as high positive control. AT5 antibody did not bind to all three antigens, and was used as negative control. The detailed amino acid sequences of the paired full length of variable region of IgH and IgL are listed below:

RA32-IgH-QVQLRQPGIELAKSGTSVKLSCKASGFTFTSYWMHWVRQRPGQGLEWIGHINPNKDGINFNERFKTKATLTVDRSSATAFMQLSSLTSADSAVYYCARWGPGYFDYWGQGTTLTVSS;

RA32-IgL- DLVLTQSPASVAVSPGQRATISCRVSESIDNFGISFMNWFQQKPGQPPKLLIYFASKQGSGVPARFTGSGSGTNFSLNIHPMEEDDTAMYFCQQSKEVPYTFGGGTKLEIK;

RW49-IgH- EVQLVESGGGLVKPGGSLKLSCAASGFTFSDYGMHWVRQAPEKGLEWVSYISSGSSTIYYADTVKGRFTISRDNAKNTLFLQMTSLRSEDTAMYYCARPGYYGLYAMDYWGQGTSVTVSS;

RW49-IgL- DIKMTQSPSSMYASLGERVTITCKASQDINSYLSWFQQKPGRSPKTLIFRANRLVDGVPSRFSGSGSGQDYSLTISSLEYEDMGIYYCLQYVEFPHTFGGGTKLEIK

AT5-IgH-QVQLQQPGAELVKPGASLKLSCQASGYIFTSYWLHWVKQRPGQGLEWIGMILPNSGSTYYNEEFKTKAALTVDKSSSTAYMQLTSLTSEDSAVYYCARSTTVVASPYVMDYWGQGTSVTVSS

AT5-IgL-DIVLTQSPATLSVTPGDSVSLSCRASQNISNNLHWCQQKSHESPRLLIKYASQSISGIPSRFSGSGSGADFTLSLNSVETEDFGMYFCQQTNSWPLTFGAGTKLELK

### Immunoprecipitation and protein mass spectrometry

Protein A (HÖLZEL Diagnostika, L00273) and Dynabeads Protein G (Thermo Fisher Scientific, 10004D) were used to capture ATLO-derived antibodies through binding to the Fc regions of antibodies. First, 50 μl of total protein A/G beads was taken (that is, 25 μl of protein A + 25 μl of protein G) into a clean tube and washed three times with 500 μl of immunoprecipitation buffer (10 mM HEPES, pH 7.6, 150 mM NaCl, 12.5 mM MgCl_2_, 0.1 mM EDTA, 0.5 mM EGTA, 10% glycerol) for 5 minutes at room temperature. The tube was placed on the magnet, and the supernatant was removed in each wash. After that, 8 μg of antibodies in 500 μl of immunoprecipitation buffer was added to couple to the 50 μl of beads and incubated on a rotating wheel overnight at 4 °C. On the second day, after washing three times with immunoprecipitation buffer, protein extract was added. The samples were incubated for 4 hours on a rotating wheel at 4 °C. Then, samples were washed three times with immunoprecipitation buffer with 5−10- minute incubations on a rotating wheel at 4 °C. To the tube was added 50 mM NH_4_HCO_3_ buffer to resuspend the samples, and the tube was placed on the magnet to remove the supernatant. After the second NH_4_HCO_3_ wash, 1 ml of NH_4_HCO_3_ was added to resuspend the beads and transferred to a fresh tube. The tube was placed on the magnet to remove as much supernatant as possible with 200-μl pipette tips. The samples were digested for 30 minutes at 25 °C at 800 r.p.m. with 100 μl (or enough to cover the beads) of 10 ng μl^−1^ trypsin in 1 M urea in 50 mM NH_4_CO_3_. The supernatant was collected into a fresh tube. The beads were washed twice with 50 μl of 50 mM NH_4_HCO_3_, and the supernatants were pooled into the corresponding tube. DTT was added to the samples to a final concentration of 1 mM DTT and digested overnight at 25 °C at 800 r.p.m. After the digestion, alkylation reduction was performed by adding 10 μl of iodoacetamide (5 mg ml^−1^) and incubating for 30 minutes in the dark at 25 °C, followed by 1 μl of 1 M DTT for 10 minutes at 25 °C. Samples were acidified using 2.5 μl of trifluoroacetic acid (TFA). Peptides were then loaded to C18 stage tips (for desalting and purification) that were prior washed using 20 μl each of methanol, 80% acetonitrile−0.1% TFA and 0.1% TFA only. After binding of the peptides to the C18 stage tips, they were washed three times with 20 μl of 0.1% TFA, followed by a fast spin at 11,000*g* for 5 minutes. Samples were eluted by 3 × 15 μl of 80% acetonitrile−0.25% TFA. They were then dried using a SpeedVac centrifuge and resuspended in 15 μl of 0.1% TFA. The desalted peptides were injected and separated on an UltiMate 3000 RSLCnano (Dionex) at Biomedical Center Munich Molecular Biology, LMU. Proteins were identified and quantified using MaxQuant version 1.6.17.0. Proteins with a log_2_ fold change greater than 1 (indicating enrichment of the candidates in aorta sample when compared to control) were identified for each sample. Venn diagram analysis was performed to identify shared candidates by three independent aortas.

### ELISA for anti-H2B in the circulation

To determine the titer of anti-H2B antibodies in the circulation of humans or mice, 100 μl of purified recombinant H2B proteins (Active Motif, cat. 31892) at 2 μg ml^−1^ concentration in the carbonate-bicarbonate buffer (Sigma-Aldrich, C3041-50CAP) were immobilized on high binding capacity microliter plates (Costar, 9018) overnight at 4 °C. Then, the microliter plates were blocked with 300 μl per well of 2% BSA for at least 2 hours at room temperature. Subsequently, H2B-coated plates were incubated for 1 hour at room temperature with 100 μl of recombinant anti-H2B antibody as standard (recombinant anti-H2B antibody was diluted stepwise to 250 ng ml^−1^, 100 ng ml^−1^, 50 ng ml^−1^, 25 ng ml^−1^, 10 ng ml^−1^ and 5 ng ml^−1^ in antibody dilution buffer) or serum from 8, 32 and 78 weeks WT and *Apoe*^−/−^ mice (at 1:250 dilution) and humans with atherosclerosis (at 1:200 dilution). The plates were incubated with HRP-conjugated secondary antibodies (1:2,000; SouthernBiotech) for 1 hour at room temperature in the dark. In the end, 100 μl per well of TMB (Kementec Diagnostics, DY999) was used to incubate plates for 5−15 minutes at room temperature. The 2 N sulfuric acid (100 μl per well) was used to stop the ELISA reaction at an optical density value between 1 and 3. The absorbance was measured at 450 nm using a multi-well plate reader (Tecan, SpectraFluor Plus).

The standard curve was drawn based on known recombinant anti-H2B antibody concentrations plotted against the absorbance values after subtracting the optical density value from the background value (no antibody background). Sigmoidal 4-parameter was used to fit the standard curve in GraphPad Prism 6. The serum sample background-subtracted optical density values were inputted into Prism, and the concentration of serum samples was reported as ng ml^−1^.

### Immunofluorescence staining

For immunofluorescence staining, tissues were dissected and embedded in Tissue-Tec (Sakura Finetek), frozen in isopentane and kept at −80 °C. Immunofluorescence staining was performed using ATLO GC-derived and RLN GC-derived antibodies, anti-mouse CD11c (Serotec, N418), α-smooth muscle actin (SMA; Sigma-Aldrich, 1A4), CXCR4 (Abcam, UMB2), RelB (Santa Cruz Biotechnology, C-19), CD35 (BD Biosciences, 8C12) and BCL6 (BD Biosciences, K112-91). Fresh-frozen sections (10 µm) from mouse tissues and human carotid plaques were prepared by a cryostat and stored at −80 °C. Tissue sections were heated on the hotplate at 37 °C for 1 minute and air dried at room temperature for 30 minutes. Sections were fixed with 4% PFA for 3 minutes at 4 °C, followed by rinsing with PBS. After diving in acetone for 2 minutes at room temperature, the sections were rinsed in PBS for 10 minutes. Primary antibodies (diluted in PBS with 0.25% BSA) were incubated overnight at 4 °C in a humid chamber. Sections were incubated with fluorescently labeled secondary antibodies (diluted in PBS with 0.25% BSA) and DAPI (0.1 g ml^−1^) in a humid chamber for 60 minutes in the dark after washing three times with PBS. After washing with PBS, slides were mounted with Fluoromount G (Dako, S3023). Human tissue sections were deparaffinized in xylene (10 min × 2) and rehydrated through a graded ethanol series (100%, 90%, 80% and 75%; 5 min each), followed by rinsing in distilled water. Antigen retrieval was performed in citrate buffer (pH 6.0) using microwave heating, followed by natural cooling to room temperature for 20 min. Endogenous peroxidase activity was blocked with 3% H_2_O_2_ in PBS for 10 min in the dark. After washing with PBS, sections were blocked with 20% goat serum in PBS containing 0.25% Triton X-100 for 30 min at room temperature and incubated overnight at 4 °C with A6 primary antibody (1:200). After washing with PBS, sections were incubated with HRP-conjugated secondary antibody (SouthernBiotech, 1:2,000) for 30 min at room temperature. Diaminobenzidine (DAB) development was performed for approximately 1 min (or until specific brownish staining appeared under microscopic observation) and stopped by rinsing in distilled water. Sections were counterstained with hematoxylin for 5 min, differentiated in 1% acid alcohol and blued under running tap water. Finally, sections were dehydrated, air-dried and mounted with neutral resin. For negative controls, staining was performed without primary antibodies or isotype controls. Stained sections were analyzed using a confocal laser scanning microscope (Leica, SP8 3X) or digital pathology slide scanner (KFBIO, KF-PRO-020). All images were prepared as TIFF files by ImageJ or Leica LAS-X (version 1.2) software and exported into Adobe IIIustrator CS6 for figure arrangements.

### Total cholesterol assay

Blood was collected into an EDTA pre-coated tube by using 1-ml syringes with a 23-gauge needle; anticoagulated blood samples from mice were centrifuged at 6,000 r.p.m. for 10 minutes; then the plasma was collected and used for the determination of total cholesterol according to the manufacturer’s instructions using the cholesterol quantitation kit (Sigma-Aldrich, MAK043-1KT).

### Statistical analyses

Data were analyzed using GraphPad Prism (version 10) or in R (version 4.2.1). The bar plots are presented as mean ± s.e.m. For comparisons of discrete variables, data distribution was tested by Shapiro−Wilk test. For data that followed a Gaussian distribution, Studentʼs *t*-test was used to compare difference between two groups, or a one-way ANOVA with Bonferroni post hoc test was used to perform statistical analysis among multiple groups. For repeated measurement data, such as body weight and antibody levels across multiple periods and among multiple groups, repeated-measures two-way ANOVA followed by a post hoc test was performed for analysis. As some datasets did not follow Gaussian distribution, the difference between two groups was compared by two-sided Wilcoxon rank-sum test, and difference among three or more groups was analyzed by non-parametric Kruskal−Wallis *H*-test with Dunn’s post hoc test for pairwise comparisons. For categorical variables, comparisons were formally analyzed by Pearson’s *χ*^2^ test and followed by post hoc pairwise comparisons, which conduct Fisher’s exact test for any of expected frequencies lower than 5 or *χ*^2^ test for all expected frequencies higher than 5. The Benjamini−Hochberg procedure was applied to adjust *P* values and control the FDR. Differences were considered significant at *P* < 0.05 (*P < 0.05, **P < 0.01, ***P < 0.001; not significant denoted by NS). No data were excluded from the analyses. Mice were randomized into different treatment groups with blinding to treament.

### Reporting summary

Further information on research design is available in the Nature Portfolio Reporting Summary linked to this article.

## Supplementary information

Supplementary Fig. 1.

Reporting Summary

Supplementary Tables 1−3 Supplementary Table 1. Immunoglobulin gene repertoire and reactivity of antibodies from WT and *Apoe*^−/−^ mice. Supplementary Table 2. Demographic and clinical characteristics of the study cohort (*n* = 495). Supplementary Table 3. Sensitivity analysis by including hsCRP as a covariate in the binary logistic regression model.

## Source data

Source Data Fig. 1 Statistical source data.

Source Data Fig. 2 Statistical source data.

Source Data Fig. 3 Statistical source data.

Source Data Fig. 4 Statistical source data.

Source Data Fig. 5 Statistical source data.

Source Data Fig. 6 Statistical source data.

Source Data Extended Data Fig. 3 Statistical source data.

Source Data Extended Data Fig. 5 Statistical Source Data.

Source Data Extended Data Fig. 7 Statistical source data.

Source Data Extended Data Fig. 8 Statistical source data.

Source Data Extended Data Fig. 9 Statistical source data.

## Data Availability

The BCR sequences and associated metadata for each sample, including mouse ID, tissue, genotype, mutation summary, isotype summary and clonality for BCR repertoire sequencing, as well as the count matrix and metadata (tissue, cluster, cell type and BCR-related information) for scRNA-seq data, and immunoprecipitation proteomics data, have been deposited in figshare (https://doi.org/10.6084/m9.figshare.27022876). The public mouse GC B cell data used in our study can be downloaded from the Gene Expression Omnibus under accession number GSE154634. We used publicly available software for the analyses. Software and parameters used are described in the Methods. All code and data are available in the main text or the supplementary materials.
