# Supplementary material for: Artery tertiary lymphoid organs encode a pathogenic high-affinity autoantibody−autoantigen pair in atherosclerosis
Source: Nat Cardiovasc Res. 2026 Sep 7;5(9):869–90. doi: 10.1038/s44161-026-00864-w (PMC13561875; doi:10.1038/s44161-026-00864-w)
Supplement: Supplementary file 1 [file 44161_2026_864_MOESM1_ESM.pdf]

nature cardiovascular research

Supplementary information

https://doi.org/10.1038/s44161-026-00864-w

Artery tertiary lymphoid organs encode a pathogenic high-affinity autoantibody–autoantigen pair in atherosclerosis

In the format provided by the authors and unedited

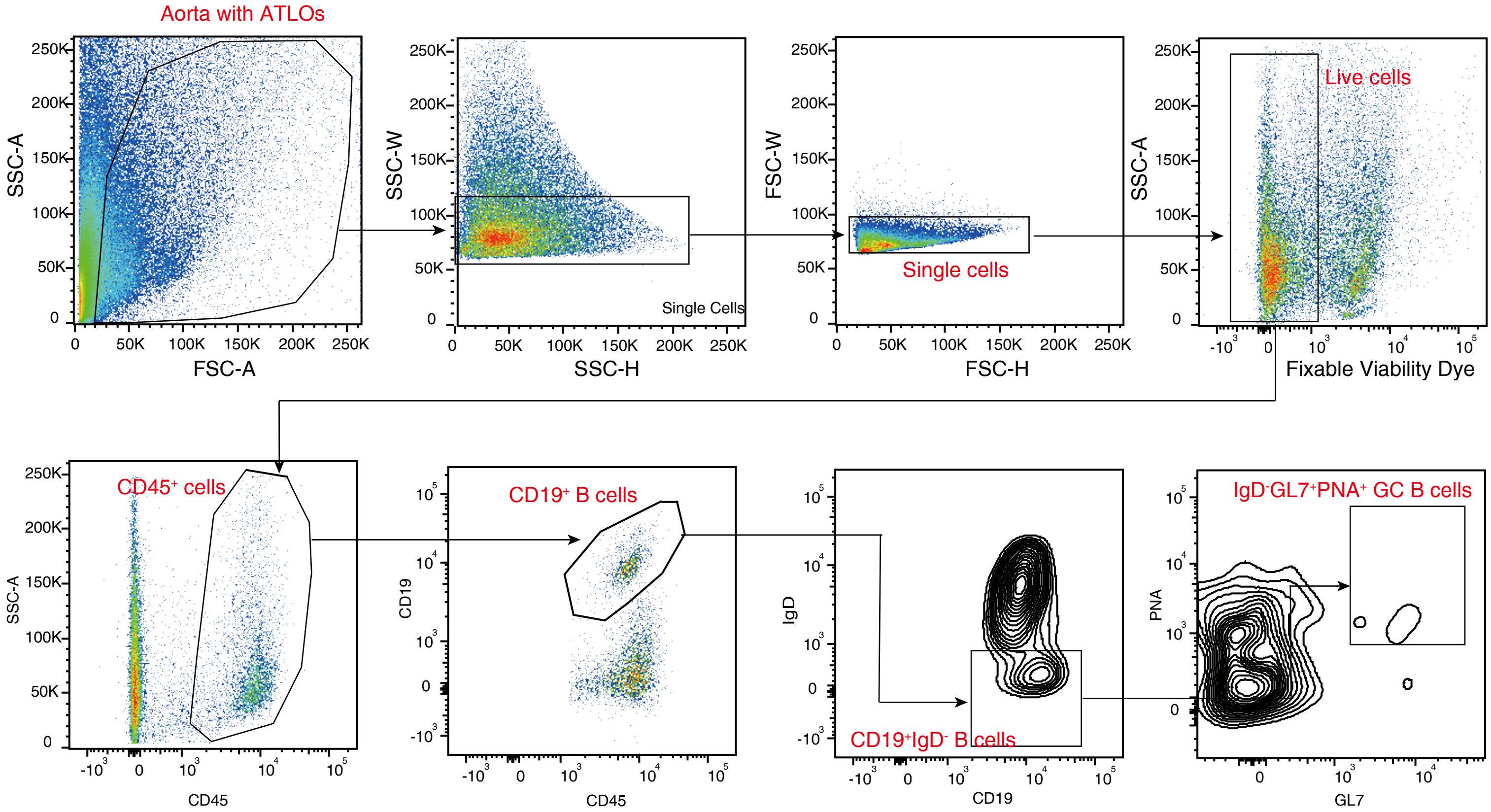

Aorta with ATLOs
SSC-A
FSC-A
SSC-W
SSC-H
Single Cells
FSC-W
FSC-H
Single cells
Live cells
Fixable Viability Dye
CD45⁺ cells
CD45
CD19⁺ B cells
CD19
IgD
CD19⁺IgD⁻ B cells
IgD⁻GL7⁺PNA⁺ GC B cells
PNA
GL7
