# Supplementary material for: Artery tertiary lymphoid organs encode a pathogenic high-affinity autoantibody−autoantigen pair in atherosclerosis
Source: Nat Cardiovasc Res. 2026 Sep 7;5(9):869–90. doi: 10.1038/s44161-026-00864-w (PMC13561875; doi:10.1038/s44161-026-00864-w)
Supplement: Supplementary file 2 [file 44161_2026_864_MOESM2_ESM.pdf]

nature portfolio

Corresponding author(s): Andreas J.R. Habenicht, Changjun Yin

Last updated by author(s): Jul 29, 2026

Reporting Summary

Nature Portfolio wishes to improve the reproducibility of the work that we publish. This form provides structure for consistency and transparency in reporting. For further information on Nature Portfolio policies, see our Editorial Policies and the Editorial Policy Checklist.

Statistics

For all statistical analyses, confirm that the following items are present in the figure legend, table legend, main text, or Methods section.

n/a	Confirmed	
☐	☒	The exact sample size (*n*) for each experimental group/condition, given as a discrete number and unit of measurement
☐	☒	A statement on whether measurements were taken from distinct samples or whether the same sample was measured repeatedly
☐	☒	The statistical test(s) used AND whether they are one- or two-sided<br>*Only common tests should be described solely by name; describe more complex techniques in the Methods section.*
☐	☒	A description of all covariates tested
☐	☒	A description of any assumptions or corrections, such as tests of normality and adjustment for multiple comparisons
☐	☒	A full description of the statistical parameters including central tendency (e.g. means) or other basic estimates (e.g. regression coefficient) AND variation (e.g. standard deviation) or associated estimates of uncertainty (e.g. confidence intervals)
☐	☒	For null hypothesis testing, the test statistic (e.g. *F*, *t*, *r*) with confidence intervals, effect sizes, degrees of freedom and *P* value noted<br>*Give P values as exact values whenever suitable.*
☒	☐	For Bayesian analysis, information on the choice of priors and Markov chain Monte Carlo settings
☐	☒	For hierarchical and complex designs, identification of the appropriate level for tests and full reporting of outcomes
☐	☒	Estimates of effect sizes (e.g. Cohen's *d*, Pearson's *r*), indicating how they were calculated

Our web collection on statistics for biologists contains articles on many of the points above.

Software and code

Policy information about availability of computer code

Data collection	For single cell sorting:BD FACSAria™ III, BD Biosciences.<br>For gel bead in emulsion preparation: 10x chromium controller<br>For library size measurement: fragment analyzer, Advanced Analytical Technologies GmbH<br>For Single cell data analysis: Linux system (Intel(R) Xeon(R) E5-1650 v3 processor, Cores: 6, Threads: 12) or Windows system (64-bit operating system, x64-based processor)<br>For sanger seqnecning: ABI 3730 capillary sequencers<br>For NGS sequencing: NextSeq 500, MiSeq or NovaSeq 6000, Illumina<br>For FACS data analysis: FACSCanto IITM<br>For immunofluorescence staining: Leica SP8 3X, Fiji<br>For antibody-antigen binding affinity: SPR, BIAcore X 100 (Cytiva, Freiburg, Germany) equipped with a research-grade C1 sensor chip; Biolayer Interferometry (BLI), OCTET R8 (SARTORIUS, Otto-Brenner-Str. 2037079 Göttingen, Germany) equipped with research-grade Protein A Biosensors (18-5010, SARTORIUS)<br>For ELISA: Multiskan SkyHigh (Thermo Fisher Scientific, USA)<br>For Mass spectrometry: Ultimate 3000 RSLCnano (Dionex, Germering, Germany)
Data analysis	For single cell RNA sequencing and single cell BCR sequencing analysis: The sequencing raw base call (BCL) files were demultiplexed with Cell Ranger (version 7.0.1) using the Linux system (Intel(R) Xeon(R) E5-1650 v3 processor, Cores: 6, Threads: 12). For the 5' gene expression sequencing data, reads were aligned to mouse mm10 reference transcriptome using STAR aligner in the 'cellranger count' or the 'cellranger multi' function. The output raw barcodes, features, and matrix files after running Cell Ranger were loaded into RStudio (R version 4.2.1) and processed with Seurat (version 4.3.0) package. Cells contained fewer than 200 genes, higher than 4,000 detected genes or > 8% of mitochondrial genes were filtered out as suspected doublets or debris. Data from different tissues were log-normalized, and 2,000 highly

variable features were calculated to integrate data. After integration, principal component analysis (PCA) was performed for following dimensional reduction and clustering. 20 dims were included to reduce the dimension and assign clusters. The clustering result was visualized using uniform manifold approximation and projection (UMAP) dimensionality reduction methods built in Seurat. In order to focus on B subsets, clusters highly expressed Cd19 or Sdc1 (CD138) genes were extracted and re-clustered for the following analyses. GC B cells were extracted and integrated with published data (GSE154634) following the integration pipelines provided by the Seurat package. 0.2 resolution was introduced to nonsupervisory cluster all integrated GC B cells. The phase of cell cycle was annotated by calculating the expression of cell cycle-related markers provided by Tirosh et al. using the cell cycle scoring pipelines in the Seurat package. PCs were extracted to be re-clustered base on 0.6 resolution. The BCR sequences were assembled and annotated referring to GRCm38 reference. The information of BCR sequences in each B cell was integrated into metadata of 5' gene expression data for further analysis. Differentially expressed genes (DEGs) between clusters were calculated by 'FindMarkers' or 'FindAllMarkers' function in Seurat, genes with fold change (FC) > 0.25 and adjusted p < 0.05 were considered as significant DEGs. Cell-cell interaction was performed with CellPhoneDB (version 5) package. The count of cell-cell interactions was predicted and visualized with CellChat (version 2.1.2) package. The normalized expression of DEGs in each cell was visualized with DoHeatmap function built in the Seurat package. DEGs obtained from each B cell subsets were parsed to the clusterProfiler package for functional enrichment and multiple comparisons. Terms showing a false discovery rate (FDR) less than 0.05 were considered as significant. Selected enriched terms were illustrated by ggplot2. The results of gene set enrichment analysis (GSEA) were also obtained using the clusterProfiler package and the GSEA plot for selective GO term was visualized by the enrichplot package.
For BCR V(D)J repertoire analysis: Reads were aligned to the GRCm38-3.1.0 reference transcriptome using the 'cellranger vdj' or "cellranger multi"command to perform sequence assembly and paired clonotype calling in Cell Ranger. Unique BCRs shared common IGHV family, IGHJ family as well as identical CDR3 amino acid (CDR3aa) region were considered the same clonotype. If one clonotype with two cells or more than two cells, it was considered as expanded clonotype. Sequences of each unique BCR sequence were re-aligned with the IMGT/HighV-QUEST database to summary the number of mutations in V region. The isotype of each BCR heavy chain sequence was annotated by MiXCR for Rep-seq data or by Cell Ranger for scRNA-seq data.
For mass spectrometry data analysis: MS/MS data were processed using MaxQuant. The label-free quantification (LFQ) intensities for each replicate and control sample were used for downstream analysis of the A6-targeted antigen. Specifically, the relative LFQ intensity for each replicate was calculated by normalizing against the control sample. Proteins with a relative LFQ intensity greater than 1 (indicating enrichment in the replicates compared to the control) were identified for each replicate. A Venn diagram was then used to visualize the overlap of enriched potential target proteins across the replicates.
For antibody-antigen binding affinity data analysis: BIAcore X 100 (Cytiva, Freiburg, Germany) equipped with a research-grade C1 sensor chip (surface plasmon resonance mthod, SPR) and OCTET R8 (SARTORIUS, Otto-Brenner-Str. 2037079 Göttingen, Germany) equipped with research-grade Protein A Biosensors (18-5010, SARTORIUS) (bio-layer interferometry method, BLI) were both used to determine the antibody-antigen binding affinity. The data were processed with BIACORE X 100 evaluation 2.0 software and Octet analysis studio software for SPR and BLI output data, respectively. ELISA was also used to determine the binding affinity between antibody and antigen. The optical density (OD) of each reaction was measured using a Multiskan SkyHigh (Thermo Fisher Scientific, USA) with a detection wavelength of 450 nm.
For mouse in vivo experiment: The body weight of each group mice was measured. The blood was collected through the tail vein to measure the titer of antibodies and cholesterol levels. The antibody levels were determined by ELISA as described above. The plasma cholesterol levels were measured by using the cholesterol quantitation kit (Sigma, MAK043-1KT). The whole aorta and aortic root were collected to analyze the atherosclerosis development. En-face Sudan IV and Oil-red-O (ORO) staining were used to analyse the lipid accumulation of mouse aortas. To assess the percentages of lipid in the whole aorta, arch, thoracic, and abdominal parts, a digital camera (Leica) was used to capture images with a standard bar. Oil-red-O (ORO) staining was used to analyse atherosclerotic plaque areas. Thunder imaging system (Leica) was used to capture ORO staining images. Image J was used to measure the percentages of lipid deposition in the whole aorta, arch, thoracic, and abdominal parts and quantify atherosclerotic lesion areas.
For immunofluorescence staining: For negative controls, stainings were performed without primary antibodies or isotype controls. Stained sections were analyzed using a Leica SP8 3X (Leica microsystems, Germany). Images were acquired with identical microscope settings using sequential channel acquisition to avoid cross-talk between fluorophores. All images were prepared as TIF files by Fiji (ImageJ, NIH, open source) or Leica LAS-X (V3.5) software and exported into Adobe IIlustrator CS6 for figure arrangements.
For clinical cohort study: we performed a cross-sectional study of 495 individuals of the general population who undergoing routine medical screening of preventive health care at the First Affiliated Hospital of Sun Yat-sen University (FAH-SYSU), China. The study was approved by the Ethics Committee of the FAH-SYSU, and all participants provided their informed consent for participation. The baseline demographic and clinical characteristics of the participants are summarized in Supplementary Table S2. Exclusion criteria included patients with diagnosed cardiovascular diseases, strokes, autoimmune diseases requiring systemic treatment, active malignancies, and severe kidney or liver dysfunction. Blood samples were collected to assess general clinical laboratory parameters and serum titers of anti-H2B antibody. Additionally, as part of medical screening program, chest 3-dimentional computed tomography (CT) scans were performed voluntarily for general diagnostic purposes. The primary outcome was the presence of aortic abnormalities related to atherosclerosis, by the terms "aortic atherosclerosis," "aortic sclerosis," "sclerosis of the aorta," and "aortic hardening" in the CT report. This diagnosis was further confirmed by the presence of high-density signals within aorta or coronary arteries in CT scans, which directly measure the calcification of aorta and arteries. Valid data were available for 418 individuals. The association between the clinical outcome and anti-H2B antibody titer was analyzed using binary logistic regression models. The model included log-transformed anti-H2B antibody titer (to approximate Gaussian distribution), sex, age, body mass index (BMI), systolic blood pressure, diastolic blood pressure, HDL cholesterol, LDL cholesterol, triglycerides, estimated glomerular filtration rate (eGFR), homocysteine, and hemoglobin A1c (HbA1c). Total cholesterol levels were excluded due to significant collinearity with LDL cholesterol. The results of the simultaneous-entry model were confirmed using a backward logistic regression approach. In this model, sex, age, anti-H2B antibody titer, LDL cholesterol, and HbA1c were retained in the final step as independent predictors.
Statistical analysis: Data were analyzed using GraphPad Prism (version 10) or in R (version 4.2.1). The bar plots were presented as mean ± standard error of mean (SEM). For comparisons of discrete variables, data distribution was tested by Shapiro–Wilk test. For data that followed a Gaussian distribution, student T-test was used to compare difference between two groups or a one-way analysis of variance (ANOVA) with Bonferroni post hoc test was used to perform statistical analysis among multiple groups. For repeated measurement data, such as body weight and antibody levels across multiple periods and among multiple groups, repeated measures two-way ANOVA followed by a post hoc test was performed for analysis. As some datasets did not follow Gaussian distribution, the difference between two groups was compared by two-sided Wilcoxon rank-sum test and difference between three or more groups was analyzed by non-parametric Kruskal–Wallis H test with Dunn's post hoc test for pairwise comparisons. For categorical variables, comparisons were formally analyzed by Pearson's Chi-square test and followed by post hoc pairwise comparisons, which conduct Fisher's exact test for any of expected frequencies < 5 or Chi-square test for all expected frequencies > 5. The Benjamini–Hochberg procedure was applied to adjust p values and control the false discovery rate. Differences were considered significant at a two-tailed P value<0.05. *: P <0.05; **: P <0.01; ***: P <0.001; n.s.: not significant.

For manuscripts utilizing custom algorithms or software that are central to the research but not yet described in published literature, software must be made available to editors and reviewers. We strongly encourage code deposition in a community repository (e.g. GitHub). See the Nature Portfolio guidelines for submitting code & software for further information.

Data

Policy information about availability of data

All manuscripts must include a data availability statement. This statement should provide the following information, where applicable:

- Accession codes, unique identifiers, or web links for publicly available datasets
- A description of any restrictions on data availability
- For clinical datasets or third party data, please ensure that the statement adheres to our policy

The scRNA-seq and scBCR-seq data discussed in this publication are deposited in Figshare (https://doi.org/10.6084/m9.figshare.27022876) . The public mouse scRNA-seq data (GSE154634) were download from GEO database.

Research involving human participants, their data, or biological material

Policy information about studies with human participants or human data. See also policy information about sex, gender (identity/presentation), and sexual orientation and race, ethnicity and racism.

Reporting on sex and gender	In our clinical cohort study, we initially recruited 495 participants, comprising 303 males and 192 females. After applying exclusion criteria, 418 participants remained for data analysis, consisting of 251 males and 167 females.
Reporting on race, ethnicity, or other socially relevant groupings	All participants are Han Chinese
Population characteristics	The baseline demographic and clinical characteristics of the participants are summarized in Supplementary Table S2.
Recruitment	A cross-sectional study of 495 individuals of the general population who undergoing routine medical screening of preventive health care at the First Affiliated Hospital of Sun Yat-sen University (FAH-SYSU), China. Exclusion criteria included patients with diagnosed cardiovascular diseases, strokes, autoimmune diseases requiring systemic treatment, active malignancies, and severe kidney or liver dysfunction.
Ethics oversight	We performed a cross-sectional study of 495 individuals of the general population (Registration number: ChiCTR2400089577) who undergoing routine medical screening of preventive health care at the First Affiliated Hospital of Sun Yat-sen University (FAH-SYSU), China. The study was approved by the Clinical Research and Laboratory Animal Ethics Committee with Approval ID: [2024]374, The First Affiliated Hospital of Sun Yat-sen University, and all participants provided their informed consent for participation.

Note that full information on the approval of the study protocol must also be provided in the manuscript.

Field-specific reporting

Please select the one below that is the best fit for your research. If you are not sure, read the appropriate sections before making your selection.

☒ Life sciences ☐ Behavioural & social sciences ☐ Ecological, evolutionary & environmental sciences

For a reference copy of the document with all sections, see nature.com/documents/nr-reporting-summary-flat.pdf

Life sciences study design

All studies must disclose on these points even when the disclosure is negative.

Sample size	For our scRNA-seq and scBCR-seq experiments, the number of mice pooled per sample was determined based on preliminary FACS experiments, ensuring sufficient cell yield for high-quality sequencing data. In vivo animal experiments were designed with statistical power calculations to determine the appropriate sample size. Where feasible, preliminary studies were conducted to further refine the number of animals required, adhering to local ethical guidelines and minimizing animal use in accordance with reductionist principles. The sample sizes for each experiment are provided in the figure legends and detailed in the Methods section.
Data exclusions	For scRNA-seq and scBCR-seq data, cells expressing fewer than 200 genes, more than 4,000 genes, or with >8% mitochondrial genes were excluded as potential doublets or debris.<br>In vivo animal experiments excluded mice that failed to achieve successful immunization or exhibited abnormal tissue damage. Blood samples with hemolysis was not included.<br>For the human clinical cohort study, patients diagnosed with cardiovascular diseases, stroke, autoimmune diseases requiring systemic treatment, active malignancies, or severe kidney or liver dysfunction were excluded from the study.
Replication	All replication attempts were successful. For the scRNA-seq and scBCR-seq analyses, two separate cohort studies were conducted: one using a pooled sample strategy and the other utilizing hashtag labeling. Both approaches ensured data replication and stability. In the in vivo animal studies, at least three biological replicates were used to guarantee reproducibility. The human clinical cohort study involved a large-scale participant group, including both individuals with and without atherosclerosis.

Randomization	samples and animals were assigned to groups randomly.
Blinding	Mice samples were examined in a blinding and randomization way.

Reporting for specific materials, systems and methods

We require information from authors about some types of materials, experimental systems and methods used in many studies. Here, indicate whether each material, system or method listed is relevant to your study. If you are not sure if a list item applies to your research, read the appropriate section before selecting a response.

Materials & experimental systems

n/a	Involved in the study
☐	☒ Antibodies
☒	☐ Eukaryotic cell lines
☒	☐ Palaeontology and archaeology
☐	☒ Animals and other organisms
☐	☒ Clinical data
☒	☐ Dual use research of concern
☒	☐ Plants

Methods

n/a	Involved in the study
☒	☐ ChIP-seq
☐	☒ Flow cytometry
☒	☐ MRI-based neuroimaging

Antibodies

Antibodies used	For single cell sorting (scRNA-seq and scBCR-seq): CD45.2[104] (APC,Biolegend,109814) CD45[30-F11] (Percp-cy5.5, Bioscience, 45-0451-82); Fixable Viability Dye(V500, invitrogen,65-0866-14) ; biolegend anti-mouse totalseq-c Hashtag Antibody[CD45:clone 30-F11; MHC I: clone:M1/42] (C0301, C0302, C0303, C0304; cat#:155861, 155863, 155865, 155867). CD16/32[93] (eBioscience,14-0161-82)<br>For immunostaining: ATLO GC-derived, RLN GC-derived antibodies (in-house design and company synthesized), anti-mouse CD68 (FA11, Serotec), alpha smooth muscle actin (SMA, 1A4, SIGMA), CXCR4(UMB2, Abcam), RelB (C-19, Santa Cruz Biotechnology), c-MyC (Y69, Abcam), CD35 (8C12, Biosciences), and BCL6 (K112-91, Biosciences);<br>For single GC B cell isolation: FVD (APC-Cy7-conjugated, 1:1000, eBioscience), CD45 PercP-Cy5.5 (30-F11, eBioscience), CD19 APC (1D3, eBioscience), IgD-eFluor 450 (11-26c, eBioscience), GL7-Biotin (GL7, eBioscience), PNA-FITC (Vector Lab), Streptavidin-APC-eFluor780 (eBioscience).<br>For collecting CD19+ B cells: FVD(APC-Cy7-conjugated, 1:1000 dilution, eBioscience), anti-CD45 (PerCP/Cy5.5, 1:200 dilution, clone 30-F11, eBioscience), anti-CD19 (APC, 1:200 dilution, clone eBio1D3, eBioscience).<br>For in vivo animal experiments, SPR, BLI and ELISA studies: ATLO GC-derived, RLN GC-derived antibodies (in-house design and company synthesized)
Validation	All commercial antibodies were purchased from the above mentioned companies, and the relevant validation information is available on their website. Additionally, these antibodies have been widely used and validated in our lab through previous studies. All the in-house design and company synthesized antibodies were validated by the company.

Animals and other research organisms

Policy information about studies involving animals; ARRIVE guidelines recommended for reporting animal research, and Sex and Gender in Research

Laboratory animals	For scRNA-seq, scBCR-seq, and scBCR cloning experiments: ApoE-/- mice with C57BL/6 background were bred and housed in the specific pathogen-free animal facility of Munich University with a 12 h light/dark cycle. Mice used in this project were more than 78 weeks old, fed on a normal chow diet and without external interferences during the experiments. C57BL/6 mice raised under the same conditions were used as the control group in our study.<br>For A6 antibody and H2B treatment experiments, 8-10 weeks Apoe-/- mice were used for our study.<br>For AAV-PCSK9 induced atherosclerosis, 8-week-old male C57BL/6 mice were injected with AAV-PCSK9 (AAV8-PCSK9-D377Y, Vigene Biosciences) via the tail vein at a dose of 1.5x1011 vector genomes per mouse. AV-PCSK9 injected mice were fed with high fat diat.
Wild animals	This study did not involve wild animals.
Reporting on sex	Only male mice were used in our study.
Field-collected samples	This study did not involve samples collected from the field.
Ethics oversight	The animal procedures were authorized by the Regierung of Oberbayern or by the First-affiliated Hospital of Sun Yat-sen University, following the guidelines of the local Animal Use and Care Committee and the National Animal Welfare Laws.

Note that full information on the approval of the study protocol must also be provided in the manuscript.

Clinical data

Policy information about clinical studies

All manuscripts should comply with the ICMJE guidelines for publication of clinical research and a completed CONSORT checklist must be included with all submissions.

Clinical trial registration: ChiCTR2400089577

Study protocol: Total 495 individuals of the general population who undergoing routine medical screening of preventive health care at the First Affiliated Hospital of Sun Yat-sen University (FAH-SYSU), China. The study was approved by the Clinical Research and Laboratory Animal Ethics Committee with Approval ID: [2024]374, The First Affiliated Hospital of Sun Yat-sen University, and all participants provided their informed consent for participation. The baseline demographic and clinical characteristics of the participants are summarized in Extended data Table 2. Exclusion criteria included patients with diagnosed cardiovascular diseases, strokes, autoimmune diseases requiring systemic treatment, active malignancies, and severe kidney or liver dysfunction. Blood samples were collected to assess general clinical laboratory parameters and serum titers of anti-H2B antibody. Additionally, as part of medical screening program, chest 3-dimentional computed tomography (CT) scans were performed voluntarily for general diagnostic purposes. The primary outcome was the presence of aortic abnormalities related to atherosclerosis, by the terms “aortic atherosclerosis,” “aortic sclerosis,” “sclerosis of the aorta,” and “aortic hardening” in the CT report. This diagnosis was further confirmed by the presence of high-density signals within aorta or coronary arteries in CT scans, which directly measure the calcification of aorta and arteries. Valid data were available for 418 individuals. The association between the clinical outcome and anti-H2B antibody titer was analyzed using binary logistic regression models. The model included log-transformed anti-H2B antibody titer (to approximate Gaussian distribution), sex, age, body mass index (BMI), systolic blood pressure, diastolic blood pressure, HDL cholesterol, LDL cholesterol, triglycerides, estimated glomerular filtration rate (eGFR), homocysteine, and hemoglobin A1c (HbA1c). Total cholesterol levels were excluded due to significant collinearity with LDL cholesterol. The results of the simultaneous-entry model were confirmed using a backward logistic regression approach. In this model, sex, age, anti-H2B antibody titer, LDL cholesterol, and HbA1c were retained in the final step as independent predictors.

Data collection: demographic and clinical characteristics of the participants were collected

Outcomes: The primary outcome was the presence of aortic abnormalities related to atherosclerosis, by the terms “aortic atherosclerosis,” “aortic sclerosis,” “sclerosis of the aorta,” and “aortic hardening” in the CT report. This diagnosis was further confirmed by the presence of high-density signals within aorta or coronary arteries in CT scans, which directly measure the calcification of aorta and arteries.

Plants

Seed stocks: *Report on the source of all seed stocks or other plant material used. If applicable, state the seed stock centre and catalogue number. If plant specimens were collected from the field, describe the collection location, date and sampling procedures.*

Novel plant genotypes: *Describe the methods by which all novel plant genotypes were produced. This includes those generated by transgenic approaches, gene editing, chemical/radiation-based mutagenesis and hybridization. For transgenic lines, describe the transformation method, the number of independent lines analyzed and the generation upon which experiments were performed. For gene-edited lines, describe the editor used, the endogenous sequence targeted for editing, the targeting guide RNA sequence (if applicable) and how the editor was applied.*

Authentication: *Describe any authentication procedures for each seed stock used or novel genotype generated. Describe any experiments used to assess the effect of a mutation and, where applicable, how potential secondary effects (e.g. second site T-DNA insertions, mosiacism, off-target gene editing) were examined.*

Flow Cytometry

Plots

Confirm that:

☒ The axis labels state the marker and fluorochrome used (e.g. CD4-FITC).

☒ The axis scales are clearly visible. Include numbers along axes only for bottom left plot of group (a 'group' is an analysis of identical markers).

☒ All plots are contour plots with outliers or pseudocolor plots.

☒ A numerical value for number of cells or percentage (with statistics) is provided.

Methodology

Sample preparation: ATLOs and plaques were cut into small pieces and digested with enzyme cocktail (400 U/ml collagenase type I, 10 U/ml collagenase type XI, 60 U/ml hyaluronidase, 60 U/ml DNase I, and 20 mM HEPES in DPBS) for 40 mins at 37° C with slow shaking. Cell suspensions were filtered through a 100 μm strainer and rinsed with pre-cooled FACS buffer. RLNs and spleen were cut into small pieces and mashed on the 100 μm strainer with the plunger of syringe, then rinsed with pre-cooled FACS buffer.

Instrument: FACSCanto IITM (BD Bioscience)

Software	FlowJo (BD Bioscience)
Cell population abundance	Dose not apply.
Gating strategy	For all cell types, initial forward scatter versus side-scatter gates were adjusted to include all cells and exclude cell debris, dead cells were excluded using Fixable Viability Dye (eBioscience) before gating for leukocytes followed by gating out cell doublets. For scRNA-seq and scBCR-seq experiments, total live immune cells (FVD-CD45+) were gated for cell sorting. For single GCB cell isolation: FVD-CD45+CD19+IgD-GL7+PNA+ GCB cells were sorted. To collect CD19+ B cells, FVD-CD45+CD19+ B cells were collected. Representative gating strategy in supplementary Figure 1.

☒ Tick this box to confirm that a figure exemplifying the gating strategy is provided in the Supplementary Information.
